# Brillouin Microscopy of Breast tumor Spheroids On‐a‐Chip: Mechanical and Transcriptional Responses to Microfluidic‐Induced Rapid Deformations

**DOI:** 10.1002/advs.202513153

**Published:** 2025-12-16

**Authors:** Mona Makkieh, Alessandra Anna Passeri, Davide Lazzari, Stefano Marchesi, Andrea Disanza, Rafal Khaled Ahmad Salem, Francesco Bonacci, Camillo Mazzella, Emanuele Martini, Mattia Tonani, Leonardo Donati, Edoardo Bellini, Hind Abdo, Judith Pineau, Serena Magni, Fabrizio Orsenigo, Matthieu Piel, Daniele Fioretto, Sabata Martino, Maurizio Mattarelli, Giorgio Scita, Brenda J. Green, Silvia Caponi

**Affiliations:** ^1^ IFOM ETS – The AIRC Institute of Molecular Oncology Via Adamello, 16 Milan 20139 Italy; ^2^ Department of Physics and Geology University of Perugia Via A. Pascoli Perugia 06123 Italy; ^3^ Department of Oncology and Haemato‐Oncology University of Milan Milan 20122 Italy; ^4^ Department of Chemistry Biology and Biotechnologies University of Perugia Via del Giochetto Perugia 06126 Italy; ^5^ CNRS UMR144 Institut Curie Institut Pierre Gilles de Gennes PSL Research University Paris 75005 France; ^6^ Centro di Eccellenza Materiali Innovativi Nanostrutturati per Applicazioni Chimiche Fisiche e Biomediche (CEMIN) University of Perugia Perugia 06123 Italy; ^7^ Consiglio Nazionale delle Ricerche CNR ‐ Istituto Officina dei Materiali (IOM) c/o Department of Physics and Geology University of Perugia Via A. Pascoli Perugia 06123 Italy

**Keywords:** breast cancer, Brillouin microscopy, fluorescence imaging, microfluidics, Raman microscopy, spheroids

## Abstract

Pioneering a novel integrated photonic platform, Brillouin–Raman Microspectroscopy on‐a‐Chip is presented, which enables real‐time, high resolution analysis of mechano‐chemical dynamics in complex 3D biological systems under controlled deformation. This contact‐free, all‐optical method seamlessly combines Brillouin and Raman microscopy within microfluidic devices, providing a unique correlative approach to overcome current limitations in probing cellular mechanics and biochemical responses in live multicellular models. This innovative platform, when applied to breast tumor spheroids, revealed that rapid, cyclical mechanical deformations, mimicking in vivo stresses, profoundly impacts tumor physiology. These findings demonstrate that controlled deformations trigger rapid nuclear shape changes and robust transcriptional reprogramming, marked by a significant (48‐fold) upregulation of the early stress response regulator ATF3. These responses are accompanied by global spheroid stiffening, as precisely quantified by Brillouin spectroscopy. Remarkably, repeated deformation imprints a form of mechanical memory in these collective systems, which culminates in enhanced collagen invasion over 24 h. The label‐free methodology sets a new benchmark in biophotonics, unlocking a new class of experiments to probe and modulate physiological and pathological processes with transformative potential for cancer research, broader mechanobiology, and the study of mechanical memory in organoids and other complex 3D models.

## Graphical abstract

Brillouin‐Raman Microspectroscopy on‐a‐Chip (BRmS) enables real‐time analysis of breast tumor spheroids in microfluidic channels. Following cyclical deformations, MCF10.DCIS.com spheroids are captured in recovery traps and imaged with BRmS. The applied deformations trigger a stiffening response marked by increased frequency shifts, elevation of the master transcriptional regulator ATF3, and enhanced collagen invasion.

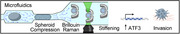

## 1 Introduction

Mechanical forces and physical properties are essential in regulating cellular processes, including cytoskeletal remodeling, gene expression, and cell behavior, with significant implications for both physiological and pathological conditions.^[^ ^1^, ^2^, ^3^, ^4^ ^]^ Mechanics governs the interplay between the forces acting on the cells and their deformation, triggering the activation of biochemical signaling pathways. This interplay drives the cellular behavior, disease progression, and it is particularly relevant in the context of cancer.^[^ ^1^, ^2^, ^5^, ^6^ ^]^ The emerging field of mechanobiology sheds light on the complex mechanisms by which cells sense and respond to forces, offering new perspectives in physiology and disease progression. Despite the relevance of the field and the advancements in the characterization of 3D cell mechanics, significant challenges persist.

Several non‐contact techniques probe mechanics in 3D. Optical tweezers/optical stretchers apply calibrated piconewton– nanonewton forces with exquisite control, but typically require isolated cells or exogenous handles and are low‐throughput and difficult to deploy deep inside compact spheroids.^[^ ^7^ ^]^ Magnetic manipulation likewise utilizes bead internalization or receptor coupling, making force transmission and targeting in densely packed aggregates uncertain.^[^ ^8^ ^]^ Ultrasound/acoustic elastography achieves large penetration depths but at coarse spatial resolution (tens of µm to mm), reporting bulk tissue properties rather than single‐cell or supracellular mechanics.^[^ ^9^ ^]^ Microfluidic assays (including viscosity‐ modulated flows) offer high throughput but offer very limited deformability and place spheroids non‐ native highly viscous solutions.^[^ ^10^ ^]^

In this context, Brillouin microscopy (BM) is emerging as a breakthrough technology.^[^ ^11^, ^12^ ^]^ It probes the mechanical properties of samples using light, relying on the interaction between a monochromatic laser beam and the high frequency (GHz) acoustic waves spontaneously present in the material. The velocity and attenuation of these acoustic waves depend on the local mechanical properties of the system. As a result, the light inelastically scattered by the sample carries information about these properties, which can be obtained from the frequency shift (ω_*B*_) and linewidth (Γ) of the Brillouin spectrum.^[^ ^13^, ^14^ ^]^

The backscattering geometry is often used in high resolution microscopy configurations. In these geometries, the square of the Brillouin frequency shift ω_B_ is proportional to the longitudinal storage elastic modulus M'.^[^ ^14^ ^]^ Hence, both M' or ω_B_ can be used as a proxy for microscale mechanics. Due to these capabilities, BM has been successfully applied in different experimental systems, including the analysis of the mechanical modulation of subcellular compartments in living cells at the onset of several diseases,^[^ ^15^, ^16^, ^17^, ^18^ ^]^ and the characterization of 3D structures in tumor spheroids and in tissues, even in living embryos or turbid samples.^[^ ^19^, ^20^, ^21^, ^22^, ^23^, ^24^ ^]^ Collectively, these studies demonstrate the capability of this imaging technique to provide micro‐mechanical insights of complex biological systems, establishing BM as a valuable tool for biomedical research.

The comparison between M', obtained by Brillouin microscopy, and the Young's modulus (E), often used to quantify microscale stiffness, is not direct. A positive correlation between E and M' has been reported in specific cases; however, the proposed phenomenological link remains strictly sample dependent.^[^ ^12^, ^18^ ^]^ Both moduli describe the response to uniaxial stress along the loading axis; yet, they capture the materials’ deformability under different conditions. M' principally reflects the materials’ resistance to volume changes, whereas the Young's modulus (E) also takes into account its resistance to shape changes.^[^ ^12^ ^]^ This distinction is particularly relevant in highly hydrated soft materials, in which the water, being nearly incompressible but easily deformable, strongly influences the mechanical behavior.^[^ ^25^ ^]^ This consideration explains the orders‐of‐magnitude differences often observed in biological materials between the two moduli.

In this study, we used Brillouin microscopy (BM), which is a non‐contact and non‐destructive technique, thus it enables real‐time measurements directly within the native environment of spheroids. This method uses high magnification objectives and allows mechanical characterization with subcellular spatial resolution. Moreover, as a microscopy‐based technique, BM integrates seamlessly with complementary methods such as fluorescence^[^ ^22^ ^]^ or Raman microscopy,^[^ ^15^, ^26^, ^27^ ^]^ further enhancing its versatility and potential for multi‐modal analysis. In particular, the Brillouin and Raman microSpectroscopy (BRmS) combines mechanical and chemical characterization of micro‐structured samples, demonstrating great potential in biology, medicine and materials science.^[^ ^15^, ^28^, ^29^ ^]^ This combined approach has already been demonstrated to follow the correlation between chemical composition and mechanical properties at microscale levels in heterogeneous samples such as polymeric films,^[^ ^29^ ^]^ biofilms.^[^ ^26^ ^]^ ex vivo human tissues^[^ ^28^, ^30^ ^]^ and more recently in single cells to account for how water content affects cells and nuclear mechanics.^[^ ^18^, ^25^ ^]^

Here, we present a novel photonic platform‐ BRmS‐on‐chip ‐ that further extends the capabilities of BRmS through its integration with microfluidic devices. This innovative approach addresses a key unmet challenge, enabling real‐time investigation of tumor cell aggregate responses to controlled mechanical stimuli. Microfluidic devices are designed to provide precise control over experimental conditions, allowing the modulation of micro‐environmental factors and stresses. Several of these techniques have analyzed the deformability of single cells through narrow constrictions, such as real time deformability cytometry, constriction‐based deformability cytometry and extensional flow deformability.^[^ ^31^, ^32^ ^]^ In a single cell microfluidic assay, the shear creep and the cell protrusion time into the channel were related to E, considering a power‐law rheology model to describe the viscoelastic behaviors. This approach provides a system to quantitatively probe the deformability of individual cells during flow.^[^ ^33^ ^]^ However, there are very few studies that developed devices to study the impact of controlled deformation and mechanical stress on cell collectives, such as tumor aggregates, spheroids or organoids.^[^ ^34^, ^35^, ^36^, ^37^, ^38^ ^]^ This critical gap is particularly relevant in cancer biology, as tumor cells often invade and extravasate from the primary tumor as collective entities.^[^ ^2^ ^]^ Tumor cell clusters in the circulation, for example, have a survival advantage and possess enhanced protection against shear stress. These multicellular aggregates can exhibit a 50‐fold greater metastatic potential compared to single cells.^[^ ^39^ ^]^ However, a causal link between the biophysical adaptability and properties of tumor cell collectives and their metastatic potential remains undefined. To address this gap, we developed an innovative method to probe the properties of cell aggregates during and after controlled perturbative conditions. Using custom‐designed deformability devices, we subjected MCF10.DCIS.com breast tumor spheroids to controlled rapid deformations and monitored their shape recovery in real time with Brillouin and Raman microscopy. We selected MCF10.DCIS.com cells as a clinically relevant model of ductal carcinoma in situ (DCIS), as they recapitulate key features of early‐stage breast cancer and can transition into invasive carcinoma via collective invasion.^[^ ^40^ ^]^ For the first time, this novel experimental platform is used to study how deformations impact nuclear shape, viscoelastic properties, transcriptional changes and invasive potential.

## 2 Results and Discussion

This section introduces the microfluidic devices and their capabilities to measure relative stiffness, comparing results with those obtained from Brillouin microscopy (BM) and Raman analysis. We developed four complementary devices: The deformation device was used to assess spheroid deformability and transit dynamics under repetitive constrictions. The flow control device is a version of the deformation device without constrictions, to control for shear stress effects. The long‐channel device was designed to impose sustained compression on spheroids. Finally, the recovery device represents a modified deformation device incorporating downstream capture zones, which allow us to monitor post‐deformation shape and mechanical recovery of spheroids. This sequential design: from constriction to sustained compression and recovery enabled us to systematically dissect spheroid mechano‐adaptation using BRmS‐on‐chip under progressively complex stress conditions. Finally, the biological effects of these perturbations are investigated.

### 2.1 Design and Characterization of the Spheroid Deformation Device

**Figure 1:**
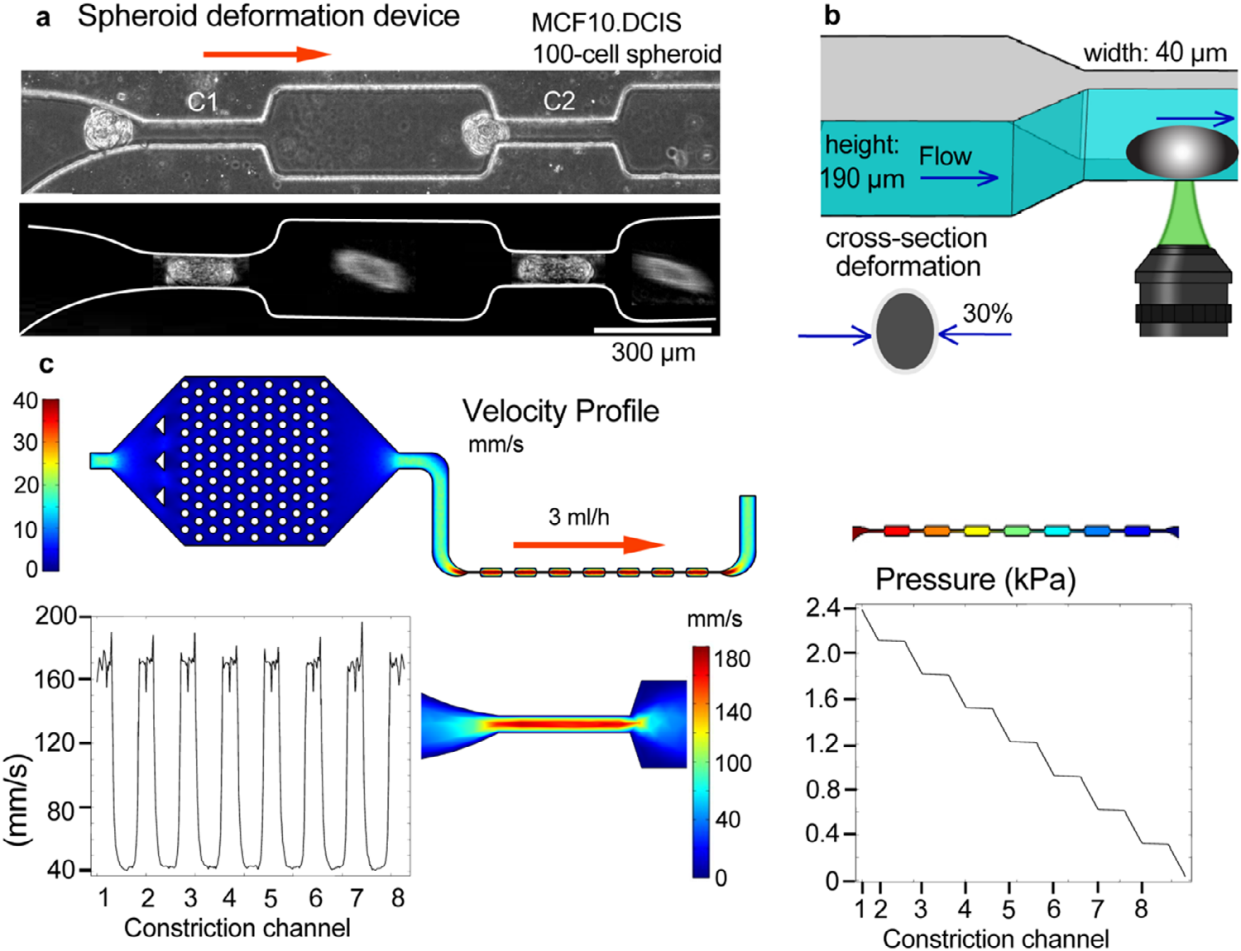
Design and characterization of the deformation device for repetitive deformations of breast tumor spheroids. a) Brightfield image showing a top‐view of MCF10.DCIS.com spheroids entering the first two constrictions (C1, C2) of the deformation device. Constriction channel dimensions are 40 µm width, 190 µm height, and 300 µm length. The bottom image is illustrated schematically to show a spheroid in the channel and in the open space. The top image is not edited. b) Schematic of a spheroid deforming through narrow channels, highlighting compression and elongation due to shear stress, pressure and frictional forces. The average deformation of the cross‐sectional area of spheroids is 30% (Figure S2, Supporting Information). c) 3D Comsol of velocity and pressure profiles within the constriction channels. The modeling was performed considering a laminar flow regime with DMEM media properties. The velocity and pressure profiles are shown in the center of the respective channels. This device was used to evaluate spheroid transit dynamics and deformability under repetitive passage.

Tumor cells can experience deformation as they navigate through different anatomical structures during their metastatic journey.^[^ ^41^, ^42^, ^43^, ^44^, ^45^ ^]^ These deformations expose tumor cell collectives to shear stress, pressure gradients and friction.^[^ ^46^ ^]^ The deformation device was designed to induce controlled and cyclical deformations of tumoral spheroids and consists of a pattern of narrow and wide channels (40 and 200 µm width, respectively) that are periodically repeated (**Figure** **1**a–c). The repeated deformations are designed to mimic loading and unloading events encountered by tumor clusters during dissemination.^[^ ^47^ ^]^ These types of mechanical stress could occur during successive capillary‐sized bottlenecks in the microvasculature and narrow interstitial tracks where clusters undergo serial, rapid deformations separated by brief recovery intervals.^[^ ^45^ ^]^ We incorporated eight constrictions and a flow of 3 mL h^−1^. This configuration was chosen to create a pressure gradient across channels 1–8 (C1 – C8) within a range that mimics solid stress in vivo conditions (≈1–7 kPa).^[^ ^48^, ^49^ ^]^ This design configuration mimics the extent and frequency of the forces experienced by tumor cell collectives in vivo. MCF10.DCIS.com spheroids were used as a model for in situ human breast ductal carcinoma, which are precursors of invasive breast cancer that typically grow within the confinement of the mammary duct.^[^ ^40^ ^]^ Spheroids were generated in low attachment microwells and circulated through the fluidic system (Figure S1, Supporting Information). By subjecting the spheroids to multiple deformations within the device, we aimed to assess mechanical homeostasis by measuring alterations in spheroid properties over time. Spheroids flow into the device and pass through 8 narrow constriction channels, by compressing their width and elongating (Figure 1a,b). Micro‐pillars were inserted as an upstream filter to remove any out‐of‐range spheroids and large debris that could block the channel. Confocal z‐stack imaging of the constricted spheroid indicated that the spheroids occlude approximately half of the channel height, and are compressed 30% of their cross‐sectional area, enabling flow through the open half (Figure S2a,b, Supporting Information). A 30% cross‐sectional deformation lies well within the physiological range experienced by tumor cells in vivo. Primary tumors generate bulk tissue strains on the order of a few to tens of percent,^[^ ^49^ ^]^ while capillary transits often impose significant compression.^[^ ^45^ ^]^ Thus, the device‐imposed deformation is physiologically relevant, reproducing both bulk solid‐stress strains in tumors and the more extreme microvascular constrictions experienced during dissemination. 3D Comsol modeling allowed us to assess the velocity through the constriction channels and the stepwise pressure drop along the channel (Figure 1c). Furthermore, we modeled a spheroid in the channel and estimated the velocity around the compressed spheroid in the range of 200–400 mm s^−1^ with a shear stress of 20–30 Pa and maximum applied pressures of 2.6 kPa (Figure S2c, Supporting Information). Spheroids spent the longest time in the first channel, where they jam; and following shape adaptation they moved more rapidly through subsequent channels (Figure S3; Video S1, Supporting Information).

To account for shear stress effects experienced by the spheroid when introduced into the device, a flow control device was implemented in parallel to the deformation device with open channel dimensions of 200 µm width × 190 µm height (Figure S4, Supporting Information). The spheroids in the device were exposed to lower shear stress (< 1 Pa) due to the absence of constrictions. By comparing the behavior and responses of the spheroids in the deformation device with those in the flow control device, we can distinguish the specific effects attributed to the constrictions, which are the results of induced deformation and locally high shear stress. Using the flow device, we can study the impact of low shear stress on flowing spheroids.

### 2.2 Evaluating the Sensitivity of the Deformation Device and BM Following Actomyosin Contractility Modulation

**Figure 2:**
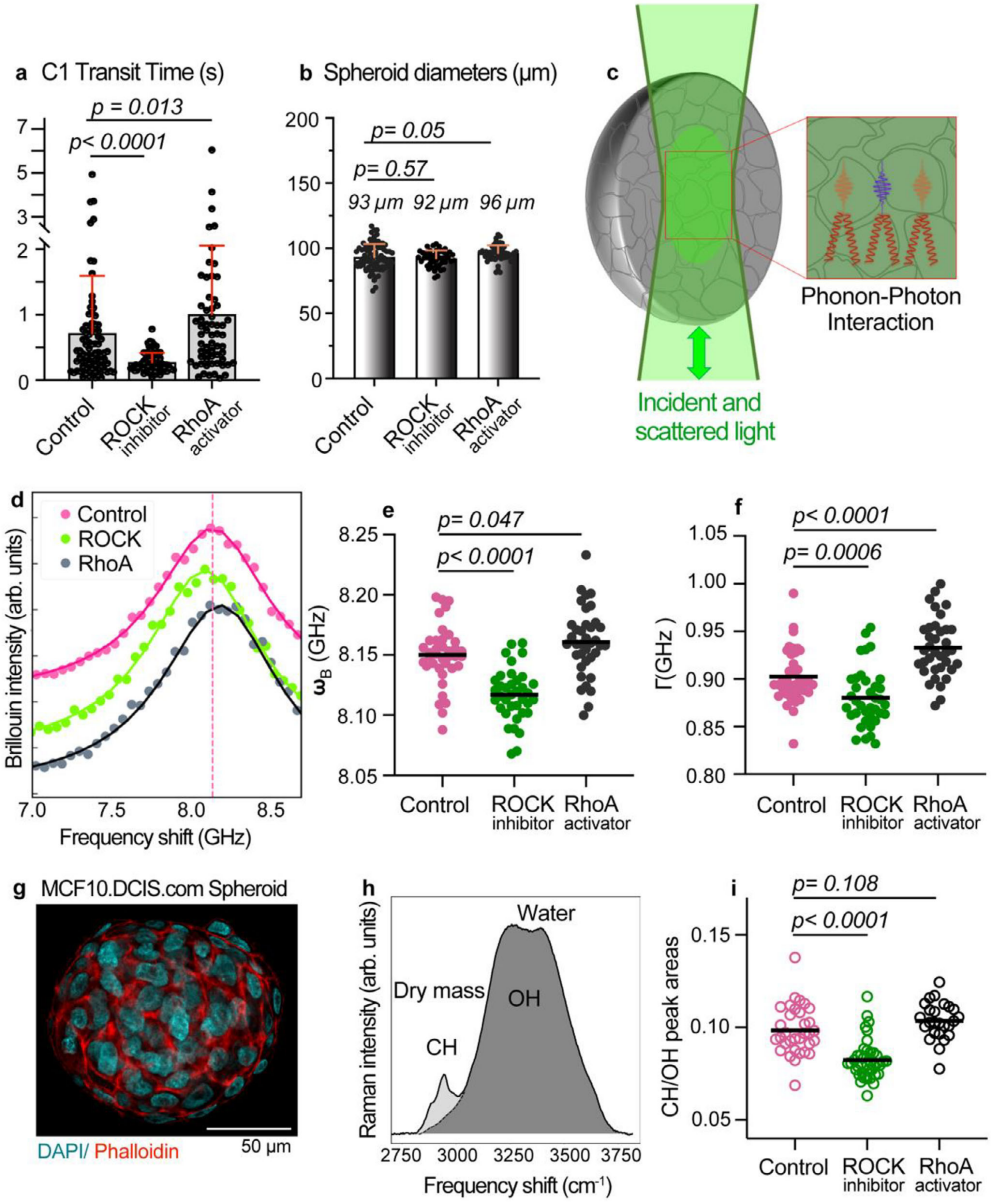
The deformation device transit times correlate with Brillouin frequency shift in MCF10.DCIS.com spheroids treated with ROCK inhibitor and RhoA activator. The spheroids analyzed in 2a‐b were passed through the deformation device, while the spheroids analyzed for 2d‐i were performed in static conditions in a glass bottom dish. a) Transit times of spheroids through channel 1 (C1). Cells in spheroids were treated with 100 nM ROCK 1/2 inhibitors or 10 µg mL^−1^ RhoA activator (CN03) for 24 h, and then introduced into the deformation device at 3 mL h^−1^. Transit times through channel 1 (C1) were obtained from timelapse imaging with a time step of 6 ms. N = 86 control, 76 ROCK inhibitor and 64 RhoA activator‐ treated spheroids. Mean values are shown + SD, each dot represents a spheroid. b) Spheroid diameters are shown before introduction into the devices. N = 82 control, 48 ROCK inhibitor, and 52 RhoA activator‐ treated spheroids. Mean values are shown + SD, each dot represents a spheroid. c) Schematic of the experimental configuration for the Brillouin experiment. The light is focused on the center of the spheroid. Using a 20x magnification objective, the Brillouin signal is averaged over the cells and interstitial water inside the scattering volume. d) Representative Brillouin peak acquired on three different spheroids: control, ROCK 1/2 inhibitors and RhoA activator under static conditions. Dashed vertical line indicates the frequency of the control spectrum. The intensity is arbitrarily shifted for visualization purposes. The solid line represents the DHO fitting function. e,f) Scatter plots of frequency shift, peak width of spheroids. Mean values are shown. Each dot represents a spheroid (N = 30 control, 25 ROCK inhibitor, and 30 RhoA activator‐ treated spheroids). g) Confocal image of a representative MCF10.DCIS.com control spheroid immunostained with DAPI and Phalloidin‐TRITC. h) Representative Raman spectrum acquired on a spheroid. The OH and CH band areas are reported in grey and light grey. i, Scatter plot showing the values of the ratio between the CH and OH areas in the Raman spectra for each spheroid under static conditions. Mean values are shown. Each dot represents a spheroid (N = 30 control, 25 ROCK inhibitor and 30 RhoA activator‐ treated spheroids). *p < 0.05* is considered significant.

To evaluate how effectively Brillouin spectroscopy and the deformation device detect changes in the rheological properties of cell collectives, we applied controlled biomechanical perturbations to the spheroids. This validation strategy involved perturbing actomyosin contractility in MCF10.DCIS.com spheroids using either ROCK inhibitors (which primarily disrupts actin contractility) or a RhoA activator (which enhances actomyosin contractility).^[^ ^50^ ^]^ Actomyosin contractility and actin polymerization play central roles in determining single cell deformability and cortical tension. These forces also influence cadherin‐mediated cell–cell adhesion, modulating adhesion energy, junctional stability, and overall tissue cohesion. In spheroids, these molecular and cellular effects collectively shape the bulk mechanical properties of the aggregate.^[^ ^50^, ^51^, ^52^ ^]^ The experiments were divided into two parts: the first analyzed the dynamic passage of spheroids through microchannels (**Figure** **2**a,b), and the second measured Brillouin and Raman signals from static no‐flow spheroids (Figure 2d–i). This combined approach allowed us to distinguish between flow‐dependent deformation behaviors and intrinsic mechanical properties. In the deformation device, spheroids treated with ROCK inhibitors exhibited significantly faster transit through channel 1, whereas those treated with the RhoA activator showed slower transit compared to ROCK‐inhibited samples (Figure 2a; Videos S2 and S3, Supporting Information). Statistical analysis showed no spheroid size‐dependency on transit time within each group or between groups (Figure 2b; Figure S5; Table S3, Supporting Information). Instead, the altered transit times reflect differences in overall deformability. The extent of deformation experienced by a spheroid during passage through the constricted microfluidic channel is governed by the aggregate's collective mechanical properties. These include viscoelastic bulk behavior, supracellular tension transmitted through cell–cell junctions, which contributes to spheroid cohesion, and the intrinsic resistance of both cells and the intercellular extracellular matrix (ECM) to deformation. Together, these elements define the specific transit time through the microchannel.

To begin dissecting the contribution of these components, we employed Brillouin microscopy (Figure 2c). Brillouin measurements revealed that, relative to controls, spheroids treated with ROCK inhibitors exhibited lower frequency shifts and reduced peak widths, whereas treatment with a RhoA activator produced the opposite trend (Figure 2d–f). Interpreting the Brillouin frequency shift (ω_*B*_) as a proxy for mechanical properties, these findings are consistent with the microfluidic results, confirming that ROCK inhibition leads to global softening of the spheroid structure, while RhoA activation results in a modest increase in stiffness.

To better interpret the Brillouin microscopy results, it is important to consider the internal structure of the spheroids. These structures consist of tightly packed, interconnected cells that occupy ≈85% of the total volume (cell volume fraction = Φ_*cell*_ =  85%) while the remaining space is filled by the ECM (ECM volume fraction =Φ_*ECM*_  =  15%) with characteristic dimensions in the micrometer range (Figure 2g; Figure S6, Supporting Information). The ECM is a highly hydrated material composed primarily of water, with the proteins secreted by the cells accounting for only a small fraction of its total volume.^[^ ^53^, ^54^ ^]^ Under our experimental conditions—specifically using a 20× objective—the Brillouin scattering signal arises from acoustic waves propagating both within single cells and through the ECM. However, given the small difference in the Brillouin frequency shift of the two components (cells and ECM), it is not possible to distinguish them as separate peaks in the spectrum. Consequently, in each measurement point, the observed Brillouin peak represents a composite signal arising from both the ECM matrix and the individual cells within the scattering volume (Figure 2c).^[^ ^29^ ^]^ Considering the comparable scattering efficiency of the two components, we adopted a linear approximation to describe the position of the resulting Brillouin peak ω_*B*_

(1) $$\omega_{B}=\omega_{ECM}\Phi_{ECM}+\omega_{Cell}\Phi_{cell}$$

where ω_*ECM*_ and ω_*Cell*_ are the frequency shifts directly related to the ECM and to the single cell mechanical properties, respectively. This equation was derived considering a first‐ order linearization due to the close proximity of the two underlying peaks, which merge into a single feature in the measured spectrum. Under this condition, the frequency position of the resulting effective peak, ω_*B*_, provides a reliable estimate of the combined contributions of the ECM and cell components. This approximation, which accounts for their respective frequency positions and linewidths yields an accuracy comparable with the experimental uncertainty. Equation (1) indicates that changes in the measured Brillouin frequency shift (ω_*B*_) can result from alterations in the mechanical properties of the ECM, of the individual cells, or from modifications in their relative volume contributions within the probed region.

Therefore, to extract the mechanical properties of individual components from the measured Brillouin shift (ω_*B*_), a quantitative assessment of the scattering volume composition is essential. To this end, we exploited the unique ability of our setup to simultaneously acquire Brillouin and Raman spectra. Raman peak intensities reflect the relative abundance of specific molecular components, since the Raman signal is proportional to the number of scattering molecules within the probed volume and the scattering efficiency of the selected vibrational mode. In particular, the high‐frequency region of the Raman spectrum offers insight into the quantification of the relative water and organic content within the scattering volume (Figure 2h, Experimental Section).^[^ ^55^ ^]^

Specifically:

- The OH stretching band (≈3300 cm^−1^) serves as a proxy for water content
- The CH_2_/CH_3_ stretching bands (≈2900 cm^−1^) reflect the dry mass of the sample, corresponding to vibrational modes of organic constituents^[^ ^56^ ^]^

We modeled the spheroids as a biphasic system in which:

- Cells contain water and dry mass in a fixed ratio (≈70% and 30%, respectively)^[^ ^57^ ^]^
- The ECM is assumed to be predominantly aqueous, with negligible dry mass^[^ ^53^ ^]^

Thus, the relative contributions of cells and ECM to the Brillouin signal can be estimated from the Raman intensity ratio of OH to CH_2_/CH_3_ bands, following the procedure detailed in the Methods and more extensively in ref. [25].

Using this approach, we found that the Raman intensity ratio remained comparable between RhoA‐activated and control spheroids, indicating no significant change in the relative volume fractions of ECM and cells (Figure 2i). Therefore, the differences observed in Brillouin frequency shift and linewidth—particularly the broader linewidth in RhoA‐treated samples—reflect genuine mechanical changes, rather than alterations in composition. As suggested by the observed broadening of the Brillouin linewidth, RhoA activation may lead to increased phonon attenuation, which could reflect enhanced local mechanical heterogeneity.^[^ ^58^ ^]^ In contrast, a marked variation in the Raman peak area ratio is observed between ROCK‐inhibited and control samples, implying a significant increase in water content and ECM volume fraction. Quantitatively, Φ_*ECM*_ increases from ≈15% for control spheroids to ≈24% in ROCK‐treated spheroids (for details, see SI Methods). This shift implies that ROCK inhibition increases extracellular space, which in turn modifies the Brillouin signal (ω_*B*_). These changes, however, were not detectable using high‐resolution 3D confocal analysis (Figure S7, Supporting Information). This result indicates that BRmS measurements conducted with low‐magnification objectives exhibit higher sensitivity, enabling direct assessment of mean compositional and mechanical properties in bulk 3D samples.

Overall, we show that microfluidic transit times provide a readout of mechanical properties in breast tumor spheroids, which are consistent with the frequency shifts of the Brillouin peaks. Given that spheroids are composite materials, their global mechanical behavior arises from the combined contributions of individual cells and the ECM, as well as their respective volume fractions. By integrating Raman spectroscopy, we proposed a method to estimate the compositional influence—specifically the cell‐to‐ECM ratio—on the Brillouin signal, thereby enabling a more accurate interpretation of the data.

### 2.3 BRmS On‐a‐Chip: Spheroids under Compression

**Figure 3:**
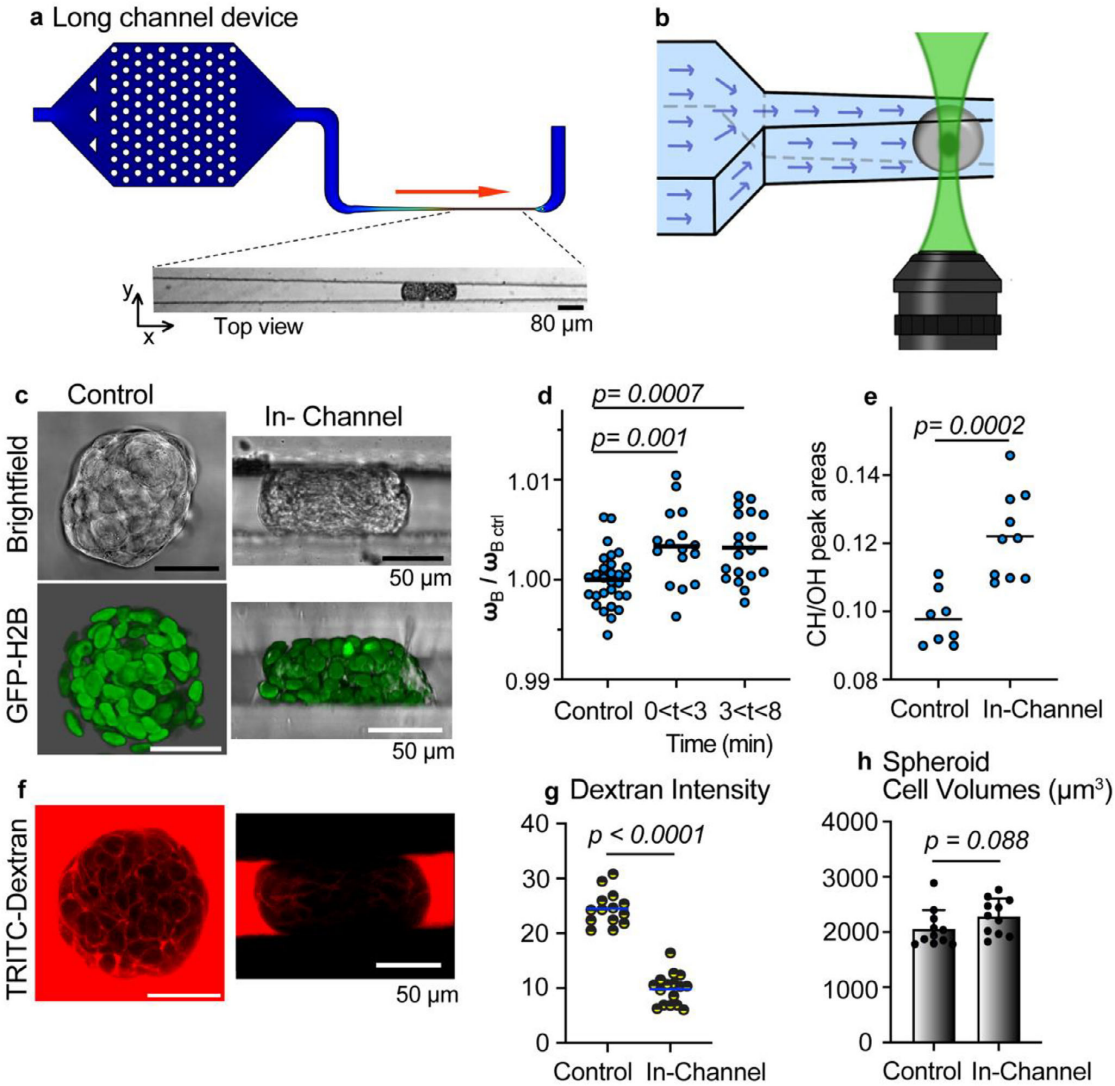
Sustained compression of spheroids in micro‐channels leads to stiffening. a) Comsol velocity profile of the long‐channel device, in which spheroids are gradually compressed from a 200 to 40 µm wide channel. The spheroids are introduced into the channel at 1 mL h^−1^ and then the flow is stopped to hold the spheroid stationary under compression (bottom inset). The image depicts two spheroids in the channel. b) Schematic of the experimental configuration for the Brillouin, Raman and fluorescence experiments. c) Representative images of control and compressed (In‐Channel) spheroids. The top image shows the spheroids using Brightfield, and the bottom shows 3D confocal rendering. Green represents fluorescent GFP‐H2B nuclei. d) Brillouin frequency shift of control and compressed spheroids. The time indicates the compression time. Mean values are shown. Each dot represents a spheroid, imaged in the center using a 20x objective. N = 29 control spheroids, 16 spheroids for 0 < t < 3 min and 19 spheroids for 3 < t < 8 min. e, Results for the ratio of the CH and OH Raman areas for control and compressed spheroids. Each dot represents a spheroid. Mean values are shown. N = 8 control and 10 in‐channel spheroids. f) Representative images of a control and compressed spheroid in 2 g L^−1^ TRITC‐3 kDa dextran. g) Average dextran intensities plotted for control and compressed spheroids. The intensity is measured over the spheroid area in the middle z‐plane, and background intensities are subtracted (representing spheroids without dye). The control intensities are obtained after the dye has filled the spheroid. The In‐Channel spheroid intensity is obtained 10 min at which the dye has exited the spheroid, and the intensity has stabilized. N = 14 control and 16 in‐channel spheroids. h) Spheroid cell volumes are plotted for control and compressed conditions. Average cell volumes were determined with confocal imaging and 3D cell re‐construction software (Arivis). Mean values + SD are shown in the graphs and each dot represents a spheroid (n = 11 for each condition). *p < 0.05* are considered significant.

To validate the BRmS‐on‐a‐chip approach, we investigated the mechanical response of breast tumor spheroids subjected to confined compression using a custom‐designed long‐channel microfluidic device (**Figure** **3**a,b; Figure S8, Supporting Information). The channel gradually narrows from 200 µm to 40 µm, creating a compression zone. Spheroids were introduced into the device at 1 mL h^−1^ and compression was initiated by halting the flow once the spheroid reached the 40 µm‐wide constriction (Figure 3c). Under sustained compression (up to 8 min), spheroids exhibited a significant increase in the Brillouin frequency shift, which persisted throughout the entire compression period (Figure 3d). Raman spectral analysis simultaneously revealed changes in chemical composition (Figure 3e), consistent with water loss. Given that the interstitial ECM behaves as a poroelastic material—i.e. highly compressible relative to cells,^[^ ^59^ ^]^ it is predicted to bear the majority of the deformation, thereby losing water under pressure.

To further support this hypothesis, we applied orthogonal measurements using fluorescence microscopy. To directly assess fluid displacement in the ECM, we incubated spheroids with 3 kDa TRITC‐dextran, a small fluorescent molecule that rapidly diffuses into the interstitial space but is excluded from cells (Figure 3f). Following equilibration, spheroids were compressed in the microfluidic channel, and real‐time imaging revealed a significant reduction in dextran fluorescence intensity compared to uncompressed controls (Figure 3g; Figure S9, Supporting Information). Quantitative analysis confirmed a clear loss of interstitial signal under compression, supporting the notion that compression expels interstitial fluid and confirming the ECM as the primary site of water loss.

To exclude intracellular volume changes as a contributing factor, we performed 3D volumetric reconstructions using confocal microscopy combined with image segmentation tools. This enabled quantitative estimation of individual cell volumes within the spheroids under compression. Although compressed cells show evident morphological alterations, no significant changes in single‐cell volume were detected between compressed and control conditions (Figure 3h). This indicates that the bulk deformation and associated mechanical signals arise from ECM volume reduction, rather than from intracellular volume loss.

Under this condition and taking into account the reduction of water content estimated by the Raman data, we found that, within the experimental error, the very small interstitial spaces under compression become negligible for Brillouin measurements (Φ_*ECM*_ ≈ 0). Therefore, the Brillouin frequency shift ω_*B*_ measured for spheroids in channel coincides with ω_*CELL*_ (Equation 1). Comparing the Brillouin shift values of spheroid before and after compression, it is also possible to estimate ω_*ECM*_ for the uncompressed spheroid which is found to be ≈8 GHz, well in the range of the expected values for a highly hydrated ECM.^[^ ^60^ ^]^

### 2.4 BRmS On‐a‐Chip: Shape Recovery after Cyclic Compression

**Figure 4:**
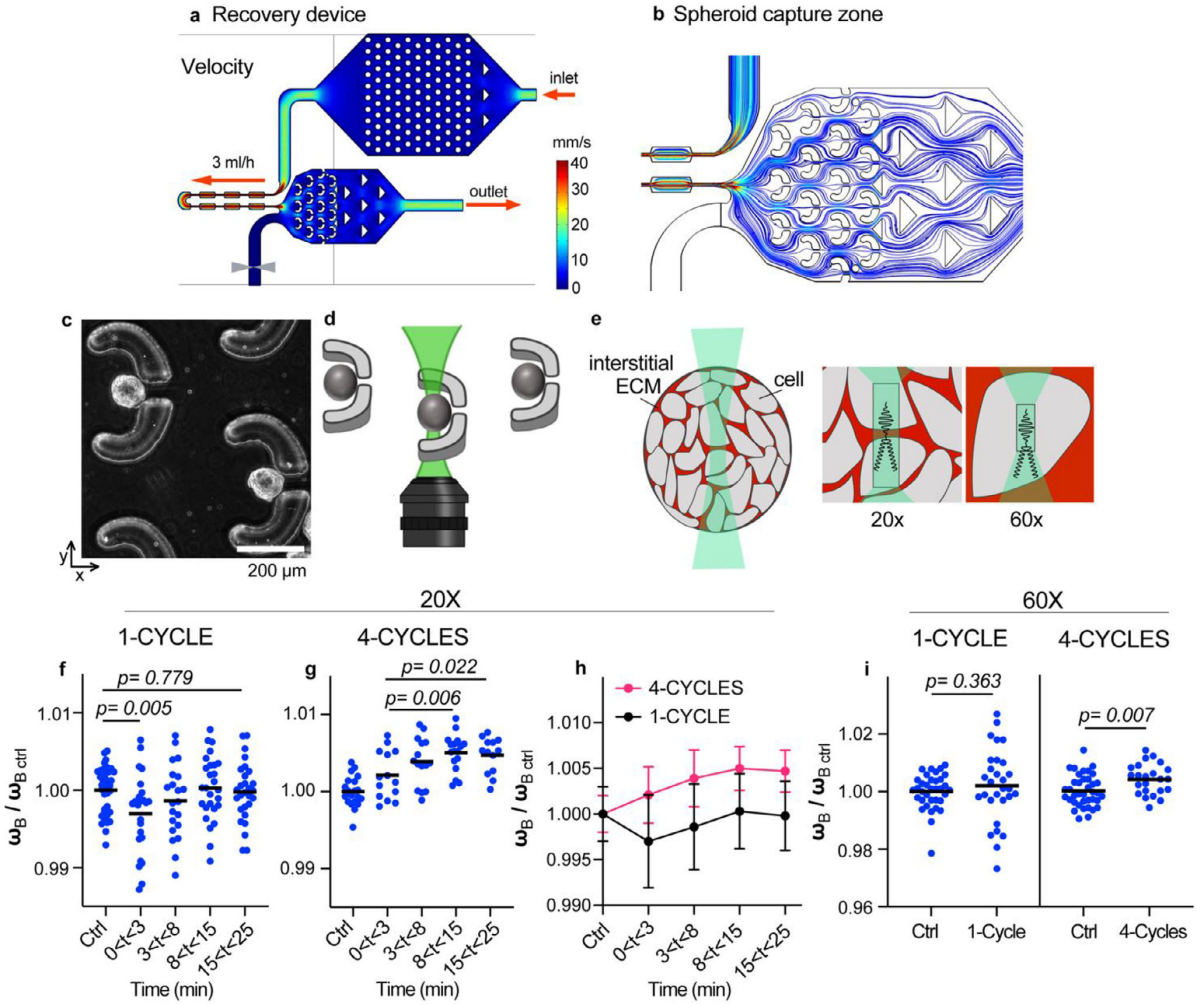
MCF10.DCIS.com spheroids recover after 1 cycle and mechanically stiffen following 4 cycles of deformation events. a,b) Comsol velocity profiles and streamline simulations of the recovery device, which incorporates downstream capture zones. c) Brightfield picture and schematic image of spheroids capture post‐deformation in recovery traps. Spheroids were introduced in cell culture media at a constant flow rate of 3 mL h^−1^. The flow rate was stopped after the spheroids enter the capture sites. d) Schematic of Brillouin and Raman set up under the recovery traps. e) Schematic showing the scattering volume in a spheroid with 20x (cells and ECM) versus 60x objective (cells only). f–i): Ctrl represents control spheroids that did not pass through the device. f) Brillouin frequency shifts plotted for the global spheroid (cells & ECM) after 1‐cycle, the dots are showing individual spheroids. Mean values are shown. N = 37 control spheroids, 24 spheroids for 0 < t < 3 min, 21 spheroids for 3 < t < 8 min, 27 spheroids for 8 < t < 15 min and 28 spheroids for 15 < t < 25 min post‐ deformations. g) Brillouin frequency shifts plotted after 4‐cycles, showing individual spheroids. Mean values are shown. N = 20 control spheroids, 14 spheroids for 0 < t < 3 min, 15 spheroids for 3 < t < 8 min, 17 spheroids for 8 < t < 15 min and 13 spheroids for 15 < t < 25 min. Frequency shifts were normalized to controls. All data was acquired in the center of the spheroid using a 20x objective (focus spot size ≈ 2 × 2 × 23 µm). h) Brillouin frequency shifts of the spheroid accounting for cells & ECM contributions. The data after 1‐cycle versus 4‐cycles (normalized to controls) are reported over time post‐ deformations. Mean ± SD are plotted. The number of spheroids per condition are recorded in f,g legends. i) Brillouin frequency shifts of single cells within spheroids after 1‐cycle versus 4‐cycles, respectively, the dots are showing individual spheroids. The timescale for the analysis was 10 < t < 30 min post‐deformation. N = 35 control spheroids and 29 1‐cycle spheroids, and N = 38 control spheroids and 23 4‐cycle spheroids. Frequency shifts were normalized to controls. All data was acquired in the center of the spheroid using a 60x objective (focus spot size ≈0.5 × 0.5 × 4 µm). Mean values are shown. *p < 0.05* are considered significant.

To investigate the dynamics of shape recovery in spheroids following deformation, we introduced a modification to the microfluidic deformation device, in a version called the recovery device. This design included a downstream capture zone positioned after the constriction channels (**Figure** **4**a,b; Video S4). The pressure and velocity profiles across the constriction channels were preserved to maintain consistent mechanical stress conditions. The added capture zone consists of an array of traps with a central 40 µm gap, creating a nozzle‐like effect that facilitates spheroid retention. Within this region, the flow rate and pressure are substantially reduced, minimizing further deformation (Figure S10, Supporting Information). Additionally, a flush line equipped with a valve enabled efficient release of captured spheroids, allowing repeated use of the capture zone.

After the deformed spheroids were captured in the traps, Brillouin and Raman microscopy were conducted to track the spheroids’ properties over time (Figure 4c,d). Spheroids were cycled through the constriction channels 1‐cycle (representing 8 constrictions) and 4‐cycles (32 constrictions). Each cycle takes on average 0.8 s (device passage time) and 10 min recirculation time. Using timelapse video analysis, we tracked spheroid shapes in the recovery zone. Initially, the spheroids were elongated as they retained a shape memory from the deformation channel; however, they rapidly recovered their round shape within 10–20 s. This shape recovery behavior was observed independent of the number of cycles (Figure S11; Videos S5 and S6, Supporting Information). Despite this rapid morphological recovery, Brillouin spectroscopy revealed persistent changes in mechanical properties.

Measurements were performed after 1‐cycle versus 4‐cycle of deformations using two microscope objectives which provided different spatial resolutions. The 20× objective captured a scattering volume that includes both the ECM and cells, while the use of 60× objective confined the scattering volume inside single cell enabling direct probing of the single cells mechanics (Figure 4e; Figure S12, Supporting Information, Methods‐ “Multimodal Brillouin microscopy set up”). These two objectives capture two different levels of heterogeneity, as illustrated in the maps (Figure S13, Supporting Information).

#### 2.4.1 1‐Cycle Perturbations

**20× objective**‐ After a single deformation cycle, the Brillouin frequency shift initially decreased, reflecting mechanical softening, without changes in Raman spectra (Figure S14a, Supporting Information). We interpret the transient reduction of Brillouin shifts to represent changes in water distribution or reversible cytoskeletal rearrangements. This effect could stem from physical properties of the ECM or cell‐intrinsic constituents, such as the cytoskeleton, the nucleus, or cytoplasmic density, as previously reported.^[^ ^61^ ^]^ Over time, this reduction was gradually reversed, with near‐complete recovery of the Brillouin signal within 15 min (Figure 4f), indicating the restoration of the global mechanical properties.

**60× objective**‐ Further insight was gained through Brillouin microscopy at single‐cell resolution, focused on the interior of cells within the spheroids. After a single deformation cycle, the Brillouin frequency shift at the cellular level remained comparable to that of control cells (Figure 4i; left graph). These findings support a model in which the rapid onset stress‐induced mechanical of memory and adaptation in spheroids are driven primarily by extracellular, rather than intracellular, contributions.

#### 2.4.2 4‐Cycle Perturbations

**20× objective**‐ In contrast to the transient mechanical response observed after a single deformation cycle, the Brillouin frequency shift after four deformation cycles revealed a significant increase (Figure 4g,h). Raman analysis indicates that the stiffening response is not attributed to changes in water content, as there are no observable changes between t = 0 and t = 25 min post‐deformation (Figure S14b, Supporting Information). Thus, it may instead reflect a mechanically adaptive response of the spheroid to repeated mechanical stress, possibly reflecting both cellular adaptation and alterations in the surrounding matrix. Importantly, Brillouin microscopy analysis of spheroids subjected to only shear stress due to the fluid flow (i.e., passaging through non‐constricted channels) did not show any significant variation as compared to controls (Figure S15, Supporting Information).

**60× objective**‐ Data acquired after four deformation cycles revealed significant mechanical differences at the single‐cell level (Figure 4i; right graph). This finding provides evidence that repeated cyclic deformations lead to cumulative mechanical changes, involving cellular adaptation.

The 20× Brillouin data from Figure 4f,g was combined in Figure 4h, to provide a visual comparison between the two different deformation cycles in the same plot. Interestingly, the temporal evolution of the Brillouin frequency shift follows a similar trajectory after both one and four deformation cycles (Figure 4h), with a gradual stabilization over 15 min. This temporal profile indicates a shared recovery process—potentially involving strain‐induced ECM modification. However, the persistent offset between the two curves points to enduring differences in the mechanical state, which are likely attributable to single‐cell‐level alterations resulting from repeated stress exposure.

Brillouin measurements were further supported by the evolution of transit times through the microfluidic constrictions (Figure S16a, Supporting Information). We compared the C1 transit times between 1–5 cycles of deformation and noticed that there was a reduction in C1 transit times during the second cycle relative to the first. This may reflect transient softening, consistent with Brillouin measurements immediately following 1‐cycle deformation (Figure 4f). Subsequent deformation cycles led to a gradual recovery of transit times to control levels, consistent with restoration of mechanical integrity within the spheroid architecture. Yet, after four cycles, transit times increased across all constrictions (C1‐C3), indicating a progressive stiffening response (Figure S16a,b, Supporting Information). This was paralleled by a sustained increase of the Brillouin frequency shift observed 15 min after 4‐cycles of deformation (Figure 4g), reinforcing the hypothesis of adaptation to mechanical load.

Overall, these results support the view that tumor spheroids exhibit mechanical memory and adaptive stiffening in response to repeated deformation. We found that early mechanical softening primarily reflects ECM changes, while later stiffening involves a combination of cellular and extracellular remodeling. These findings prompt further investigation into whether the history of mechanical stress also impacts transcriptional reprogramming and long‐term phenotypic adaptation.

### 2.5 Spheroids Respond to Deformations Through Alterations in Nuclear Shape, Gene Expression, and Invasive Capabilities

Force‐induced changes in structural arrangement of the chromatin play a critical role in regulating gene expression programs.^[^ ^62^ ^]^ To ensure that the observed mechanical adaptations were not confounded by cellular damage, we assessed membrane integrity and viability following repeated mechanical deformation. After subjecting MCF10.DCIS.com spheroids to four deformation cycles, we performed a cell membrane integrity assay using a live‐cell–permeable aqua dye (Figure S17, Supporting Information). This dye selectively enters cells only when the plasma membrane is compromised. Our results demonstrated that membrane integrity was preserved, indicating that repeated mechanical cycling did not induce overt membrane damage. Using a live cell cytoplasmic dye, we also showed that cells were viable (Figure S18, Supporting Information), whereas analysis of the proliferation marker Ki67 (Figure S19, Supporting Information) indicated that the proliferation rate was unperturbed by the deformation. Together, these results demonstrate that repeated mechanical deformation preserves key cellular functions, including membrane integrity, viability, and proliferation.

To investigate intracellular responses to mechanical stress, we quantitatively examined nuclear morphology within spheroids using high‐resolution confocal imaging combined with an automated FIJI macro for image analysis. Cells subjected to mechanical forces often undergo shape remodeling not only at the plasma membrane and actin cortex but also at the level of intracellular organelles, including the nucleus, centrosome, Golgi apparatus, endo‐lysosomes, and mitochondria.^[^ ^3^ ^]^ The extent and dynamics of these deformations are governed by both cell‐intrinsic properties and the physical characteristics of the surrounding environment.^[^ ^4^ ^]^

**Figure 5:**
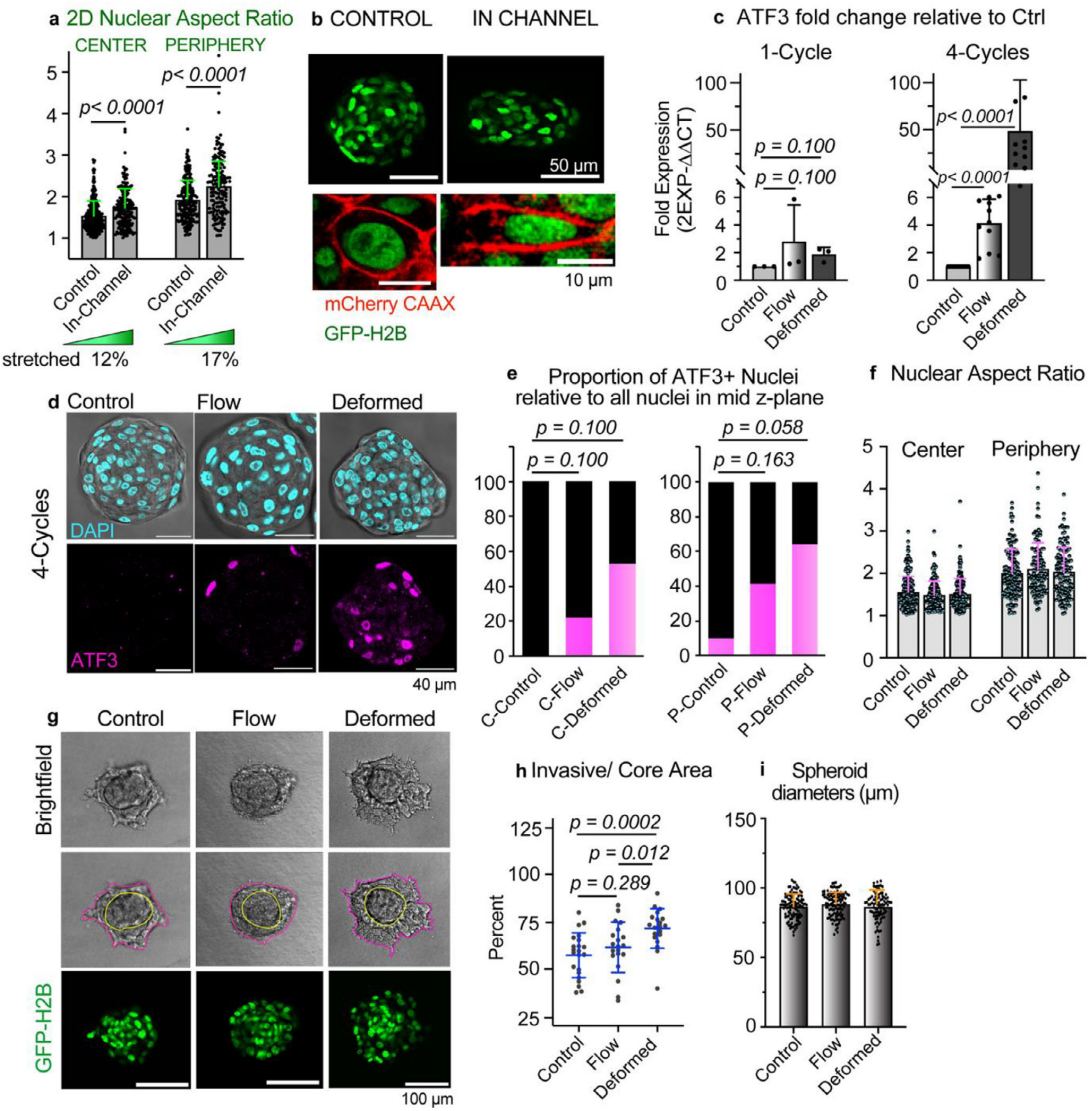
Repetitive spheroid deformations increase ATF3 levels and enhance collagen invasion. a) 2D nuclear aspect ratios were extracted from the central z‐plane of spheroids that were compressed in the channel. Central versus peripheral nuclei were plotted. Aspect ratios were determined through an automated Fiji macro. Dots represent spheroid nuclei. # of spheroids = 10 control and 19 in‐channel. # of nuclei analyzed = 297 versus 184 (control center vs periphery) and 180 versus 159 (in‐channel center vs periphery). Mean values + SD are shown. b) Representative 63X confocal images of control and compressed spheroids. Nuclei are labeled with GFP‐H2B and cell membranes are labeled with mCherry‐ CAAX. c–i) Spheroids were cycled through the deformation device and the flow device, respectively. Control spheroids did not pass through a device. c) Quantitative PCR results showing relative ATF3 gene expression of spheroids cycled through the respective device (1 cycle vs 4 cycles) and cultured for 1 h post‐chip. Values were normalized to the controls. Dots represent experiment repeats. Mean + SD are shown. *p < 0.05* are considered significant. N = 1500–3000 spheroids per condition. d) Confocal imaging with 63X objective of spheroids cycled four times through the devices and cultured for 4 h post‐chip. Spheroids were immunostained with DAPI and ATF3 conjugated to an anti‐rabbit Alexa fluor 647 antibody. Central z‐plane of spheroids are shown. e) Proportion of ATF3‐positive nuclei relative to all nuclei in the spheroid central z‐plane. ATF3‐positive nuclei are identified by applying a threshold intensity value (see Methods) and represented by the pink portion of the graph. Intensities are normalized to secondary antibody controls. C‐ references the spheroid center, and P‐ represents the periphery. Mean intensity values are plotted. N = 10 control spheroids (218 center vs 126 peripheral nuclei), 8 flow spheroids (146 center vs 104 peripheral nuclei) and 9 deformed spheroids (156 center vs 114 peripheral nuclei). f) Nuclear aspect ratios are quantified using the DAPI channel for the center versus peripheral nuclei. Dots represent spheroid nuclei. Mean values are shown + SD. The number of spheroids and nuclei analyzed are the same as in (e). g) Spheroids were cycled four times through the devices and were immediately placed into the collagen matrix. Invasion was monitored for 24 h. Confocal imaging with a 20x objective was performed of the invading spheroids after 24 h of culture in 3 mg mL^−1^ collagen‐I. Yellow tracing indicates the compact spheroid core, while the pink outlet represents the invasive area of the spheroid. h) Invasive/ core areas are plotted for control, flow spheroids and deformed spheroids (N = 22 control, 20 flow and 20 deformed spheroids). Mean ± SD are shown. *p < 0.05* are considered significant. i) Spheroid diameters are shown pre‐chip. The mean diameters for control, flow and deformed, respectively are 86, 88, 86 µm. Dots represent individual spheroids (N = 107 control, 111 flow and 89 deformed spheroids). Mean ± SD are shown. *p < 0.05* are considered significant.

To enable precise morphological tracking, we generated MCF10.DCIS.com cells stably expressing fluorescent markers: H2B‐GFP to label nuclei and CAAX‐mCherry to delineate the plasma membrane. Using this dual reporter system, we analyzed nuclear shape across entire spheroids, distinguishing between cells located in the peripheral and central regions. Spheroids subjected to mechanical deformation exhibited significantly more elongated nuclei, as quantified by increased nuclear aspect ratios, compared to untreated controls. This effect was consistently observed in both peripheral and core cells. The elongation was evident in both 2D projections and full 3D reconstructions, confirming a robust deformation signature at the nuclear level (**Figure** **5**a,b; Figures S20–S22; Videos S7 and S8, Supporting Information).

Transcriptional changes have been previously linked to nuclear deformation and mechanical stress in individual cells.^[^ ^42^, ^62^, ^63^, ^64^ ^]^ Hence, we performed bulk RNA sequencing on MCF10.DCIS.com spheroids subjected to four deformation cycles, followed by 1 h of recovery in culture, to capture early gene expression responses. Control spheroids (not introduced into any device) and spheroids that went through the flow control device were included in the experiment. As expected, the number of early responding differentially expressed transcripts was limited. Nevertheless, we observed a very significant and robust upregulation of the ATF3 (activating transcription factor 3, Figure S23, Supporting Information). In addition to ATF3, there was an increase in early response transcriptional co‐activators FOSB, JUN, and NR4A3, as well as an upregulation of inflammatory response genes IL6, CXCL2, EGR1, and EGR4 (Figures S23 and S24, Supporting Information). ATF3 is a stress‐inducible gene and is transcriptionally activated in a rapid and transient fashion by various types of stress.^[^ ^65^, ^66^, ^67^ ^]^ It is an early response transcription factor that acts as a master regulator in metabolism, immunity, and oncogenesis. Using quantitative PCR analysis, we validated the rapid and robust upregulation of ATF3 transcript levels that increased 48‐fold in spheroids cycled four times through the device, but not after a single cycle (Figure 5c). We also observed a four‐fold increase in spheroids that passed through the flow recovery device. These data are consistent with reports indicating that shear stress can increase ATF3 transcript levels.^[^ ^68^ ^]^ These results suggest that while shear stress alone is sufficient to upregulate ATF3, its expression is further potentiated by compressive mechanical stress.

The transcriptional response was corroborated at the protein level: ATF3 protein expression was significantly elevated in deformed spheroids, as shown by both immunofluorescence staining and western blot analysis (Figure 5d,e; Figure S25a, Supporting Information). Notably, immunostaining revealed that peripheral cells within the spheroid exhibited the strongest ATF3 signal, indicating that these cells experienced higher levels of mechanical stress during passage through the constrictions. This regional difference paralleled morphological changes, as peripheral nuclei were more elongated than those in the spheroid core (Figure 5f).

ATF3 is shown to have also a role in promoting breast cancer cell motility;^[^ ^69^ ^]^ thus, we next evaluated whether repeated mechanical deformation would affect the invasive behavior of MCF10.DCIS.com spheroids. Spheroids were embedded in a 3 mg mL^−1^ collagen‐I matrix, which is a physiologically relevant extracellular matrix characterized by native pore size and stiffness heterogeneity and is suitable for modeling invasive potential in vitro. Confocal imaging of spheroids 24 h after deformation revealed a clear distinction between the central core (yellow) and a peripheral invasive zone (pink), with deformed spheroids displaying a markedly expanded invasion front compared to non‐deformed controls (Figure 5g; Figure S26, Supporting Information). Quantification of the invasive‐to‐core area ratio confirmed a statistically significant increase in invasiveness following four deformation cycles (Figure 5h), an effect observed even when initial spheroid sizes were controlled for (Figure 5i).

These results suggest that the history of mechanical stress enhances the invasive phenotype of epithelial spheroids, potentially mediated by mechanically induced transcriptional reprogramming, including the upregulation of ATF3.

## 3 Conclusion and Outlook

In this study, we introduce an integrated platform—BRmS‐on‐a‐chip—that combines Brillouin and Raman spectroscopy with four complementary microfluidic devices. These tools enabled us to systematically monitor spheroid mechanical responses under repetitive constriction, sustained compression and cyclic deformation with recovery, respectively. By tracking shape, composition, and stiffness in real time, we were able to dissect the individual contributions of cells and ECM to the mechanical properties of 3D tumor collectives. Raman and Brillouin measurements allowed us to decouple mechanical changes from volumetric alterations in ECM and cellular fractions. Under compression, cells behaved as incompressible units, maintaining volume despite shape deformation, while the ECM exhibited poroelasticity, losing water and contributing to spheroid compaction.^[^ ^59^ ^]^

Reversible mechanical changes—captured using the deformation and recovery devices—demonstrated dynamic mechano‐adaptive behavior. Specifically, transient softening was observed after a single constriction cycle, whereas repeated cyclic deformations in the recovery device led to a persistent stiffening response, consistent with mechano‐adaptation. Single‐cell Brillouin analysis confirmed that intrinsic cellular mechanics are altered by repeated stress. These biomechanical adaptations were accompanied by rapid transcriptional changes, including robust upregulation of the stress‐responsive transcription factor ATF3, which is known to modulate motility and inflammation. Functionally, this was associated with enhanced invasiveness in collagen matrices, highlighting a potential mechano‐transcriptional axis driving tumor progression.

The BRmS‐on‐a‐chip platform enables systematic mechano‐phenotyping of 3D collectives and can be extended in several directions. The set up can be applied toward non‐cancerous cell lines to probe whether repeated stress triggers an invasive transition, potentially programming an epithelial‐to‐mesenchymal transition (EMT).^[^ ^69^ ^]^ Notably, ATF3 levels were increased following 4‐cycles of deformation in the normal HaCaT keratinocyte spheroids and mammary MCF10A spheroids (Figure S25b,c, Supporting Information). These results indicate that a mechano‐stress response pathway also occurs in non‐malignant cells. Looking forward, the BRmS‐on‐a‐chip platform can be exploited to probe how therapeutic interventions modulate mechanical responses, and whether such adaptations differ according to the aggressiveness of the tumor state.

Methodological considerations for Brillouin microscopy in 3D spheroids are also critical for future directions. Despite the clear advantages of Brillouin spectroscopy, its widespread use is still limited for several reasons. These include the caution required in data analysis and interpretation, as well as the complexity of Brillouin setups, which are often confined to specialized photonics laboratories. While methodological efforts are being made to establish a consensus among researchers in the field,^[^ ^14^ ^]^ at a more fundamental level there are still open questions that remain to be addressed. In dense, biphasic spheroids, Brillouin readouts can be influenced by local water content and mass density.^[^ ^25^ ^]^ Thus, analysis should be paired with Raman measurements to explicitly control for these variables. Additional practical limitations include reduced signal in turbid regions, finite axial sampling volumes, and low acquisition throughput. These can be mitigated by including objectives to analyze global and intracellular mechanics. Improving acquisition speeds and refractive index and density co‐mapping could be applied toward future directions.

In summary, our findings demonstrate that mechanical history influences both the physical state and gene expression programs of tumor spheroids. The BRmS‐on‐a‐chip approach, supported by the complementary deformation, long‐channel, and recovery devices, provides an innovative and powerful framework to interrogate mechano‐adaptation dynamics. By integrating these distinct experimental regimes, we could directly link deformation history to nuclear remodeling, transcriptional reprogramming and enhanced invasive potential.

## 4 Experimental Section

### Cell Culture

MCF10.DCIS.com cells were cultured in DMEM/F12 media (Invitrogen) supplemented with 5% horse serum (Invitrogen), 2 mM L‐glutamine (Euroclone), 1% penicillin‐streptomycin (Sigma), 0.5 µg mL^−1^ hydrocortisone, 10 µg mL^−1^ insulin and 20 ng mL^−1^ human epithelial growth factor (EGF, Invitrogen). Cells were grown in cell culture dishes 100×20 mm (CELL STAR) at 37 °C in humidified atmosphere with 5% CO_2_. Spheroids were created in low attachment Aggrewell dishes (StemCell). Spheroids were treated with 100 nM ROCK 1/2 inhibitor (“Compound 3”, previously characterized pharmacologically by Chiesi Farmaceutici.^[^ ^70^ ^]^ The inhibitor was provided under a Material Transfer Agreement. Chiesi; CH 051672) or 10 µg mL^−1^ RhoA activator (CN03: Cytoskeleton Inc.) at the time of cell seeding in Aggrewell plates and cultured for 24 h.

### Transduction

MCF10.DCIS.com were infected with a pSLIK‐neo‐EV construct (empty vector, control) and selected with neomycin to obtain a stable population. Constitutive expression of GFP H2B was achieved by retroviral infection of MCF10.DCIS.com cells with pBABE puro‐GFP‐H2B vector (provided by the IFOM Cell Culture Facility). MCF10.DCIS.com EV GFP‐H2B cells were further transduced with mCherry‐CAAX construct (Addgene, plasmid #129285) following the lentiviral transfection protocol. Cells were sorted for high GFP and high mCherry signal using FACS sorter (MoFlo Astrios). Constitutive expression of mCherry H2B was achieved by retroviral infection of MCF10.DCIS.com cells with pBABE puro‐mCherry‐H2B vector (also provided by the IFOM Cell Culture Facility).

### Microfluidic Experiments

Briefly, MCF10.DCIS.com cells were seeded in low adhesion Aggrewell dishes (StemCell) to obtain ≈1200 spheroids composed of 100 cells each. Spheroids grew for 24 h, after which they were collected and processed through the microfluidic device at a constant flow rate.

### Deformation Device

Spheroids were cycled through the same fluidic channel 1–5 times at 3 mL h^−1^ to mimic high frequency deformation events. Each channel has 8 constrictions (40 µm wide, 190 µm high, and 300 µm length). Each passage lasted 0.8 s; and the re‐circulation time was 10 min between cycles. After the deformation was performed, spheroids were collected and cultured in 6‐well low attachment dishes (Corning) for the desired time point.

### Flow Control Device

The flow control device has the same design as the deformation device but without constrictions (200 µm wide, 190 µm height). Spheroids were cycled through the same fluidic channel 1–4 times at 3 mL h^−1^ to mimic the effects of laminar shear stress. After the experiment was performed, they were collected and cultured in 6‐well low attachment dishes for the desired time point.

### Long‐Channel Device

Spheroids were introduced into the microfluidic device at 1 mL h^−1^. The channel gradually narrows from 200 to 40 µm. Spheroids stopped in the narrow part of the channel where they were imaged in the absence of flow.

### Recovery Device

Spheroids were introduced into the recovery device at 3 mL h^−1^ and captured in the downstream traps. The recovery device has the same design of the constriction channels as the deformation device. After the spheroids were captured, the flow was halted and the spheroids were imaged over time. Detailed experimental conditions are found in the Supplementary Information.

### Multimodal Brillouin Microscopy Set Up

Brillouin and Raman spectra were acquired simultaneously using the same focusing and collecting optics. The integrated Brillouin–Raman microscope (BRmS) couples the same excitation laser and collection optics to a multipass tandem Fabry–Pérot interferometer (TFP‐2 HC) and a Raman spectrometer (Horiba) respectively as deeply discussed in refs. [15, 26]. In brief, a green laser (Spectra Physics Excelsior) with a wavelength of λ = 532 nm is focused on the sample with a power of 10 mW. The backscattered light is collected by the same objective. During the measurements, the spheroids were maintained in the cell media inside an incubator chamber (UNO‐T‐H‐PREMIXED – OKOLAB) at 37 °C in a controlled humidity and CO_2_ conditions (5% CO_2_).

### Low Spatial Resolution Brillouin Analysis

Correlative Brillouin and Raman analysis were performed on the spheroid using 20x long working distance objective (M‐Plan Apo ×20 objective lens from Mitutoyo, Kawasaki, Japan NA = 0.45; axial resolution of 23 µm; lateral resolution of 2 × 2 µm. This resolution is well confined within the spheroids which have a characteristic diameter of ≈100 µm. The measurement location was carefully controlled by focusing on the equatorial plane of the spheroid, thereby minimizing variability arising from peripheral versus core positioning. As Brillouin and Raman microscopy are highly sensitive to the composition of the scattering volume, care was taken to maintain consistent acquisition depth and focus across all samples. This configuration captures the average mechanical and chemical properties of the spheroids probing both cells and ECM.

### High Spatial Resolution Brillouin Analysis

Brillouin analysis was performed also on single cells within the spheroid using a 60x water immersion objective (UPLSAPO 60XW from Olympus NA = 1.2 with an axial resolution 4 µm; lateral resolution of 0.5 × 0.5 µm). The 60x objective, which has a higher numerical aperture, is capable of focusing the light in a smaller area, increasing the axial resolution, to ≈ 4 µm. This configuration allows the laser spot to be focused entirely within a single cell, enabling measurement of the mechanical properties of the single cells within the spheroid. Also in this case, the equatorial plane was selected as focusing plane, while avoiding peripheral regions—where buffer or strain effects might bias the measurement. Moreover, also nucleoli, which are the stiffest subnuclear structures, were avoided to prevent any modification introduced by their presence. Their location is easily detectable in the brightfield images. The Brillouin sampling volume was fully contained within the cell body by carefully inspecting bright‐field images (Figure S12, Supporting Information). The different spatial resolutions achieved in the mechanical maps using the two configurations are reported in Figure S13 (Supporting Information).

Brillouin shifts and linewidths were obtained by fitting the spectra with a damped harmonic oscillator DHO function.^[^ ^15^ ^]^ A custom Python script was used to, first, fit separately the Stokes and anti‐Stokes peaks, to verify the symmetry with respect to the elastic peak. In case of asymmetry, the x‐axis was shifted to obtain this condition. Then, a single DHO was used to fit the entire spectrum, and the resulting parameters were used for the analysis.

The Raman analysis was focused on the high‐frequency region where the CH stretching bands (centered at 2900 cm^−1^) and the OH stretching band (centered ≈ 3300 cm^−1^) are located. The spectra were analysed using a custom Python script, aiming to extract the ratio between the areas of the CH and OH bands, to quantify relative compositional changes across the different configurations. The processing steps were as follows: a) a spline function was used to remove the laser‐induced fluorescence, b) a spectrum acquired on an empty PDMS chip was subtracted to eliminate the PDMS contribution; c) since the CH and OH bands overlap, their contributions were separated by subtracting a reference spectrum acquired from the cell culture medium, which contained only the OH band. This subtraction removed the OH contribution and isolated the CH band. The areas of the isolated CH and OH bands were then evaluated and used to calculate their ratio. The normalization factors for the medium and PDMS subtractions ware determined by minimizing the difference between the resulting CH band area and the CH band area from a reference spectrum of a spheroid acquired outside the chip.

Brillouin‐Raman spectra analysed 8–38 spheroids, with at least one spectrum acquired per spheroid. The dots reported in the figures correspond to the mean frequency shift, linewidth and CH/OH peak area within the measured spheroids. The data points represent individual spheroids and are shown to represent biological variability. When more than one acquisition per spheroid was performed within the respective time point (e.g., during time‐course analyses of recovery), values were averaged to provide a representative measure.

To couple microfluidics with BRmS, we set up the pump next to the imaging system and introduced spheroids into the deformation recovery device at 3 mL h^−1^. Spheroids were captured in the recovery traps, the flow was stopped and the spheroids were imaged using Brillouin and Raman microscopy.

### Timelapse Imaging of Spheroids Moving Through Constriction Channels

Micro‐devices were coated with anti‐adhesion solution (StemCell) for 15 min at RT. Channels were washed with cell culture media. The microscope incubator was set to 37 °C. Spheroids were introduced into the microfluidic channels at a concentration of 1200 per 2 mL of media (containing 25 mM Hepes).

This low concentration enables tracking of individual spheroids through the channels. Spheroids were tracked using a Thunder Leica microscope and a Camera (Photometrics Prime BSI Express sCMOS) camera. Videos were created using a 5X objective using the brightfield channel and obtaining 4–6 ms time step sizes.

### Dextran Assay

Spheroids were collected in MCF10.DCIS.com media containing 25 mM hepes and 0.2% gentamycin. Control spheroids were weakly attached to the base of an 8‐well Ibidi‐treat dish (Ibidi) by incubating for 1.5 h. Spheroids were then incubated with 3 kDa TRITC dextran (Thermofisher) concentration of 2 g L^−1^ in 200 µL total volume of media for 20 min to fill the interstitial volume with fluorescent dextran (Figure S6, Supporting Information). Timelapse videos were taken of the spheroid central plane using a 40x air objective and a 30 s time step using a Leica TCS SP8‐STED confocal microscope.

Long‐channel devices were treated with anti‐adhesion solution (StemCell) for 15 min and then pre‐washed with media containing 2 g L^−1^ dextran dye. Spheroids were incubated with the dextran dye for 20 min and then introduced into the long‐channel device at 3 mL h^−1^, then reduced to 1 mL h^−1^ and captured in the 40 µm gap zone for imaging. Spheroid z‐stacks were obtained with 0.8 µm step size and 40x air objective.

### Statistical Analyses

All statistical analyses were performed using GraphPad Prism software. Data are mean ± s.d. of biological replicates. For comparison between two groups, statistics were performed with an unpaired two‐tailed Student's t‐test for normally distributed populations. The Mann Whitney t‐test was applied for non‐normally distributed samples. Normality tests were performed with the Shapiro‐Wilk test (alpha = 0.05). For comparison between more than two groups, data were analyzed by ordinary one‐way or two‐way ANOVA and 95% confidence intervals; with Tukey's correction for comparison between all groups, and Dunnett's multiple comparison when comparing groups against the control. Experiments were performed in triplicate.

## Conflict of Interest

The authors declare no conflict of interest.

## Author Contributions

M.M., A.A.P., and D.L. are co‐first authors and equally contributed to this work. B.J.G. is the lead contact and B.J.G. performed conceptualization, methodology, investigation, validation, formal analysis, writing, visualization, supervision, and funding acquisition. G.S. and S.C. performed conceptualization, methodology, writing, visualization, supervision, funding acquisition, and resources. A.A.P., M.M., D.L., and R.K.A.S. performed methodology, investigation, validation, and formal analysis. S.M., A.D., H.A., M. M., and F.B. performed methodology, validation, supervision, and funding acquisition. E.M., M.T., S.M., and F.O. performed software, resources, and data curation. E.B. performed software, data curation, and resources. C.M., L.D., and J.P. performed methodology, investigation, and validation. M.P., D.F., and S.M. performed supervision and resources.

## Supporting information



Supporting Information

Supporting Information

Supporting Information

Supporting Information

Supporting Information

Supporting Information

Supporting Information

Supporting Information

Supporting Information

## Data Availability

The data that support the findings of this study are available from the corresponding author upon reasonable request.

## References

[1] K. Damodaran , S. Venkatachalapathy , F. Alisafaei , A. V. Radhakrishnan , D. Sharma Jokhun , V. B. Shenoy , G. V. Shivashankar , Mol. Biol. Cell 2018, 29, 3039. 30256731 10.1091/mbc.E18-04-0256 PMC6333178

[2] J. J. Northey , L. Przybyla , V. M. Weaver , Cancer Discov. 2017, 7, 1224. 29038232 10.1158/2159-8290.CD-16-0733 PMC5679454

[3] H. Yu , J. K. Mouw , V. M. Weaver , Trends Cell Biol. 2011, 21, 47. 20870407 10.1016/j.tcb.2010.08.015 PMC3014395

[4] E. Frittoli , A. Palamidessi , F. Iannelli , F. Zanardi , S. Villa , L. Barzaghi , H. Abdo , V. Cancila , G. V. Beznoussenko , G. Della Chiara , M. Pagani , C. Malinverno , D. Bhattacharya , F. Pisati , W. Yu , V. Galimberti , G. Bonizzi , E. Martini , A. A. Mironov , U. Gioia , F. Ascione , Q. Li , K. Havas , S. Magni , Z. Lavagnino , F. A. Pennacchio , P. Maiuri , S. Caponi , M. Mattarelli , S. Martino , et al., Nat. Mater. 2023, 22, 644. 36581770 10.1038/s41563-022-01431-x PMC10156599

[5] H. T. Nia , L. L. Munn , R. K. Jain , Science 2020, 370, aaz0868. 10.1126/science.aaz0868 PMC8274378 33122355

[6] X. Quiroga , N. Walani , A. Disanza , A. Chavero , A. Mittens , F. Tebar , X. Trepat , R. G. Parton , M. I. Geli , G. Scita , M. Arroyo , A.‐L. Le Roux , P. Roca‐Cusachs , Elife 2023, 12, 72316. 10.7554/eLife.72316 PMC10569792 37747150

[7] C. J. Bustamante , Y. R. Chemla , S. Liu , M. D. Wang , Nat. Rev. Methods Prim. 2021, 1, 25. 10.1038/s43586-021-00021-6 PMC8629167 34849486

[8] A. R. Bausch , W. Moller , E. Sackmann , Biophys. J. 1999, 76, 573. 9876170 10.1016/S0006-3495(99)77225-5 PMC1302547

[9] B. K. Patel , K. Pepin , K. R. Brandt , G. L. Mazza , B. A. Pockaj , J. Chen , Y. Zhou , D. W. Northfelt , K. Anderson , J. M. Kling , C. M. Vachon , K. R. Swanson , M. Nikkhah , R. Ehman , Breast Cancer Res. Treat. 2022, 194, 79. 35501423 10.1007/s10549-022-06607-2 PMC9538705

[10] M. H. Panhwar , F. Czerwinski , V. A. S. Dabbiru , Y. Komaragiri , B. Fregin , D. Biedenweg , P. Nestler , R. H. Pires , O. Otto , Nat. Commun. 2020, 11, 2190. 32366850 10.1038/s41467-020-15813-9 PMC7198589

[11] I. Kabakova , J. Zhang , Y. Xiang , S. Caponi , A. Bilenca , J. Guck , G. Scarcelli , Nat. Rev. Methods Prim. 2024, 4, 8. 10.1038/s43586-023-00286-z PMC11465583 39391288

[12] S. Caponi , A. Passeri , G. Capponi , D. Fioretto , M. Vassalli , M. Mattarelli , Eur. Biophys. J. 2022, 51, 99. 34463775 10.1007/s00249-021-01567-9 PMC8964566

[13] A. Malandrino , R. D. Kamm , E. Moeendarbary , ACS Biomater. Sci. Eng. 2018, 4, 294. 29457129 10.1021/acsbiomaterials.7b00041 PMC5811931

[14] P. Bouvet , et al., Nat. Photon. 2024, 19, 681.

[15] S. Mattana , M. Mattarelli , L. Urbanelli , K. Sagini , C. Emiliani , M. D. Serra , D. Fioretto , S. Caponi , Light Sci. Appl. 2018, 7, 17139. 30839528 10.1038/lsa.2017.139 PMC6060066

[16] G. Scarcelli , W. J. Polacheck , H. T. Nia , K. Patel , A. J. Grodzinsky , R. D. Kamm , S. H. Yun , Nat. Methods 2015, 12, 1132. 26436482 10.1038/nmeth.3616 PMC4666809

[17] A. Fasciani , S. D'Annunzio , V. Poli , L. Fagnocchi , S. Beyes , D. Michelatti , F. Corazza , L. Antonelli , F. Gregoretti , G. Oliva , R. Belli , D. Peroni , E. Domenici , S. Zambrano , D. Intartaglia , C. Settembre , I. Conte , C. Testi , P. Vergyris , G. Ruocco , A. Zippo , Nat. Genet. 2020, 52, 1397. 33169020 10.1038/s41588-020-00724-8 PMC7610431

[18] S. Kerdegari , A. A. Passeri , F. Morena , G. Ciccone , V. Bazzurro , P. Canepa , A. Lagomarsino , S. Martino , M. Mattarelli , M. Vassalli , A. Diaspro , S. Caponi , C. Canale , Acta Biomater. 2025, 198, 291. 40189116 10.1016/j.actbio.2025.04.009

[19] M. Nikolic , G. Scarcelli , K. Tanner , Biophys. J. 2022, 121, 3586. 36059196 10.1016/j.bpj.2022.09.002 PMC9617162

[20] G. Yan , S. Monnier , M. Mouelhi , T. Dehoux , Proc. Natl. Acad. Sci. U S A 2022, 119, 2113614119. 10.1073/pnas.2113614119 PMC8795543 35046032

[21] V. Mahajan , T. Beck , P. Gregorczyk , A. Ruland , S. Alberti , J. Guck , C. Werner , R. Schlüßler , A. V. Taubenberger , Cancers (Basel) 2021, 13, 5549. 34771711 10.3390/cancers13215549 PMC8583550

[22] K. Elsayad , S. Werner , M. Gallemí , J. Kong , E. R. Sánchez Guajardo , L. Zhang , Y. Jaillais , T. Greb , Y. Belkhadir , Sci. Signal 2016, 9, rs5. 27382028 10.1126/scisignal.aaf6326

[23] M. Mattarelli , G. Capponi , A. A. Passeri , D. Fioretto , S. Caponi , ACS Photonics 2022, 9, 2087.

[24] G. Antonacci , R. Vanna , M. Ventura , M. L. Schiavone , C. Sobacchi , M. Behrouzitabar , D. Polli , C. Manzoni , G. Cerullo , Nat. Commun. 2024, 15, 5202. 38898004 10.1038/s41467-024-49419-2 PMC11187154

[25] A. A. Passeri , F. Morena , C. Argentati , F. Bonacci , I. Neri , D. Fioretto , M. Vassalli , S. Martino , M. Mattarelli , S. Caponi , ACS Photonics 2025, 12, 3794. 40688183 10.1021/acsphotonics.5c00808 PMC12272693

[26] F. Scarponi , S. Mattana , S. Corezzi , S. Caponi , L. Comez , P. Sassi , A. Morresi , M. Paolantoni , L. Urbanelli , C. Emiliani , L. Roscini , L. Corte , G. Cardinali , F. Palombo , J. ? R. Sandercock , D. Fioretto , Phys. Rev. X 2017, 7, 031015.

[27] L. Qiu , Y. Su , K.‐M. Xu , H. Cui , D. Zheng , Y. Zhu , L. Li , F. Li , W. Zhao , Light Sci. Appl. 2023, 12, 129. 37248287 10.1038/s41377-023-01153-y PMC10226997

[28] M. Alunni Cardinali , A. Di Michele , M. Mattarelli , S. Caponi , M. Govoni , D. Dallari , S. Brogini , F. Masia , P. Borri , W. Langbein , F. Palombo , A. Morresi , D. Fioretto , J. R. Soc. Interface 2022, 19, 20210642. 35104431 10.1098/rsif.2021.0642 PMC8807060

[29] A. A. Passeri , A. Di Michele , I. Neri , F. Cottone , D. Fioretto , M. Mattarelli , S. Caponi , Biomater. Adv. 2023, 147, 213341. 36827851 10.1016/j.bioadv.2023.213341

[30] R. Mercatelli , S. Mattana , L. Capozzoli , F. Ratto , F. Rossi , R. Pini , D. Fioretto , F. S. Pavone , S. Caponi , R. Cicchi , Commun. Biol. 2019, 2, 117. 30937399 10.1038/s42003-019-0357-y PMC6435656

[31] M. Urbanska , H. E. Muñoz , J. Shaw Bagnall , O. Otto , S. R. Manalis , D. Di Carlo , J. Guck , Nat. Methods 2020, 17, 587. 32341544 10.1038/s41592-020-0818-8 PMC7275893

[32] B. J. Green , M. Marazzini , B. Hershey , A. Fardin , Q. Li , Z. Wang , G. Giangreco , F. Pisati , S. Marchesi , A. Disanza , E. Frittoli , E. Martini , S. Magni , G. V. Beznoussenko , C. Vernieri , R. Lobefaro , D. Parazzoli , P. Maiuri , K. Havas , M. Labib , S. Sigismund , P. P. D. Fiore , R. H. Gunby , S. O. Kelley , G. Scita , Small 2022, 18, 2106097. 10.1002/smll.202106097 35344274

[33] Z. Chen , Y. Zhu , D. Xu , Md. M. Alam , L. Shui , H. Chen , Lab Chip 2020, 20, 2343. 32463051 10.1039/d0lc00092b

[34] R. C. Boot , A. Roscani , L. van Buren , S. Maity , G. H. Koenderink , P. E. Boukany , Lab Chip 2023, 23, 1768. 36809459 10.1039/d2lc01060g PMC10045894

[35] S. Jain , H. Belkadi , A. Michaut , S. Sart , J. Gros , M. Genet , C. N. Baroud , Biofabrication 2024, 16, 035010. 10.1088/1758-5090/ad30c7 38447213

[36] M. Pandey , Y. J. Suh , M. Kim , H. J. Davis , J. E. Segall , M. Wu , Phys. Biol. 2024, 21, 036003. 10.1088/1478-3975/ad3ac5 PMC12950161 38574674

[37] G. Mary , B. Malgras , J. Efrain Perez , I. Nagle , N. Luciani , C. Pimpie , A. Asnacios , M. Pocard , M. Reffay , C. Wilhelm , Cancers (Basel) 2022, 14, 366. 35053529 10.3390/cancers14020366 PMC8773997

[38] M. Dolega , G. Zurlo , M. L. Goff , M. Greda , C. Verdier , J.‐F. Joanny , G. Cappello , P. Recho , J. Mech. Phys. Solids 2021, 147, 104205.

[39] N. Aceto , A. Bardia , D. T. Miyamoto , M. C. Donaldson , B. S. Wittner , J. A. Spencer , M. Yu , A. Pely , A. Engstrom , H. Zhu , B. W. Brannigan , R. Kapur , S. L. Stott , T. Shioda , S. Ramaswamy , D. T. Ting , C. P. Lin , M. Toner , D. A. Haber , S. Maheswaran , Cell 2014, 158, 1110. 25171411 10.1016/j.cell.2014.07.013 PMC4149753

[40] F. R. Miller , S. J. Santner , L. Tait , P. J. Dawson , J. Natl. Cancer Inst. 2000, 92, 1185a. 10.1093/jnci/92.14.1185a 10904098

[41] C. M. Denais , R. M. Gilbert , P. Isermann , A. L. McGregor , M. te Lindert , B. Weigelin , P. M. Davidson , P. Friedl , K. Wolf , J. Lammerding , Science 2016, 352, 353. 27013428 10.1126/science.aad7297 PMC4833568

[42] P. Shah , C. M. Hobson , S. Cheng , M. J. Colville , M. J. Paszek , R. Superfine , J. Lammerding , Curr. Biol. 2021, 31, p753. 10.1016/j.cub.2020.11.037 PMC7904640 33326770

[43] Y. Javanmardi , A. Agrawal , A. Malandrino , S. Lasli , M. Chen , S. Shahreza , B. Serwinski , L. Cammoun , R. Li , M. Jorfi , B. Djordjevic , N. Szita , F. Spill , S. Bertazzo , G. K. Sheridan , V. Shenoy , F. Calvo , R. Kamm , E. Moeendarbary , Adv. Sci. (Weinh) 2023, 10, 2206554. 37051804 10.1002/advs.202206554 PMC10238207

[44] V. Gensbittel , M. Kräter , S. Harlepp , I. Busnelli , J. Guck , J. G. Goetz , Dev. Cell 2021, 56, 164. 33238151 10.1016/j.devcel.2020.10.011

[45] S. H. Au , B. D. Storey , J. C. Moore , Q. Tang , Y.‐L. Chen , S. Javaid , A. F. Sarioglu , R. Sullivan , M. W. Madden , R. O'Keefe , D. A. Haber , S. Maheswaran , D. M. Langenau , S. L. Stott , M. Toner , Proc. Natl. Acad. Sci. U S A 2016, 113, 4947. 27091969 10.1073/pnas.1524448113 PMC4983862

[46] L. Beunk , G.‐J. Bakker , D. van Ens , J. Bugter , F. Gal , M. Svoren , P. Friedl , K. Wolf , Eur. Phys. J. E Soft Matter 2022, 45, 48. 35575822 10.1140/epje/s10189-022-00182-6 PMC9110550

[47] L. Beunk , K. Brown , I. Nagtegaal , P. Friedl , K. Wolf , Semin. Cell Dev. Biol. 2019, 93, 36. 30009945 10.1016/j.semcdb.2018.07.014 PMC6399078

[48] C. Colson , F. Sanchez‐Garduno , H. M. Byrne , P. K. Maini , T. Lorenzi , Proc. Math Phys. Eng. Sci. 2021, 477, 20210593. 35153606 10.1098/rspa.2021.0593 PMC8791052

[49] H. T. Nia , H. Liu , G. Seano , M. Datta , D. Jones , N. Rahbari , J. Incio , V. P. Chauhan , K. Jung , J. D. Martin , V. Askoxylakis , T. P. Padera , D. Fukumura , Y. Boucher , F. J. Hornicek , A. J. Grodzinsky , J. W. Baish , L. L. Munn , R. K. Jain , Nat. Biomed. Eng. 2016, 1, 0004. 28966873 10.1038/s41551-016-0004 PMC5621647

[50] G. Guan , R. D. Cannon , D. E. Coates , L. Mei , Genes (Basel) 2023, 14, 272. 36833199 10.3390/genes14020272 PMC9957420

[51] J. D. Amack , M. L. Manning , Science 2012, 338, 212. 23066072 10.1126/science.1223953

[52] A. J. Devanny , M. B. Vancura , L. J. Kaufman , Mol. Biol. Cell 2021, 32, ar24. 34432511 10.1091/mbc.E21-07-0357 PMC8693969

[53] D. Fan , E. E. Creemers , Z. Kassiri , Circ. Res. 2014, 114, 889. 24577968 10.1161/CIRCRESAHA.114.302335

[54] I. G. Goncalves , J. M. Garcia‐Aznar , PLoS Comput. Biol. 2021, 17, 1008764. 10.1371/journal.pcbi.1008764 PMC7968691 33635856

[55] M. Takeuchi , S. Kajimoto , T. Nakabayashi , J. Phys. Chem. Lett. 2017, 8, 5241. 29022721 10.1021/acs.jpclett.7b02154

[56] A. Ghita , T. Hubbard , P. Matousek , N. Stone , Anal. Chem. 2020, 92, 9449. 32603089 10.1021/acs.analchem.0c01842

[57] A. B. Fulton , Cell 1982, 30, 345.6754085

[58] M. Mattarelli , M. Vassalli , S. Caponi , ACS Photonics 2020, 7, 2319. 10.1021/acsphotonics.5c00808 PMC12272693 40688183

[59] M. E. Dolega , S. Monnier , B. Brunel , J.‐F. Joanny , P. Recho , G. Cappello , Elife 2021, 10, 63258. 10.7554/eLife.63258 PMC8064752 33704063

[60] M. Bailey , M. Alunni‐Cardinali , N. Correa , S. Caponi , T. Holsgrove , H. Barr , N. Stone , C. P. Winlove , D. Fioretto , F. Palombo , Sci. Adv. 2020, 6, abc1937. 10.1126/sciadv.abc1937 PMC7608813 33127678

[61] E. M. Darling , D. Di Carlo , Annu. Rev. Biomed. Eng. 2015, 17, 35. 26194428 10.1146/annurev-bioeng-071114-040545 PMC8204286

[62] Y. Song , J. Soto , B. Chen , T. Hoffman , W. Zhao , N. Zhu , Q. Peng , L. Liu , C. Ly , P. K. Wong , Y. Wang , A. C. Rowat , S. K. Kurdistani , S. Li , Nat. Mater. 2022, 21, 1191. 35927431 10.1038/s41563-022-01312-3 PMC9529815

[63] M. M. Nava , Y. A. Miroshnikova , L. C. Biggs , D. B. Whitefield , F. Metge , J. Boucas , H. Vihinen , E. Jokitalo , X. Li , J. M. García Arcos , B. Hoffmann , R. Merkel , C. M. Niessen , K. N. Dahl , S. A. Wickström , Cell 2020, 181, 800. 32302590 10.1016/j.cell.2020.03.052 PMC7237863

[64] R. Golloshi , C. Playter , T. F. Freeman , P. Das , T. I. Raines , J. H. Garretson , D. Thurston , R. P. McCord , EMBO Rep. 2022, 23, 52149. 10.15252/embr.202052149 PMC9535800 35969179

[65] A. D. Santos , C. P. Toseland , Int. J. Mol. Sci. 2021, 22, 3178.33804722

[66] M. Payson , M. Malik , S. Siti‐nur Morris , J. H. Segars , R. Chason , W. H. Catherino , Fertil. Steril. 2009, 92, 748. 18692824 10.1016/j.fertnstert.2008.06.030 PMC4130340

[67] L. M. Hoffman , M. A. Smith , C. C. Jensen , M. Yoshigi , E. Blankman , K. S. Ullman , M. C. Beckerle , Mol. Biol. Cell 2020, 31, 1774. 31967947 10.1091/mbc.E19-01-0027 PMC7521858

[68] S. Calamak , M. Ermis , H. Sun , S. Islam , M. Sikora , M. Nguyen , V. Hasirci , L. M. Steinmetz , U. Demirci , Adv. Biosyst. 2020, 4, 1900139. 10.1002/adbi.201900139 32293132

[69] X. Yin , C. C. Wolford , Y.‐S. Chang , S. J. McConoughey , S. A. Ramsey , A. Aderem , T. Hai , J. Cell Sci. 2010, 123, 3558. 20930144 10.1242/jcs.064915 PMC2951469

[70] S. Cantoni , S. Cavalli , F. Pastore , A. Accetta , D. Pala , F. Vaccaro , N. Cesari , F. De Logu , R. Nassini , G. Villetti , F. Facchinetti , Eur. J. Pharmacol. 2019, 850, 126. 30753868 10.1016/j.ejphar.2019.02.009

