# Supplementary material for: Brillouin Microscopy of Breast tumor Spheroids On‐a‐Chip: Mechanical and Transcriptional Responses to Microfluidic‐Induced Rapid Deformations
Source: Adv Sci (Weinh). 2025 Dec 16;13(4):e13153. doi: 10.1002/advs.202513153 (PMC12822390; doi:10.1002/advs.202513153)
Supplement: Supplementary file 1 — Supporting Information [file ADVS-13-e13153-s009.pdf]

SUPPLEMENTARY INFORMATION

Brillouin Microscopy of breast tumor spheroids On-a-Chip: Mechanical and Transcriptional Responses to Microfluidic-Induced Rapid Deformations

Mona Makkieh[1§], Alessandra Anna Passeri[2§], Davide Lazzari[1§], Stefano Marchesi[1], Andrea Disanza[1,3], Rafal Khaled Ahmad Salem[1], Francesco Bonacci[2], Camillo Mazzella[1], Emanuele Martini[1], Mattia Tonani[1], Leonardo Donati[4], Edoardo Bellini[1], Hind Abdo[1], Judith Pineau[5], Serena Magni[1], Fabrizio Orsenigo[1], Matthieu Piel[5], Daniele Fioretto[2,7], Sabata Martino[4,6], Maurizio Mattarelli[2], Giorgio Scita[1,3], Brenda J. Green[1,3*], Silvia Caponi[7*]*

[1] IFOM ETS - The AIRC Institute of Molecular Oncology, Via Adamello, 16, 20139 Milan, Italy.
[2] Department of Physics and Geology, University of Perugia, Via A. Pascoli, 06123 Perugia, Italy
[3] Department of Oncology and Haemato-Oncology, University of Milan, Milan, Italy.
[4] Department of Chemistry, Biology, and Biotechnologies, Via del Giochetto, University of Perugia, Perugia, Italy
[5] CNRS UMR144, Institut Curie, Institut Pierre Gilles de Gennes, PSL Research University, Paris, France
[6] Centro di Eccellenza Materiali Innovativi Nanostrutturati per Applicazioni Chimiche Fisiche e Biomediche (CEMIN), University of Perugia, 06123 Perugia, Italy
[7] National Research Council of Italy, Istituto Officina dei Materiali CNR - IOM, c/o Department of Physics and Geology, University of Perugia, Via A. Pascoli, 06123 Perugia, Italy

*Corresponding authors: brenda.green@ifom.eu; giorgio.scita@ifom.eu; silvia.caponi@cnr.it
Lead contact: brenda.green@ifom.eu
*shared last author position

[§]shared first author position

Keywords: Brillouin microscopy, Raman microscopy, microfluidics, fluorescence imaging, breast cancer, spheroids

CONTENTS

FIGURES

Figure S1. Microfluidic setup for re-circulation of spheroids through deformation events.

Figure S2. Compressed spheroid Comsol modeling and shape characterization.

Figure S3. Schematic of a spheroid moving through the deformation device.

Figure S4. Comsol modeling of the flow control device.

Figure S5. Linear correlation regression for control, ROCK inhibitor- treated and RhoA activator-treated spheroid diameters.

Figure S6. Quantification of interstitial volume in spheroids using fluorescent dextran.

Figure S7. Spheroid packing density and interstitial spaces of MCF10.DCIS.com spheroids treated with a ROCK inhibitor or RhoA activator.

Figure S8. Comsol modeling of the long-channel device.

Figure S9. Device set up for measurement of dextran in compressed spheroids.

Figure S10. Comsol modeling of deformation and flow recovery devices.

Figure S11. Spheroid recovery dynamics.

Figure S12. Brightfield images of MCF10.DCIS.com cells within a spheroids.

Figure S13: Brillouin frequency shifts and linewidth maps of MCF10.DCIS.com spheroids using 20x and 60x objectives.

Figure S14. Raman analysis of 1-pass and 4-pass spheroids in the recovery traps over time.

Figure S15. Brillouin microscopy measurements for flow control spheroids.

Figure S16. Spheroid transit times through constrictions.

Figure S17. Aqua dye membrane integrity assay.

Figure S18. Cytoplasmic live cell dye assay confirms viability 24h post- deformation.

Figure S19. Ki67 immunohistochemistry of spheroids 24h post-deformation.

Figure S20. Nuclear aspect ratios from the mid-plane of spheroids in-channel and post-chip.

Figure S21. MCF10.DCIS.com nuclear and membrane labeled cells in spheroids.

Figure S22. 3D segmentation of nuclei and cells in MCF10.DCIS.com spheroids.

Figure S23. Volcano plots for MCF10.DCIS.com spheroids.

Figure S24. Hallmark pathways for MCF10.DCIS.com spheroids.

Figure S25. ATF3 levels increase following deformation of MCF10.DCIS.com, HaCaT and MCF10A spheroids.

Figure S26. Panel images of collagen invasion of MCF10.DCIS.com spheroids.

TABLES

Table S1: iBright western blot volume measurements

Table S2: Collagen Reagent Amounts

Table S3: ANCOVA analysis of MCF10.DCIS.com spheroid diameters vs transit times for no-treatment controls, spheroids treated with a ROCK inhibitor or a RhoA activator

VIDEOS

Video S1. MCF10.DCIS.com spheroid in the deformation device.
The spheroid was introduced into the deformation device at 3 ml/h.

Video S2. MCF10.DCIS.com spheroid treated with ROCK inhibitor in the deformation device.
Cells in spheroids were treated with 100 nM ROCK 1/2 for 24 h, and then introduced into the deformation device at 3 ml/h.

Video S3. MCF10.DCIS.com spheroid treated with RhoA activator in the deformation device.
Cells in spheroids were treated with 10 μg/ml RhoA activator (CN03) for 24 h, and then introduced into the deformation device at 3 ml/h.

Video S4. MCF10.DCIS.com spheroids entering the recovery traps.
Spheroids were introduced into the deformation device at 3 ml/h. The scale bar is 500 μm. The minimal debris in the capture zone does not impact capture or analysis.

Video S5. MCF10.DCIS.com spheroid recovering in the trap (Brightfield).
The spheroid was deformed 1-cycle through the device at 3 ml/h and captured in the traps.

Video S6. MCF10.DCIS.com spheroid recovering in the trap (GFP-H2B).
The spheroid was deformed 1-cycle through the device at 3 ml/h. Cells expresses fluorescent GFP-H2B. This spheroid is identical to the spheroid shown in Video S5.

Video S7. MCF10.DCIS.com compressed spheroid with segmented nuclei.
Arivis 3D re-construction of MCF10.DCIS.com spheroid nuclei in the constriction channel. The nuclei were resolved in half the spheroid (closer to the base) and these were used in the analysis.

Video S8. MCF10.DCIS.com control spheroid with segmented nuclei.
Arivis 3D re-construction of MCF10.DCIS.com spheroid nuclei. The lower half of the spheroid was used for analysis, to correspond with the compressed spheroid image processing.

EXPERIMENTAL SECTION

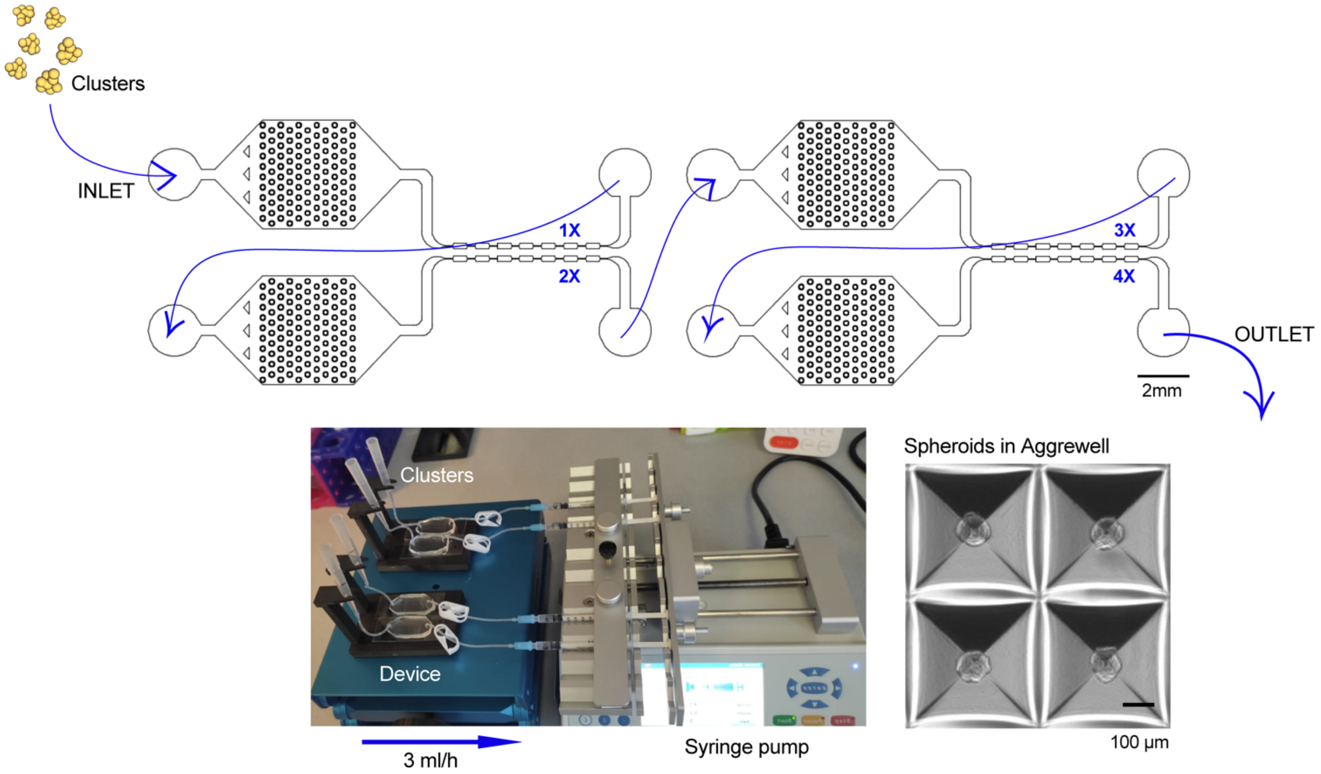

Figure S1. **Microfluidic setup for re-circulation of spheroids through deformation events.** Spheroids are grown in Aggrewell low attachment dishes, collected in cell culture media and introduced into the device. Spheroids can be cycled through the device. Each cycle (1X, 2X, 3X, 4X) takes 0.8s (device passage time) and 10 minutes recirculation time.

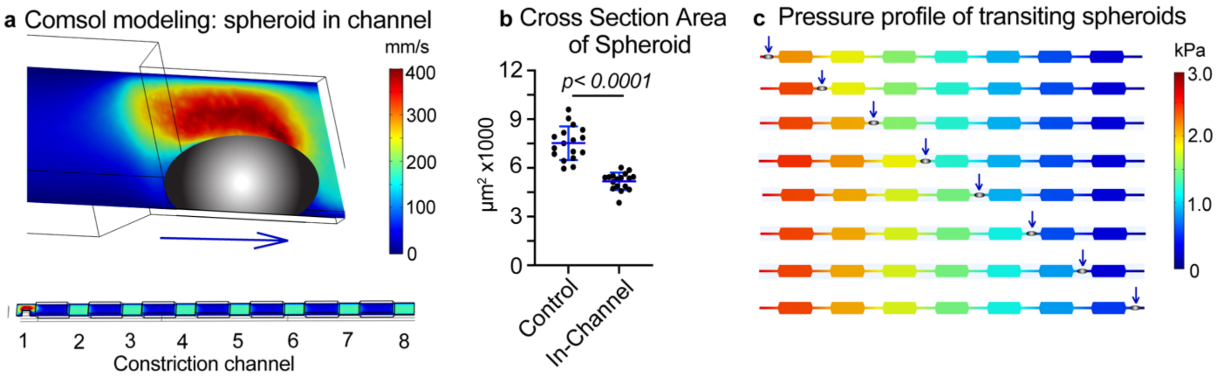

Figure S2. Compressed spheroid Comsol modeling and shape characterization.
a, A spheroid modeled in the deformation channel. Simulation of velocity profiles are performed with a constant flow rate of 3 ml/h. The spheroid was modeled in the channel as an ellipsoid considering dimensions of 90 µm height and 130 µm length and 40 µm width. **b**, cross-sectional area of compressed spheroids relative to controls obtained through confocal z-sectioning. Mean ± SD are shown. $p<0.05$ are considered significant. Dots represent spheroids (N= 17 control and 17 in-channel spheroids). The cross section area considers the area of an ellipse = π x ½ (width) x ½ (height). **c**, Comsol pressure profiles across the channel, obtained when a spheroid is in each constriction channel.

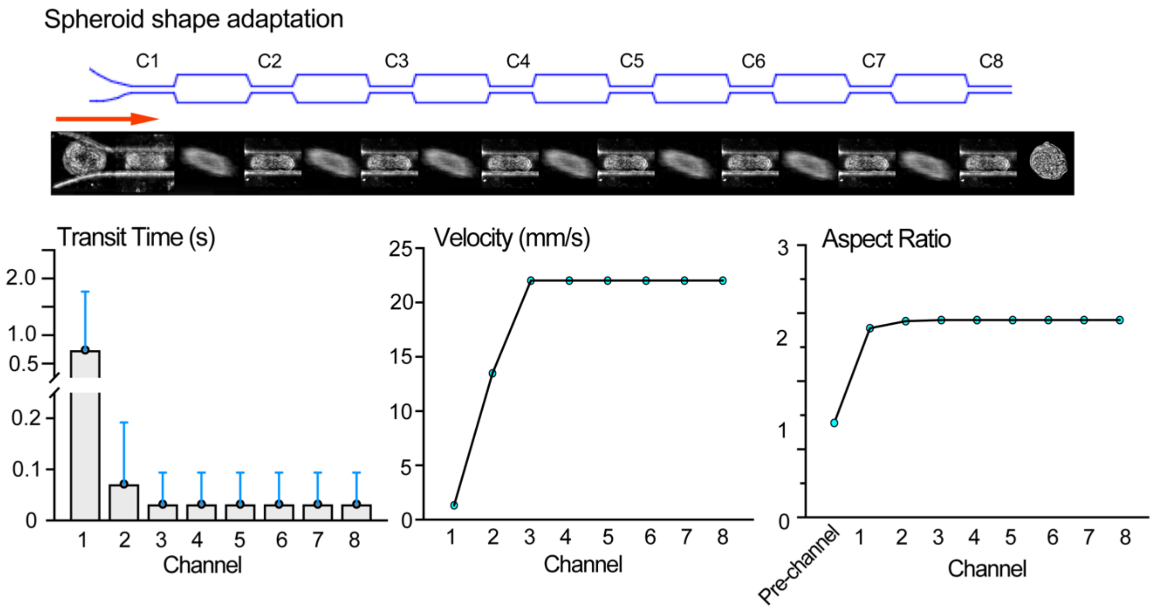

Figure S3. Schematic of a spheroid moving through the deformation device. Spheroid transit times, velocity and aspect ratios are plotted for each constriction channel. Data are obtained experimentally by tracking spheroids passing through the channels using widefield timelapse microscopy. The microscopy imaging allows visualization of the first three channels using the lowest objective. In these plots, it was assumed that the parameters obtained for C4-8 are equal to the parameters for C3. The transit time and velocity for C1 includes the pre-channel event as soon as the spheroid arrives at the channel entrance, and is tracked through C1. The aspect ratio of the spheroid before it enters C1 is included, to highlight the initial shape change. For transit time, 127 spheroids were analyzed. For the channel velocity, 126 spheroids were analyzed and for the aspect ratio, 23 spheroids were analyzed. Mean values + SD or only mean values are shown.

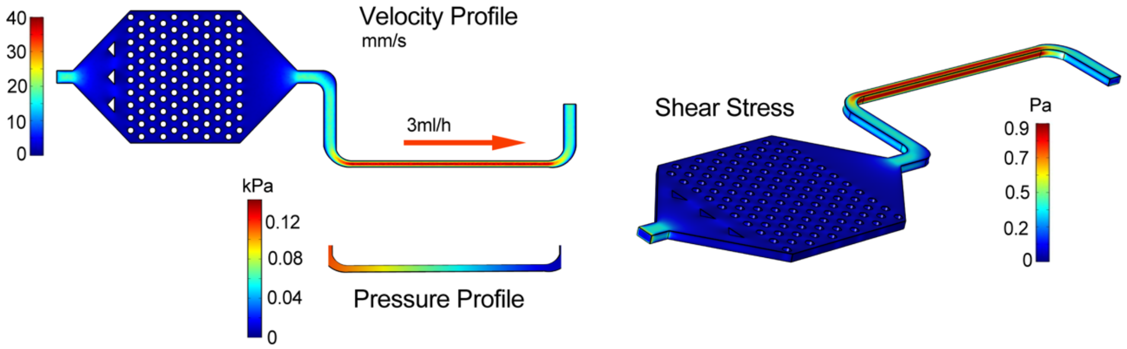

Figure S4. **Comsol modeling of the flow control device.** Fluid modeling was performed considering a flow rate of 3 ml/h. Velocity, pressure and shear stress profiles are shown.

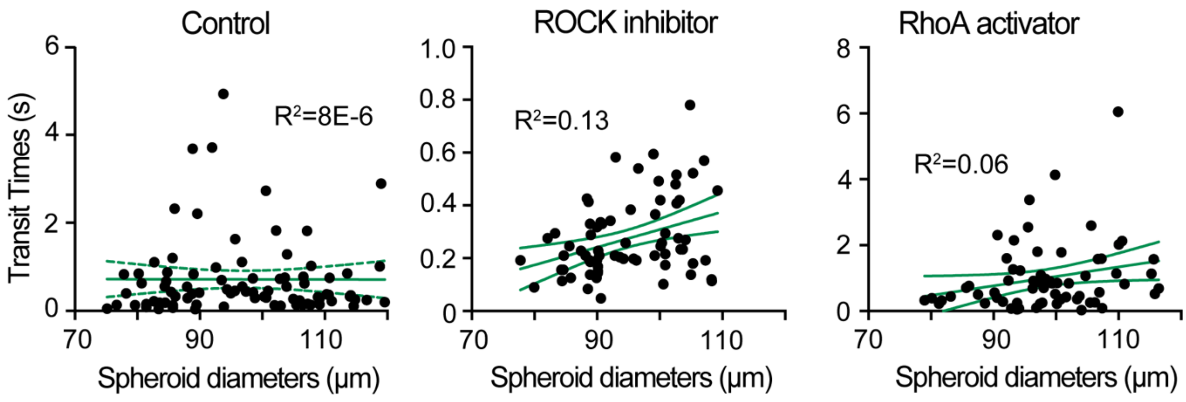

Figure S5. Linear correlation regression for control, ROCK inhibitor- treated and RhoA activator- treated spheroid diameters. Linear regression line and error bars are shown in green. Each dot represents a spheroid; n= 84, 70 and 64 control, ROCK inhibitor and RhoA activator-treated spheroids, respectively. Spheroid diameters were measured prior to entering C1. Transit times were obtained from timelapse imaging.

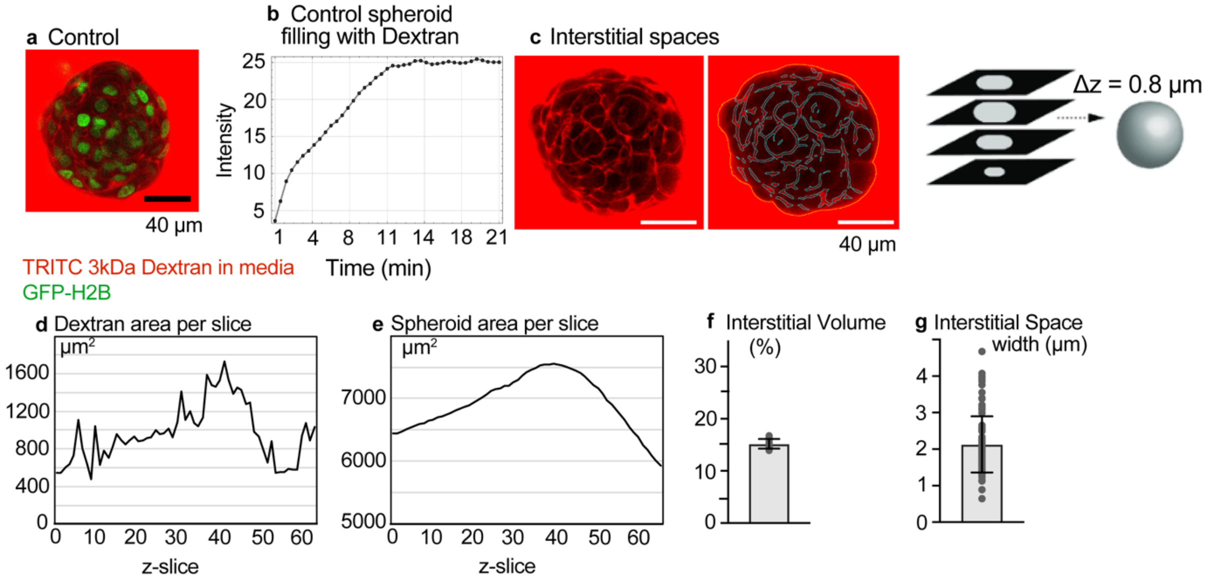

Figure S6. Quantification of interstitial volume in spheroids using fluorescent dextran.
a, Confocal images of control spheroids in the dextran TRITC dye. Spheroids stably expressed GFP-H2B for recognizing nuclei. **b**, Timelapse analysis of dextran uptake into control spheroids. The plot was obtained by tracing the spheroid mid-plane and plotting the intensity against time for the timelapse video. **c**, Fluorescently labeled dextran dye diffuses into the interstitial spaces in control spheroids in approximately 11-12 minutes. Confocal images are shown at the spheroid mid-plane, and the automated detection of the dextran in interstitial spaces is illustrated on the right. Z-stacks are obtained of the spheroid and an automated macro is applied to identify the interstitial space area per slice (see Methods). **d-e**, Spheroid dextran area per slice and spheroid slice area are shown, respectively, for a representative control spheroid with a diameter of 97 µm. **f**, The percent interstitial volume ($\Phi_{ECM} = 15\%$) in the spheroid relative to the total volume (N= 10 spheroids). This estimation is in the range of percent interstitial volume (14%) reported in breast tumor spheroids[1]. Mean ± SD are shown. Dots represent spheroids. **g**, The spheroid interstitial space widths (µm) are plotted, as estimated from spheroids filled with dextran in the mid-plane of the spheroid. N= 86 spheroids. Mean ± SD are shown. Dots represent interstitial widths.

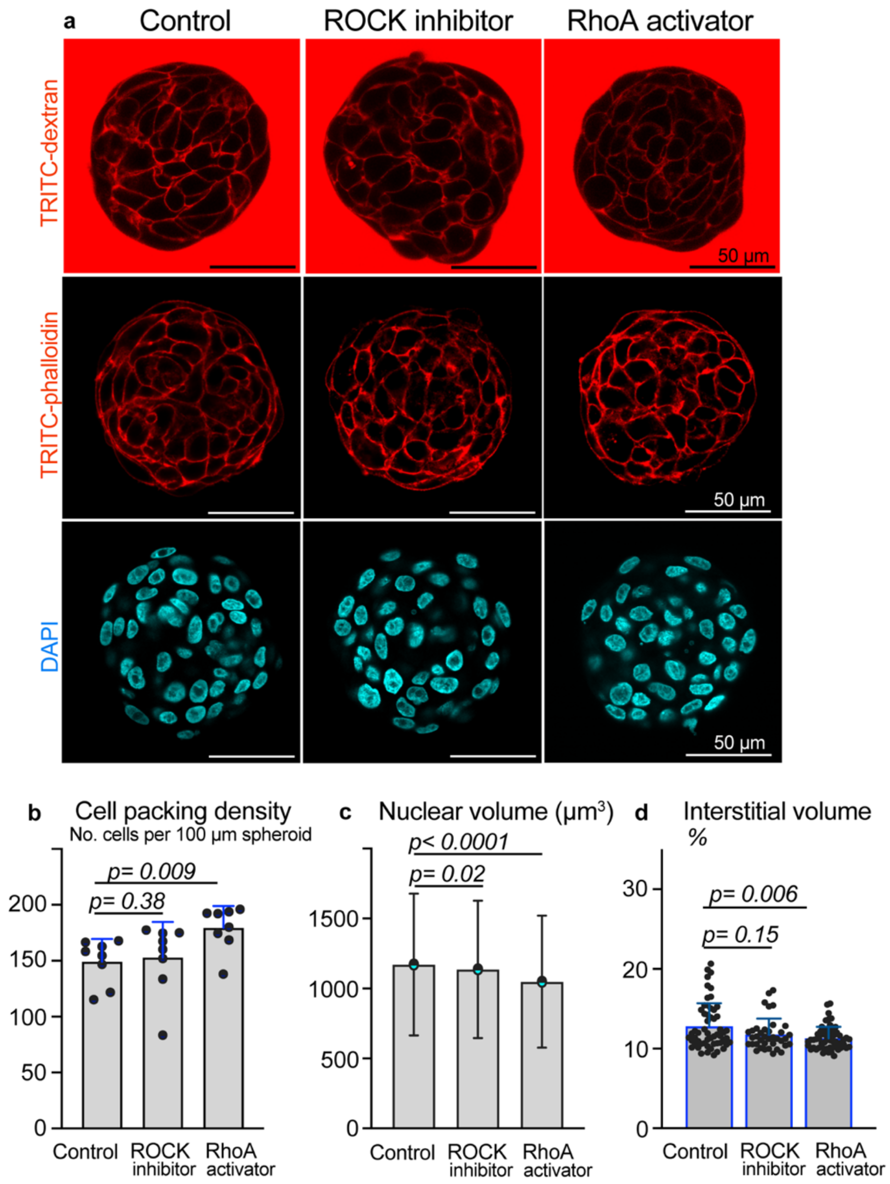

Figure S7. Spheroid packing density and interstitial spaces of MCF10.DCIS.com spheroids treated with a ROCK inhibitor or RhoA activator. a, Interstitial space visualization with TRITC-dextran in live MCF10.DCIS.com spheroids. Spheroids were placed in a solution of 2 g/L 3 kDa TRITC-dextran in media for 10-30 minutes. Confocal images were obtained using a 63x objective. Representative images of fixed spheroids immunostained with TRITC-phalloidin and DAPI are shown. **b-c:** 3D Arivis analysis was performed on live spheroids labeled with GFP-H2B. **b,** The cell packing densities are plotted as the number of cells per a typical spheroid volume of 530,000 μm^3 (representing a 100 µm diameter spheroid). 8 control, 8 ROCK-inhibitor and 8 RhoA activator spheroids were analyzed. Each dot represents a spheroid. **c,** The nuclear volumes of the cells in the spheroids are plotted. 8 control, 8 ROCK-inhibitor and 8 RhoA activator spheroids were analyzed. **c,** The interstitial volume are shown for the three conditions. V_I are determined by analyzing confocal z-stacks of a spheroid incubated with 2 g/L TRITC-dextran as described in the Methods. 5 control, 6 ROCK inhibitor and 6 RhoA activator treated spheroids were analyzed. Each dot represents a quantified z-plane within a spheriod. All spheroids were treated with ROCK inhibitor or RhoA activator for 24 h prior to imaging. Control spheroids did not receive any treatment. The mean and SD are shown. $p < 0.05$ are considered significant.

The cell packing density of RhoA activator- treated spheroids was slightly elevated; while the nuclear volume and interstitial spaces were reduced relative to controls. These factors could contribute to higher Brillouin shifts and linewidths observed in these spheroids.

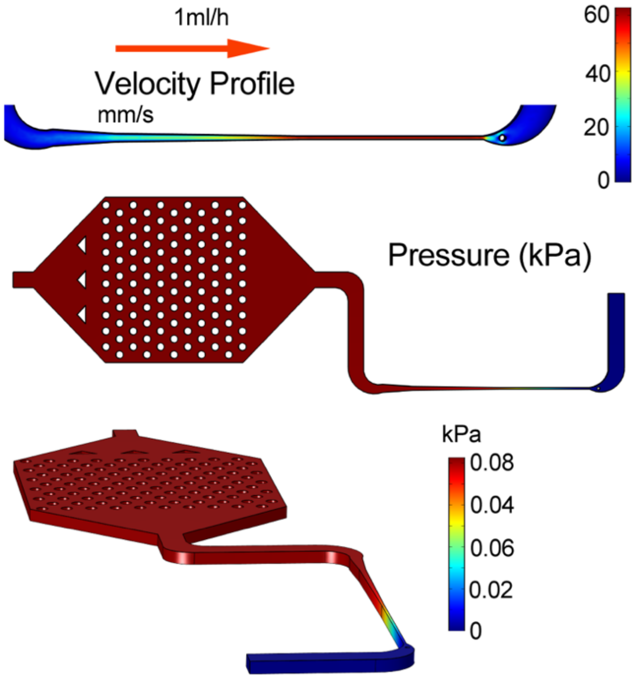

Figure S8. **Comsol modeling of the long-channel device.** Fluid modeling was performed considering a flow rate of 1 ml/h. Velocity and pressure profiles are shown. The long channel width gradually decreases from 200 µm- 40 µm, while maintaining a constant height of 190 µm. The total channel length is 5.25 mm. The channel with narrows in the following manner: 200-100 µm (750 µm long), 100-80 µm (750 µm long), 80-60 µm (750 µm long), 60-40 µm (750 µm long), 40 µm constant width (2.25 mm long). The spheroids stop when they reach the 40 µm- width zone and are imaged. Gradual reduction of the channel width is employed to facilitate the ability for the spheroids to stop in the channel. A single pillar is placed at the channel exit to also aid in spheroid slowing down and stopping in the compression channel.

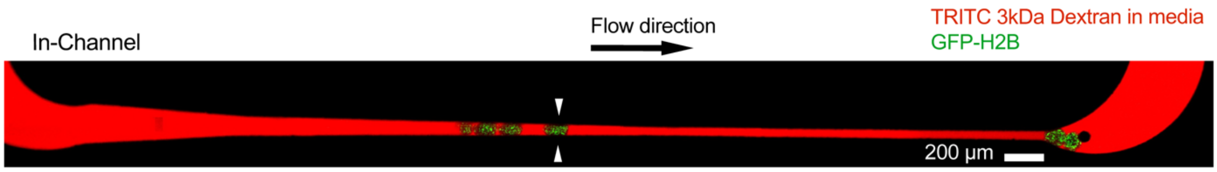

Figure S9. Device set up for measurement of dextran in compressed spheroids. Compressed spheroids were imaged in the 40 µm wide channel. The channel width decreases from 200- 40 µm along the direction of flow (white arrows indicate when the width reaches 40 µm). A single pillar at the exit aids in slowing down spheroids in the straight channel.

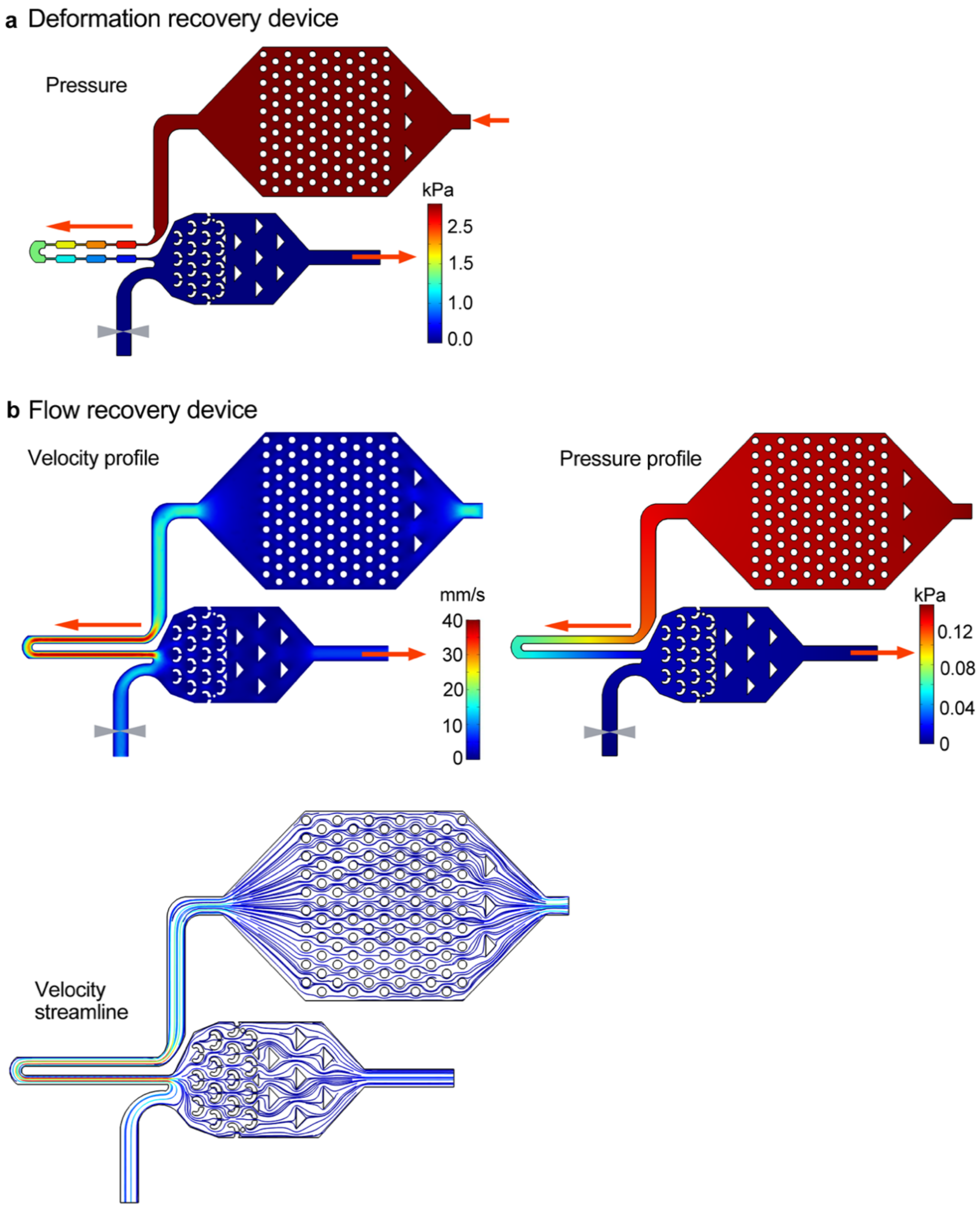

Figure S10. **Comsol modeling of deformation and flow recovery devices.** Fluid modeling was performed considering a flow rate of 3 ml/h. Pressure and velocity profiles are shown.

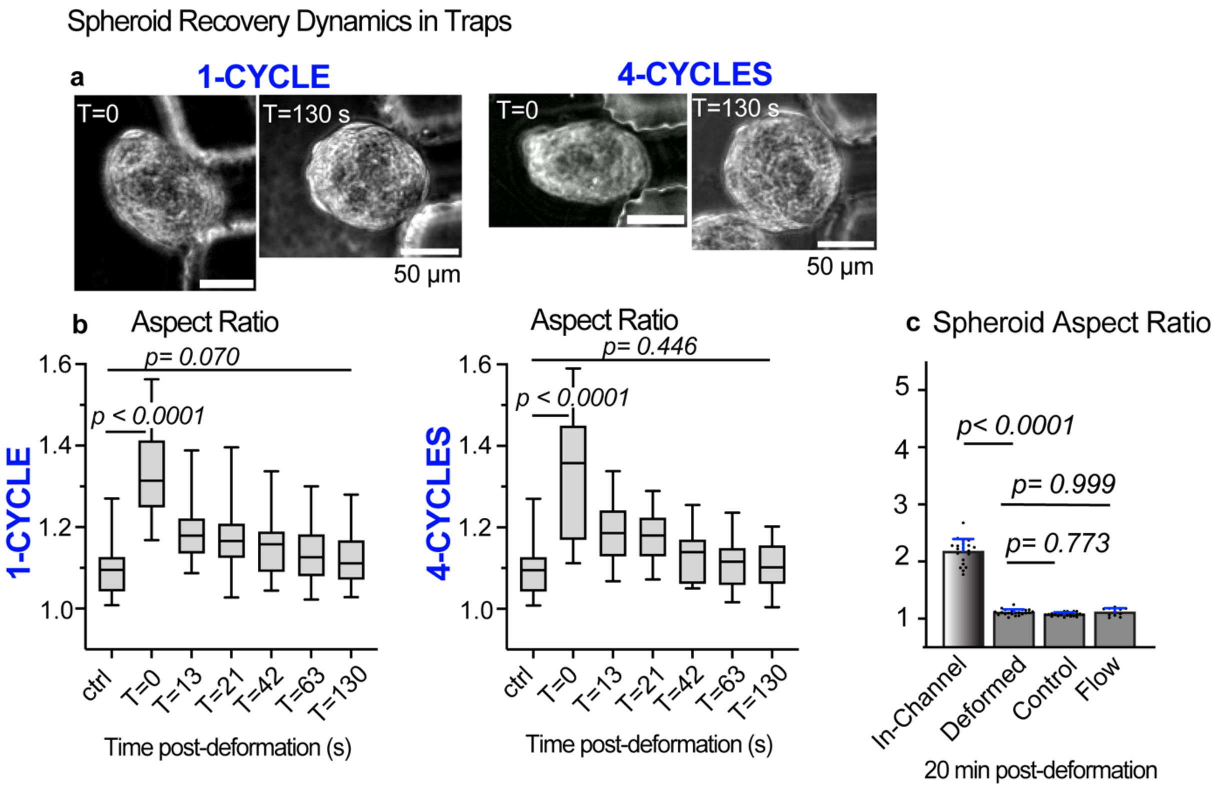

Figure S11. Spheroid recovery dynamics. a, Representative brightfield images of spheroids in recovery traps at different time points. **b**, MCF10.DCIS.com spheroids were introduced into the deformation recovery device at a constant flow rate of 3 ml/h suspended in media. Flow was stopped after spheroids were trapped. Shapes were recorded using timelapse imaging and tracing the spheroids at defined time points. Box and whisker plot is shown with the median ± min/ max. 1-cycle: N= 49 control spheroids and 21 recovering spheroids. 4-cycles: N= 49 control spheroids and 14 recovering spheroids. $p<0.05$ are considered significant. **c**, Spheroid aspect ratios. In-Channel represents live spheroids held in the compression channel, Deformed and Flow represent spheroids cycled through the respective devices 4 times and then fixed (20-min processing time). Control spheroids were kept at the same conditions but did not pass through the device. Mean + SD are shown. $p<0.05$ is considered significant. Dots represents spheroids. N= 21 in-channel, 22 deformed, 22 control and 11 flow spheroids.

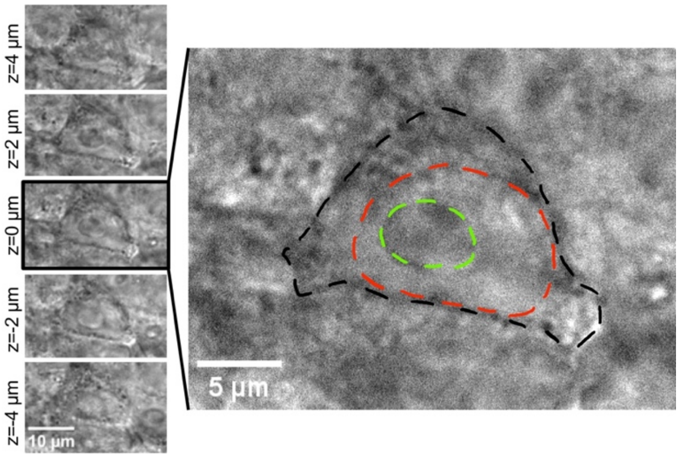

Figure S12. Brightfield images of MCF10.DCIS.com cells within a spheroids. Images were obtained using a 60x objective. The brightfield image ensures accurate focus within the cell. Left: A z-scan was performed to locate the cell. Images at different z-heights are illustrated and the selected plane is highlighted in black. Right: magnified view of the identified cell. The cell boundary is outlined in black, the nucleus boundary in red and the nucleolus in green.

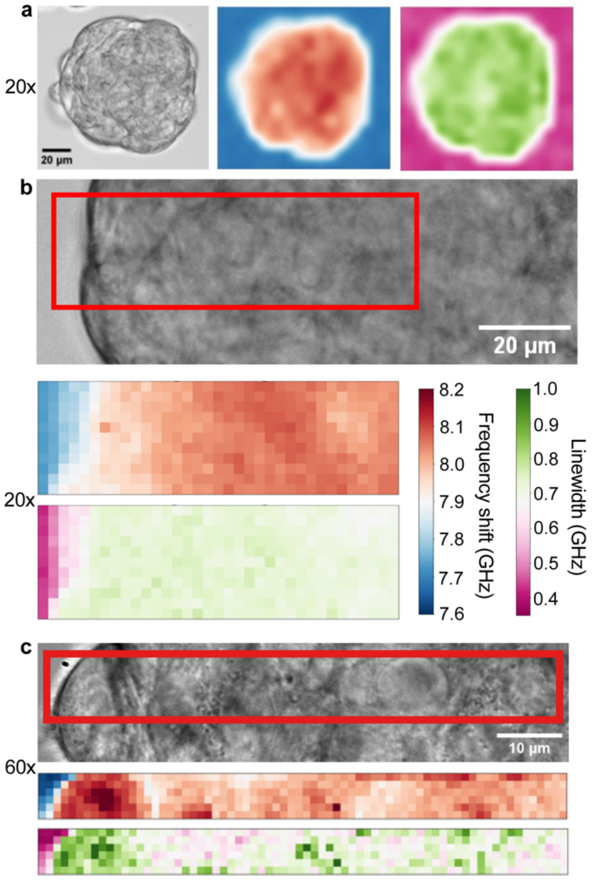

Figure S13: Brillouin frequency shifts and linewidth maps of MCF10.DCIS.com spheroids using 20x and 60x objectives. Spheroids were fixed with 4% PFA and suspended in PBS prior to imaging. **a,** Representative brightfield image of a spheroid. Brillouin shift map and linewidth map of the central plane of an entire spheroid are shown. The map was acquired with a low magnification 20x objective, step size: 7 µm and quadratic interpolation. **b,** Zoomed in brightfield image of the scanned region. Brillouin frequency shift and linewidth maps of a spheroid obtained with a low-magnification objective (20x), using a map step of 2 µm are shown. **c,** Brightfield image, Brillouin frequency-shift map, and linewidth map of a spheroid acquired with a high-magnification objective (60x), using a map step of 1 µm.

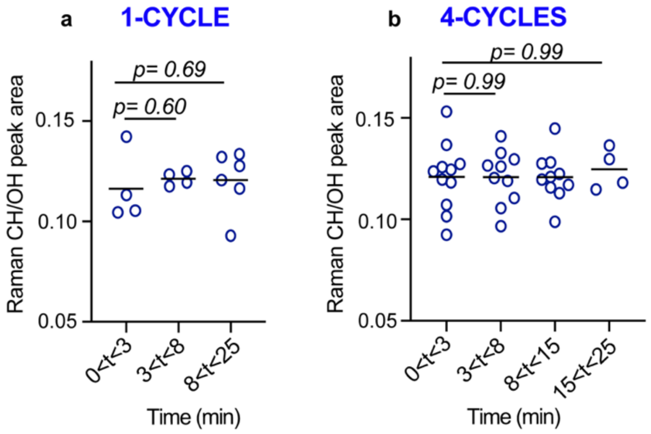

Figure S14. Raman analysis of 1-pass and 4-pass spheroids in the recovery traps over time. For 1-pass: N=4 spheroids for 0<t<3 minutes, 4 spheroids for 3<t<8 minutes and 6 spheroids for 8<t<25 minutes. For 4-pass: N= 11 spheroids for 0<t<3 minutes, 10 spheroids for 3<t<8 minutes, 10 spheroids for 8<t<15 minutes and 4 spheroids for 15<t<25 minutes. Mean values are shown. $p<0.05$ are considered significant.

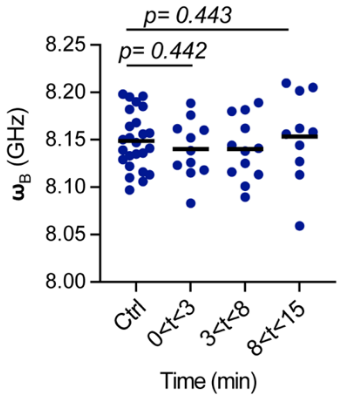

Figure S15. **Brillouin microscopy measurements for flow control spheroids.** Frequency shifts are shown for spheroids that passed through the flow recovery device. Mean values are shown, each dot represents a spheroid. N= 25 control spheroids, 11 spheroids for 0<t<3 minutes, 12 spheroids for 3<t<8 minutes and 10 spheroids for 8<t<15 minutes. $p<0.05$ are considered significant.

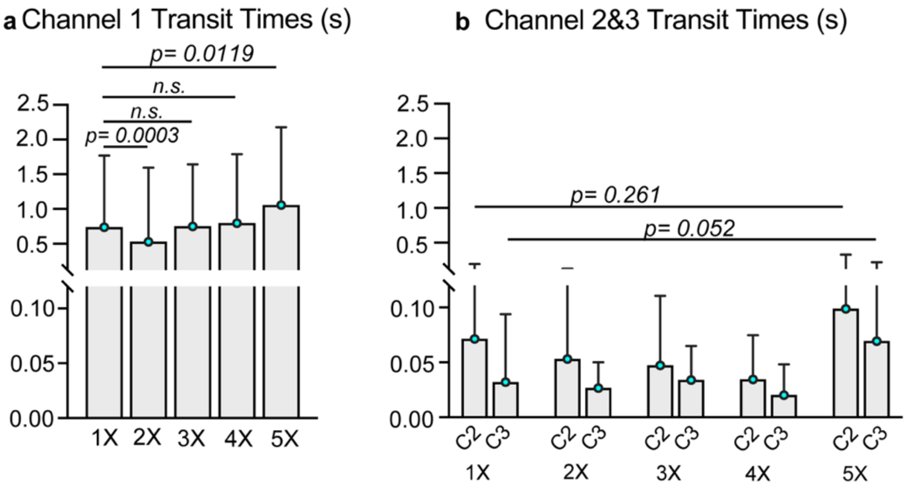

Figure S16. Spheroid transit times through constrictions. MCF10.DCIS.com spheroids are introduced into the deformation device at a constant flow rate of 3 ml/h suspended in media. Transit times through the constrictions channels are recorded using widefield microscopy and a frame rate of 167 frames/s: **a:** C1 and **b:** C2-C3. The first 3 channels are monitored using a 5X objective. Spheroids are cycled through the device 5 times, by re-introducing the spheroids back into the inlet tip. The time between cycling is 10 minutes. Mean + SD are shown. For channel 1, N= 127 spheroids for 1X, 55 spheroids for 2X, 59 spheroids for 3X, 84 spheroids for 4X and 63 spheroids for 5X. For channel 2, N= 126 spheroids for 1X, 55 spheroids for 2X, 57 spheroids for 3X, 83 spheroids for 4X and 62 spheroids for 5X. For channel 3, N= 126 spheroids for 1X, 55 spheroids for 2X, 55 spheroids for 3X, 81 spheroids for 4X and 61 spheroids for 5X. $p<0.05$ are considered significant.

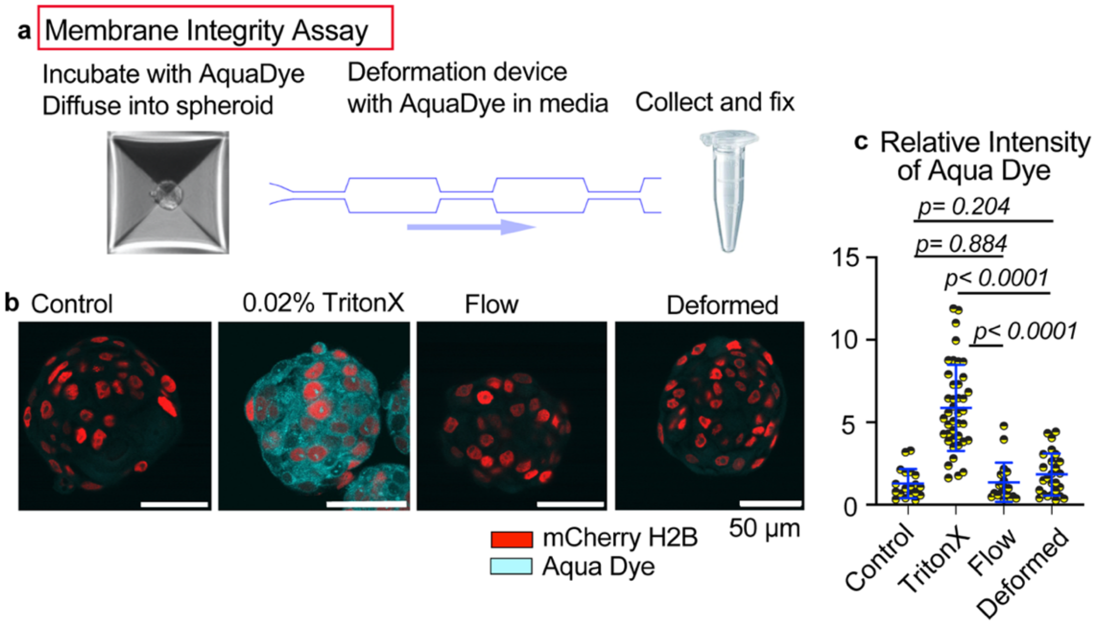

Figure S17. **Aqua dye membrane integrity assay**. **a**, Membrane integrity assay using aqua live cell dye. Schematic showing spheroids incubated with the live-cell dye for 2 h at 37ºC. Subsequently, spheroids were passed through the constriction device and immediately fixed, followed by imaging with a confocal microscope. **b,** Representative 63X images obtained with confocal microscope of spheroids incubated with the aqua dye. Control represents spheroids that did not pass through the device. Spheroids were pre-treated with 0.02% TritonX as a positive control. Flow and deformed represents spheroids that pass through the flow and deformation devices, respectively. Cells were stably transfected with fluorescent mCherry H2B. **c**, Relative intensity of the aqua dye was measured in the center of the spheroids, subtracting background signal. Spheroids passing through the flow or deformation device do not uptake the dye post-device as shown by low cellular dye intensities. Mean ± SD are shown. $p<0.05$ are considered significant. Dots represent spheroids. N= 19 control, 41 TritonX, 20 flow and 26 deformed spheroids.

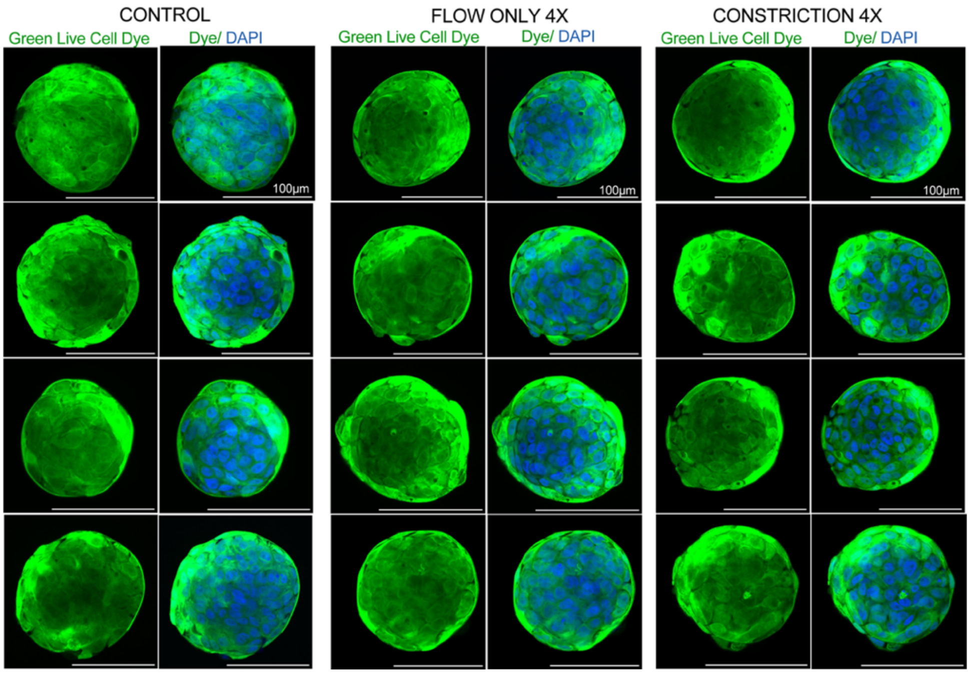

Figure S18. **Cytoplasmic live cell dye assay confirms viability 24h post- deformation.** The dye enters live cells (not permeablized), thus it doesn't diffuse uniformly into the spheroid center in all cases. After live cell dye incubation, spheroids were fixed with 4% paraformaldehyde, permeablized and incubated with DAPI for nuclear staining.

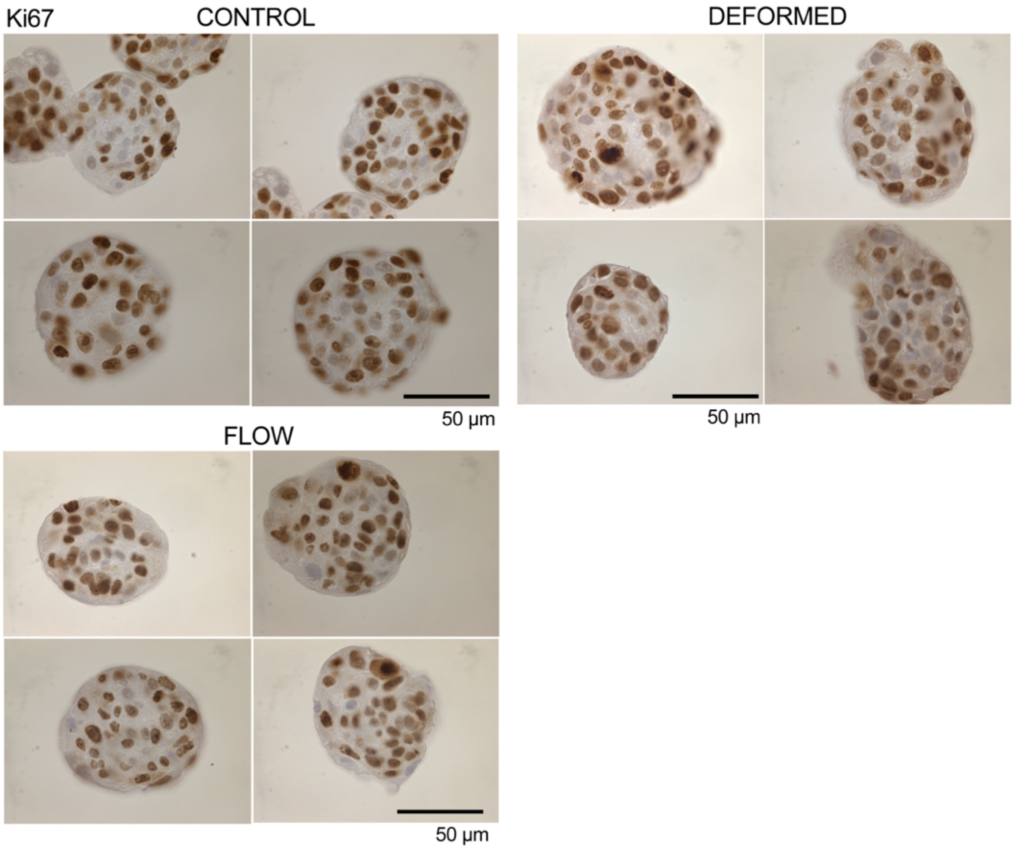

Figure S19. Ki67 immunohistochemistry of spheroids 24h post-deformation. Ki67 images were obtained with a widefield color camera and 60x objective. All sections were counterstained with Mayer's hematoxylin.

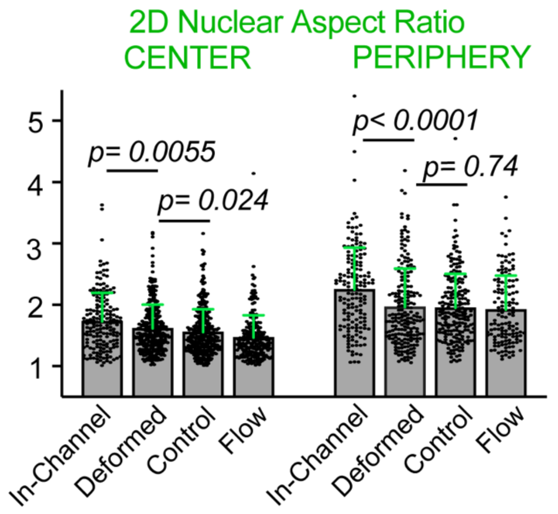

Figure S20. **Nuclear aspect ratios from the mid-plane of spheroids in-channel and post-chip**. Deformed and Flow represents spheroids that underwent 4x cycling and were fixed immediately following deformation or flow. Control spheroids were kept at the same conditions but did not pass through the device. In-Channel spheroids were imaged live within the fluidic channel. Mean + SD are shown. Dots represents nuclei. N (spheroids) = 19 in-channel, 13 deformed, 10 control and 9 flow spheroids. N (nuclei center) = 180 in-channel, 323 deformed, 328 control and 226 flow nuclei. N (nuclei periphery) = 159 in-channel, 206 deformed, 207 control and 137 flow nuclei. $p<0.05$ are considered significant.

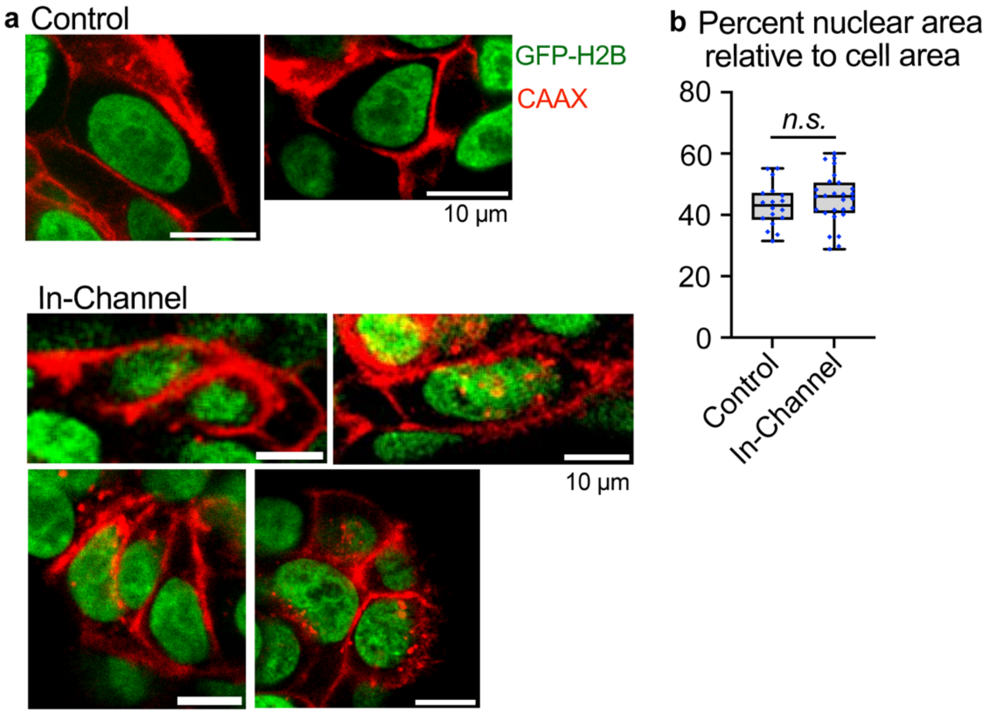

Figure S21. MCF10.DCIS.com nuclear and membrane labeled cells in spheroids. Confocal images showing GFP-H2B labeled nuclei and mCherry-CAAX membrane dye in control and compressed spheroids. Box and whisker plot is shown with the median ± min/ max. $p<0.05$ are considered significant. Dots represent a cell/nucleus. N= 3 control vs 3 in-channel spheroids (18 control cells vs 27 in-channel cells were analyzed). All scale bars are 10 µm.

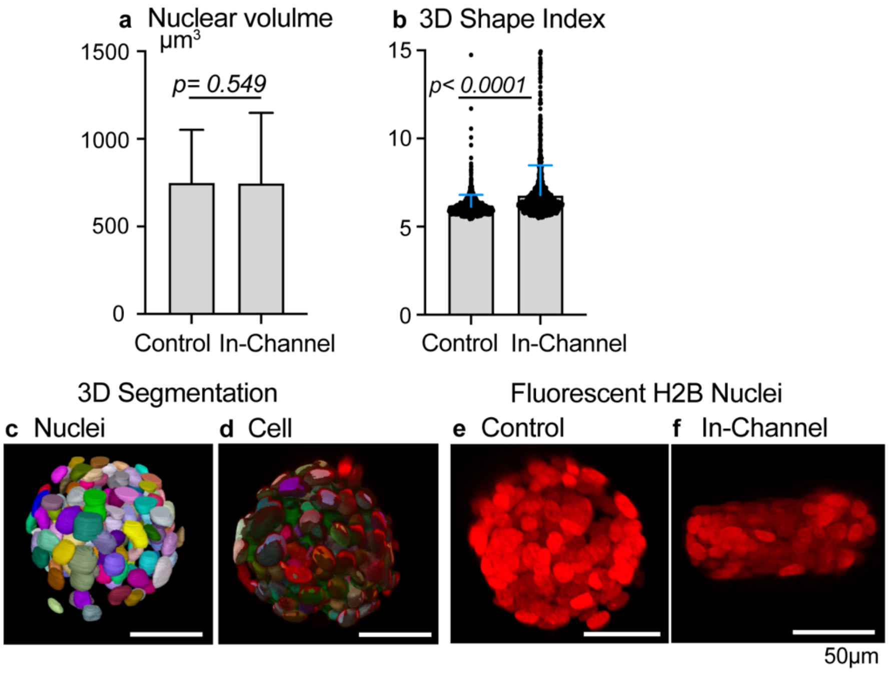

Figure S22. 3D segmentation of nuclei and cells in MCF10.DCIS.com spheroids. Cells were transfected with H2B-GFP for visualization of the nuclei. **a-b**, Nuclear volume and nuclear 3D shape index are plotted. 3D shape index = surface area/ volume$^{2/3}$. N= 11 control and 11 compressed spheroids. For (a) n= 1458 control nuclei and 1521 compressed nuclei were plotted. For (b) n= 1415 control nuclei and 1502 compressed nuclei were plotted. Mean + SD are shown. **c-d**, 3D segmentation images of nuclei and cells in the spheroid. **e-f**, Fluorescently labeled H2B nuclei in control and compressed spheroids.

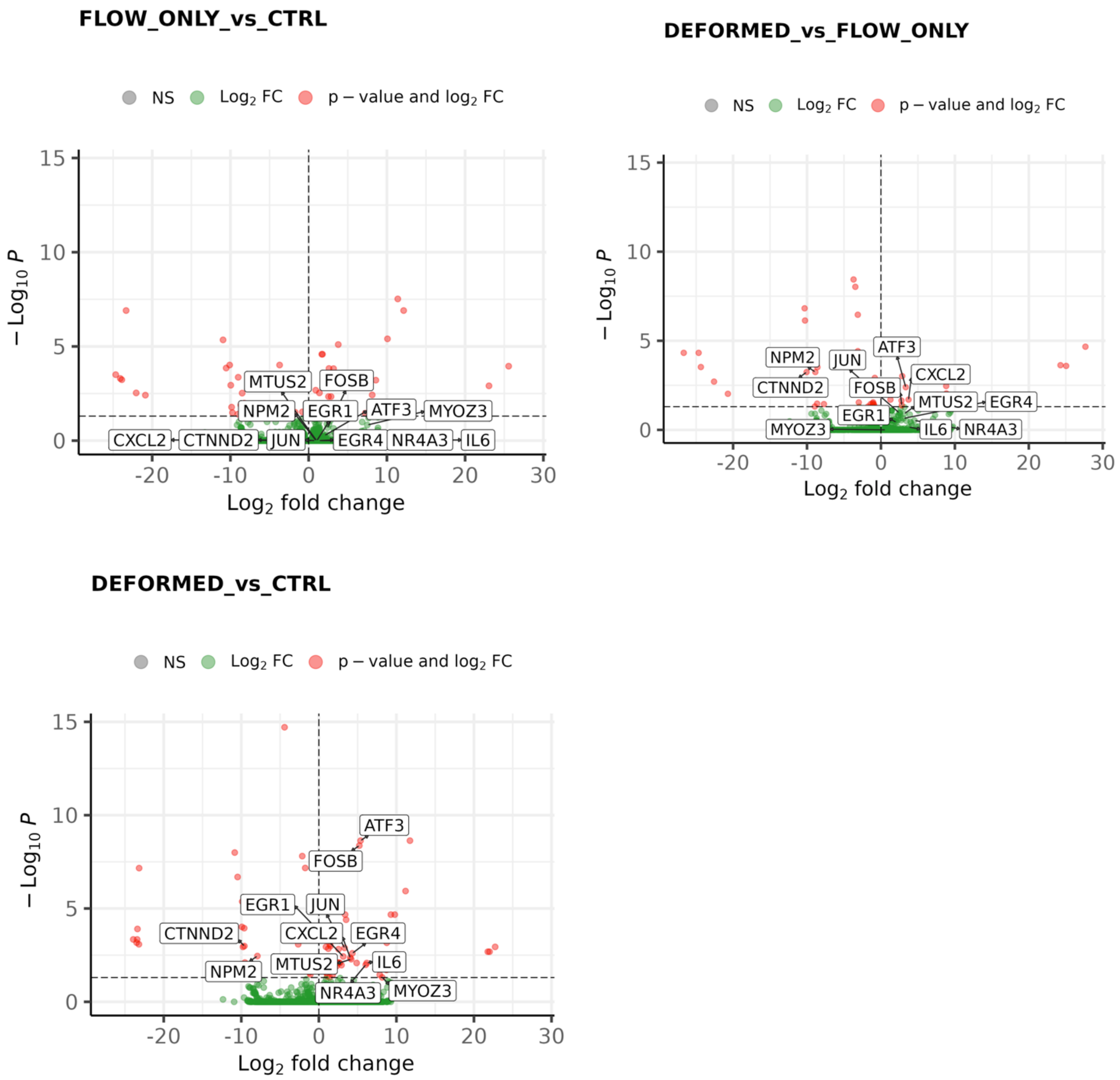

Figure S23. Volcano plots for MCF10.DCIS.com spheroids. Log_2 fold change (FC) are plotted in green. Log_2 fold change are plotted against the $-log_{10}P$ values (pink). Deformed and flow only spheroids represent spheroids that were cycled four times through the respective devices and then cultured for 1 hour in low attachment conditions. Control spheroids were not introduced into the devices but otherwise treated the same. N= 1500-3000 spheroids per condition.

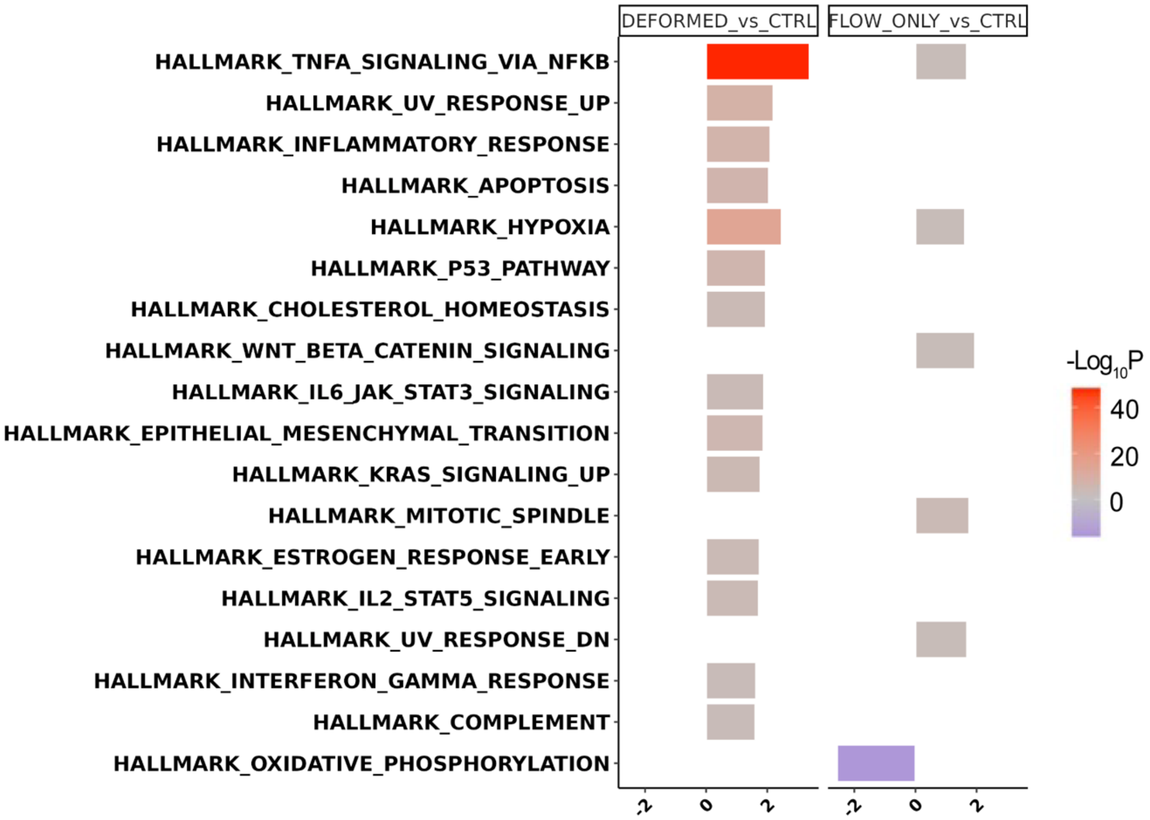

Figure S24. Hallmark pathways for MCF10.DCIS.com spheroids. Normalized enrichment scores (NES) from gene set enrichment analysis (GSEA) are shown. Deformed and flow only spheroids represent spheroids that were cycled four times through the respective devices and then cultured for 1 hour in low attachment conditions. Control spheroids were not introduced into the devices.

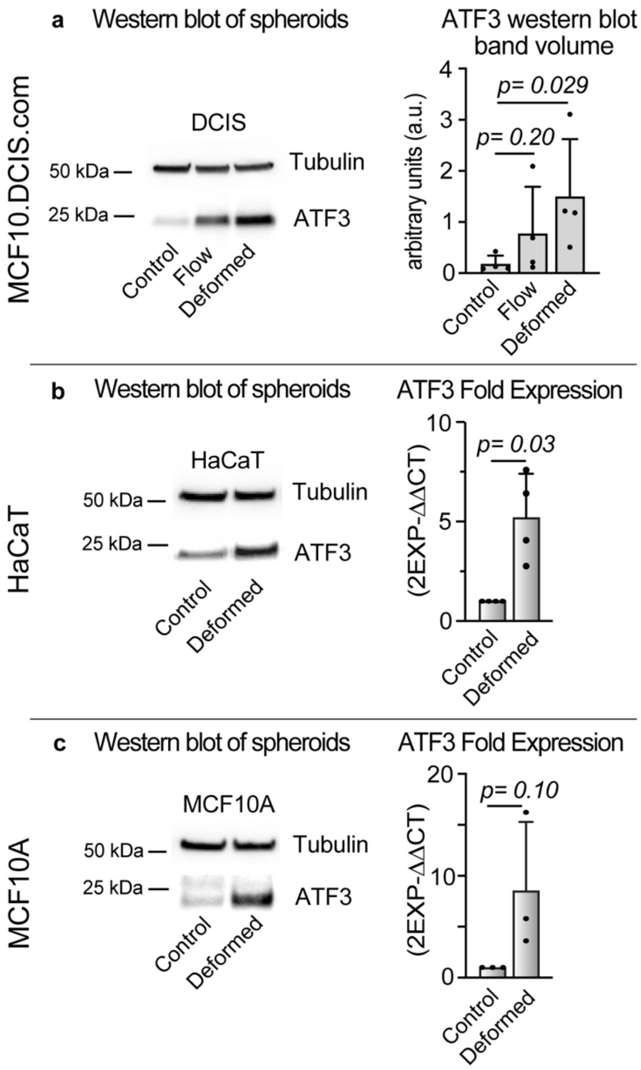

Figure S25. ATF3 levels increase following deformation of MCF10.DCIS.com, HaCaT and MCF10A spheroids. a, Protein levels of ATF3 in control, flow control and deformed MCF10.DCIS.com spheroids. Spheroids were cycled 4 times through the device and then cultured 4 hours post-chip. The ATF3 band volumes are calculated relative to tubulin volumes. The band volume consists of the sum of the pixel intensities x band area (pixels). Data are mean + SD. $p < 0.05$ are considered significant**.** Dots represent experiment repeats. N= 1500- 3000 spheroids per condition. **b-c,** Representative western blotting and quantitative PCR results showing relative ATF3 gene expression of HaCaT keratinocytes (**b**) and MCF10A (**c**) spheroids cycled through the deformation device 4 times. Spheroids used for western blot were cultured for 4 hours post-chip, while spheroids used for PCR were cultured for 1 hour post-chip. QRT-PCR values were normalized to the controls. Data are the mean + SD. Dots represent experiment repeats. N= 1500- 3000 spheroids per condition.

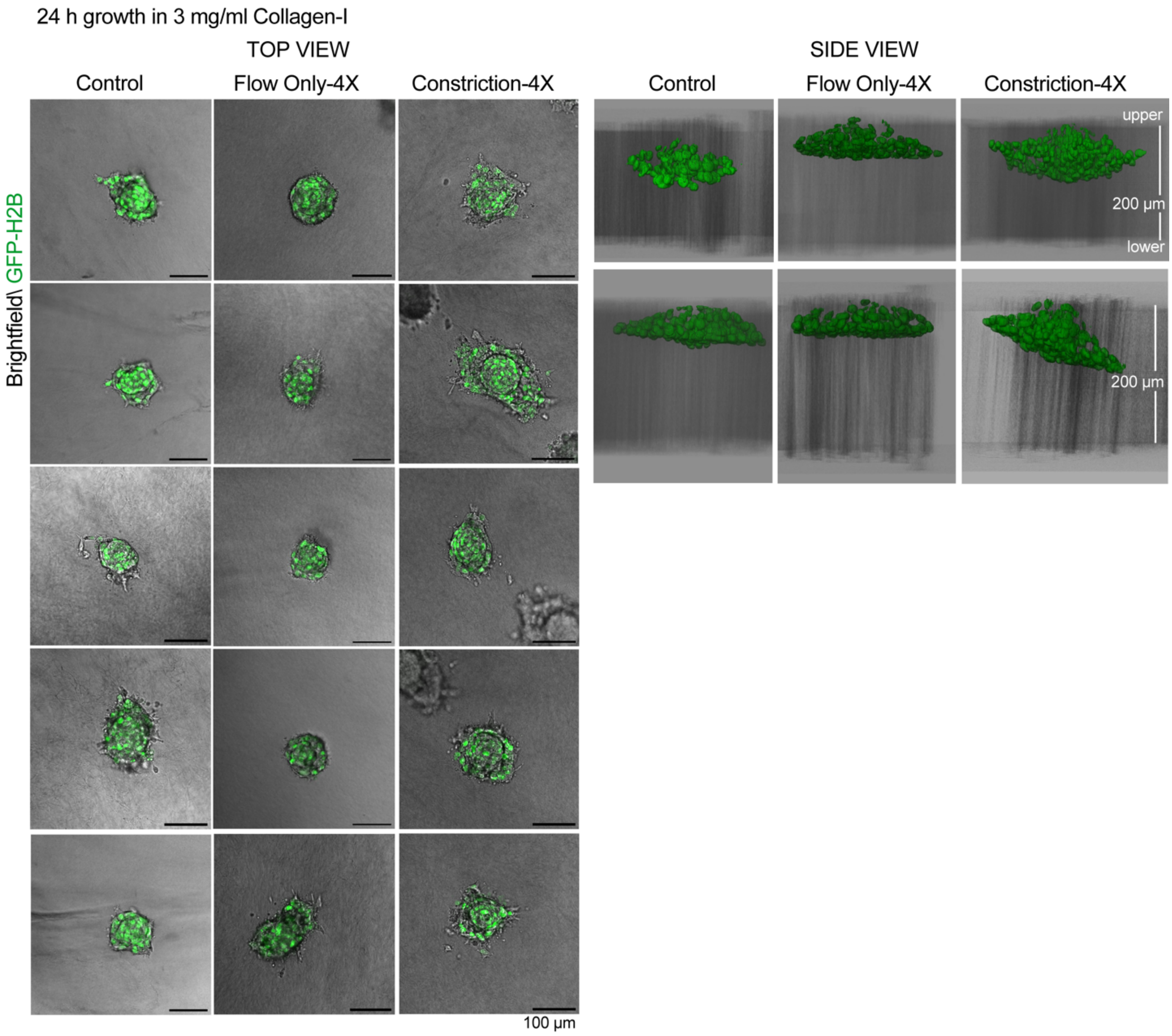

Figure S26. Panel images of collagen invasion of MCF10.DCIS.com spheroids. Control spheroids do not pass through the device, flow only and constricted spheroids are circulated 4 times through the respected devices and then embedded in the collagen matrix. Represented confocal images are shown of the spheroids after 24 hours of invasion in collagen I. The top view and side views are shown. For the side view, z-stacks were taken over 200 µm and 3D rendering was performed with Leica software. This view shows that the spheroids are embedded in the collagen and do not spread on the bottom of the well. The grey represents the collagen matrix and green is the fluorescent nuclei labeled with GFP-H2B. Images were obtained with a 25x water objective.

Experimental Section

Primary and secondary antibodies

- ATF3 antibody [clone EPR19488] - ChIP Grade Monoclonal antibody, Abcam Cat# ab207434 RRID:AB_2734728 (IF 1:100; WB 1:1000)
- Donkey Anti-Rabbit IgG (H+L) Polyclonal Antibody, Alexa Fluor 647 Conjugated, (Polyclonal, ThermoFisher Cat# A-31573, RRID:AB_2536183, multiple lots)
- Monoclonal Anti-α-Tubulin Clone B-5-1-2 mouse IgG1 isotype (Sigma-Aldrich Cat# T5168, RRID:AB_477579; WB 1:2000)
- Ki-67 Recombinant Rabbit Monoclonal Antibody (SP6) Thermoscientific, 1:50, MA5-14520

Live cell dyes

- Cell Tracking Dye Kit – Green. MW 500 Da. Abcam Cat# ab138891. (1:400 dilution for spheroid labeling)
- Dextran, Tetramethylrhodamine, 3000 MW, Anionic. Thermofisher Cat# D3307 (2g/L dilution for spheroid labeling)
- Live/Dead Fixable Aqua Dead Cell Stain kit, Cat. # L34957

QPCR primer identity
ATF3:Hs00231069_m1

Cell culture. MCF10A cells were cultured in DMEM/F12 media (Gibco) supplemented with 5% horse serum (Invitrogen), 2 mM L-glutamine (Euroclone), 1% penicillin-streptomycin (Sigma), 0.5 µg/ml hydrocortisone, 10 µg/ml insulin, 100 ng/ml cholera toxin and 20 ng/ml human epithelial growth factor (EGF, Invitrogen). Cells were grown in cell culture dishes 100 x 20 mm (CELL STAR®) at 37°C in humidified atmosphere with 5% CO_2. Hacat and vocal cord cancer cells (UT-SCC-11 stage 1 vocal fold cancer, and UT-SCC-103 stage 3 vocal fold cancer) were cultured in DMEM high glucose media (Invitrogen), 10% fetal bovine serum (Euroclone), 2 mM L-glutamine (Euroclone), 1% penicillin-streptomycin (Sigma), and 1% MEM non-essential amino acids (biowest). Spheroids were created in low attachment Aggrewell dishes (StemCell) and cultured for 24h.

Transfection for lentivirus infections. For lentivirus production, HEK-293T cells were transfected using the calcium phosphate method. In detail, for each transfection 5 µg or 10 µg of vector of interest was prepared in a tube with 5 µg pMDL-RRE (gag-pol), 2.5 µg of pRSV-REV, 2.8 µg pMD2G-ENV (VSV-G), 62.4 µL $CaCl_2$ 2M and 0.1X sterile TE buffer (pH 8) for a volume up to 500 µL. This solution was added dropwise to 500 µL 2X HBS while bubbling continuously. After 5 minutes of incubation at RT the mixture was added to the cells and cultured overnight at 37°C. Cells were incubated for 12-16 hours at 37°C and 5% CO_2. The day after transfection, the medium was removed, and 5.5 mL of fresh medium was added to each plate. 48 hours and 72 hours after transfection, the viral supernatant was collected and passed through a 0.45 µm filter. Supernatants were supplemented with polybrene 8 µg/mL and added to the target cells. Four cycles of infections were performed over two days.

Deformation Device experiment: (2 or 3- day experiment)

Day 1- Low- adhesion Aggrewell dishes (StemCell) were coated with a low adhesion solution by centrifugation at 1300 rcf, followed by a wash with MCF10.DCIS.com media. Single cells were seeded in 24-well Aggrewell dishes in triplicate, and centrifuged at 100 rcf, generating 1200 spheroids per well. 100-cells per micro-well were seeded (total 1.2 $x10^5$ cells per well), and cultured for 24 hours. Spheroid diameters after 24 hour of culture ranged from 85- 100 µm.

Day 2- Spheroids in the Aggrewell dishes were released from the micro-wells by gentle pipetting and PBS wash. Spheroids were centrifuged at 800 rpm, the media was removed, leaving 250 µl of spheroids in media remaining in a 1.5 ml Eppendorf tube. Hepes (25 mM) and gentamycin (0.2%) were added to the media. The control spheroids were left in the Aggrewell dish and kept at room temperature.

Devices were incubated with a sterilization solution (3% penicillin/ streptomycin and 0.6% gentamycin in PBS) flown into the microchannels for 20 minutes. Next, an anti-adhesion solution (StemCell) was introduced into the device for coating for 15 minutes, followed by a wash with media.

Flow- only devices and constriction devices were set up in parallel. Collection syringes were coated with 1% BSA in PBS prior to the experiment to prevent any non-specific sticking of spheroids inside the syringe.

Spheroids were introduced into the devices at a constant flow rate of 3 ml/h, and introducing 80 μl at a time, to prevent clogging in the chip and large pressure fluctuations (bursts of flow) which can damage the spheroids.

After the spheroids have passed through the device, a 50 μl media wash was performed. Next, the flow was stopped, and the spheroids in the collection syringe were re-introduced back into the inlet of the device for the next cycle. The syringe was washed with media and then discarded. A new syringe is used for each cycle. Spheroids were cycled through the same fluidic channel multiple times to mimic high frequency deformation events. The spheroids did not accumulate in the channels between passes, as was confirmed with manual visualization under a microscope.

After the final pass, the spheroids can be immediately collected for analysis, or cultured for 24 hours.

In the case of immediate collection, the collection solution in the syringe is gently ejected into a 1.5 ml Eppendorf tube. The spheroids were then used for various downstream assays.

Spheroids cultured for 24 hours were ejected into a 6-well low attachment well. Spheroids were cultured in 1.5 ml of media in the 6-well low attachment dishes (Corning) together with 20 ng/ml EGF at 37°C and 5% CO_2 together with the control. The low volume of media is necessary as the spheroids remain spaced apart along the border of the well, which prevents spheroid fusing. Following culture, the spheroids were collected, centrifuged to remove the media and wash with PBS, then used for live-cell assay, PCR, western blot, or immunofluorescence staining.

Device design and fluid modeling.

The device designs were performed using AutoCAD software (LT 2021), and polyester of chromium masks were used for soft lithography protocol (details are in the Supporting Information). To ensure the optimal performance of our microfluidic device, we conducted simulations that provide an initial validation of the design and predict the fluid dynamics encountered by our samples. These simulations were performed using COMSOL Multiphysics®, a finite element analysis software, which allows for the creation of a detailed computational mesh that accurately represents the geometric features of the microfluidic device. The mesh was generated from a 3D design imported from AutoCAD, where the initial design was created and extruded before being transferred into Comsol for further analysis. By imposing a constant flow rate and solving the Navier-Stokes equations, Comsol generates velocity profiles and pressure distributions throughout the device. These simulations are crucial for establishing the correct design parameters, as they validate the device's ability to generate physiologically relevant pressure gradients, similar to those experienced by tumor cells *in vivo*.

Simulation Workflow in Comsol

1. **Geometric Modelling**:
   - The geometry of the microfluidic device was initially designed and extruded into a 3D model using AutoCAD and setting a height of 190 μm. This design, including all essential features such as channels, inlets, and outlets, was then imported into Comsol for further processing.
   - Once imported, the geometry was reviewed and adjusted within Comsol to ensure compatibility with the simulation requirements, preserving the accuracy and scale necessary for the intended analysis.
2. **Material and Fluid Properties**:
   - Relevant material properties, such as the fluid's viscosity and density, were assigned based on literature values and experimental data (medium density=1.009 g/cm^3, medium dynamic viscosity=0.94 cP)[2].

    - The fluid was modelled as an incompressible Newtonian fluid and laminar flow, which is appropriate for the biological conditions being simulated.
3. **Boundary Conditions**:
    - **Inlet and Outlet**: A constant flow rate was applied at the inlet, while the outlet was set to a fixed pressure with a suppress of backflow.
    - **Walls**: No-slip boundary conditions were applied to the walls of the channels, assuming that the velocity of the fluid at the wall is equal to the velocity of the wall itself.
4. **Meshing and Mesh Convergence**:
    - A computational mesh was generated, with finer mesh elements near regions of interest, to capture the detailed flow dynamics.
    - Mesh convergence studies were performed to ensure that the simulation results were independent of mesh density, achieving a balance between accuracy and computational efficiency.
5. **Physical Model Setup**:
    - The Navier-Stokes equations were solved under steady-state conditions initially.
    - Following the steady-state analysis, a time-dependent study was conducted for particle tracking. This step allowed us to analyse the trajectories and distribution of particles within the device over time, providing insights into how the microfluidic environment influences sample behaviour.
6. **Simulation Execution**:
    - Simulations were run until convergence criteria were met, ensuring that the results were stable and reliable.
    - The outputs, including velocity profiles, pressure distributions, and particle trajectories, were then analysed to validate the design and predict the device's performance under experimental conditions.

Device fabrication - soft lithography. The device is made using soft lithography. Silicon wafers (Prime CZ-Si wafer 4 inch, thickness = 525 ± 25 µm, Microchemicals) were dried using an air gun and then cleaned with a plasma cleaner (PVA TEPLA 300 AL) for 5 min. 4 mL of SU8-3050 was added on the surface of the silicon wafer (mirror side up) by weighing 4.61 g (SU8-3050 density 1.219 g mL^{-1}).

A layer of 190 µm of SU-3050 was obtained setting the spin coater (POLOS) with the following program: step 1 - 500 rpm for 5 s with acceleration of 100 rpm s^{-1} and step 2 - 900 rpm for 0.8 s with acceleration of 500 rpm s^{-1} followed by 900 rpm for 30 s and a final step 3- of 1900 rpm for 1 s at 1000 rpm s^{-1}. Then the SU8-wafer was soft-baked at 65°C for 2 min and then 95°C for 45-50 min, and cooled at RT. Then a chromium mask (Chrome Mask on 5" Quartz 5 x 5 x 0.9 inch, Pattern Side Down, clear polygon tone, PhotomaskPORTAL) with the desired pattern was placed into a Mask Aligner (Karl Süss MA6/BA8) on top of the silicon wafer (soft contact gap of 31, exposure energy of 500 mJ/cm^2) where it was exposed to UV light to cross-link the exposed regions in the mask. A post-UV exposure bake (PEB) was performed at 95°C for 5 min. All temperatures were obtained by gradually ramping the temperature from room temperature to the set point. The non-crosslinked SU8 was eliminated due to 20 min of agitation with an SU8 developer solution. The master was washed with isopropanol (IPA) and left to dry at RT. Then the microfluidic devices were fabricated using molds and elastomer polydimethylsiloxane (PDMS, Dow Corning SYLGARD™ 184 Silicone Elastomer Kit 1.1 KG KIT, Dow Corporate).

PDMS device fabrication. PDMS (1:10 ratio of curing agent: PDMS) was poured ontop of the master and baked at 80°C for 45 minutes, then removed from the oven and cooled to RT. The cured replicas were then peeled off, punched, and plasma bonded to thickness no. 1 glass coverslips (Prestige®) that were pre-coated with a thin layer of PDMS. The bonded chips were kept at 80°C for 20 min to secure a robust bonding. Afterwards, the silicon tubing was attached to the inlet and outlet of the devices. Before use, the devices were conditioned with 5% Pluronic F68 (Sigma-Aldrich) in PBS for 24 hours to reduce the non-specific adsorption.

RNA extraction:
Following the experiment, the spheroids were collected, washed with PBS and the pellet was frozen at -80°C. The spheroids were frozen in a small amount of PBS (5- 10 µl). Briefly, the Qiagen Micro-Kit was applied, adding 10 µl of B-mercaptoethanol into 100 µl of RLT buffer, followed by vortexing for 15 seconds, homogenizing lysed spheroids with 1 ml syringes (5- passes). The lysate was pipetted onto the QIA shredder column and spun at 14,000 rpm. An equal volume of 70% ethanol was added to the collected flow through, and applied into the RNAeasy micro- column, centrifuged for 14,000 rpm. The wash buffer RW1 was added onto of the micro-column and centrifuged. Next, 80µl of DNAase solution is added onto the RNAeasy micro-column and incubated for 15 minutes, followed by a second wash with RW1, wash with RPE buffer, and last an 80% ethanol solution. RNA was eluded with 12 µl of RNA-ase free water and quantified with NanoDrop 2000 Spectrophotometer. Quantities obtained for microfluidic spheroid experiments were 0.45- 3 ug of RNA.

Quantitative RT-PCR. Total RNA from cells was extracted using RNeasy Plus Mini Kit (Qiagen) and quantified by NanoDrop 2000 Spectrophotometer to assess both concentration and quality of the samples. Reverse transcription to generate cDNA was performed using the High-Capacity cDNA Reverse Transcription Kits (Applied biosystems) and PCR thermal cycler (10 min at 25°C, 2 hours at 37°C, 5 min at 85°C and ∞ at 4°C). 500 ng of RNA was reverse transcribed into cDNA. 5 ng of cDNA was amplified, in triplicate, in a reaction volume of 10 µL containing the following reagents: 5 µL of TaqMan® Fast Advanced Master Mix (Thermofisher), 0.5 µL of TaqMan Gene expression assay 20x (Thermofisher). Real-time PCR was carried out on the 7500 Real-Time PCR System (Thermofisher), using a pre-PCR step of 20 s at 95°C, followed by 40 cycles of 1 s at 95°C and 20 s at 60°C. Then, samples were amplified with primers for each target and for all the targets one no-template-control (NTC) sample was run. Reactions with Ct values greater than 35 were determined to be below the limit of detection. GAPDH was used as the housekeeping gene and the cycle threshold (Ct) of genes detected were normalized to this gene. qPCR was performed by the qPCR-service at Cogentech, Milan. The relative gene expressions were determined from the 2EXP-ΔΔCT method and the log2 expression of 2EXP-ΔΔCT.

Western blot. Protein samples were prepared by lysing cells in 1X JS buffer containing protease inhibitor cocktail to prevent the degradation of proteins. Cells were incubated on ice for 15 min then lysates were centrifuged at 14,000 rcf for 20 min at 4°C to remove the debris. The cleared lysates were transferred to new vials and the protein concentration of each sample was measured by Bradford colorimetric protein assay following manufacturer's instructions. Equal amounts of the protein (20-50 µg per lane) were prepared adding 5X LaemmLi and lysis buffer to make up the volume of 40 µL. Proteins were denatured through a 10 min incubation step at 95°C. Then, samples were loaded on 4-12% precast polyacrylamide gels (BOLT TM 4-12% Bis-Tris Plus Invitrogen) alongside a protein marker (Novex). Electrophoresis was performed (200 V for 30 minutes) to separate the proteins of different molecular weights. Proteins were transferred from the gel to nitrocellulose membrane (GE Healthcare Life science) in western transfer buffer 1X (diluted with isopropanol) at constant voltage (100 V for 1 hour). Ponceau coloring followed by TBS 0.1% Tween (TBS-T) wash were used to reveal the amount of transferred proteins. The membranes were cut in two parts, one to reveal the protein of interest and one to reveal the house keeping protein. Membranes were blocked for non-specific antibody binding using 5% skimmed milk in TBS-T for 1 hour (blocking solution). Blocked membranes were incubated in blocking solution with primary antibody specific to the target protein for one hour at room temperature. Three washes of 5 min each was performed with TBS-T. The protein of interest membrane was incubated with anti-rabbit HRP conjugated secondary antibody while the house keeping protein membrane was incubated with anti-mouse HRP conjugated secondary antibody, both for one hour at room temperature. Three washes of 10 min each was performed again with TBS-T. The membranes were then developed using Thermo Scientific™ SuperSignal™ West Atto Ultimate Sensitivity Substrate and imaged using ChemiDocTM XRS+ System (Bio-Rad).
Western blot volume measurements from the ATF3 bands were determined using the iBright™ Imager Analysis Report which represents the integrated signal intensity of the bands in arbitrary units (A.U.) and the band area. The band volume is the sum of all the pixel intensities (brightness or darkness values) within a defined region of interest (ROI) that the software selected around each band. Band Volume= Sum of Pixel Intensities ×Band Area (Pixels).

The data are shown below:

Table S1: iBright western blot volume measurements

Date	sample	ATF3 (A.U.)	Tubulin (A.U.)	Normalized values
16.9.24	Control	4784680	11320700	0.42264878
	Flow	20073100	9602720	2.09035565
	Deformed	31794900	10236900	3.10591097
24.10.24	Control	1151040	13343400	0.08626287
	flow	9037480	13088100	0.69051123
	Deformed	12902300	10933500	1.18007043
2.12.24	Control	1288900	10122000	0.12733649
	Flow	2044260	9552790	0.21399612
	Deformed	4593660	9024640	0.5090131
3.12.24	Control	1013220	10860000	0.09329834
	Flow	973735	8424560	0.11558289
	Deformed	10655000	8774900	1.21425885

Collagen Invasion Assay. Invasion assays were performed in Ibidi 8-well dishes. Collagen mixtures were prepared on ice in a sterile biosafety cabinet. Initially, the base of the Ibidi chambers were coated with 60 µl of 3 mg/ml rat tail collagen I (Corning) and incubated at 37ºC for 10 minutes to allow the base layer to form a gel. Next, spheroids were pipetted into 96-well u-bottom dishes (Corning) to confirm the introduction of at least 10 spheroids in 100 µl volume. 3 mg/ml rat tail collagen I was prepared together with the spheroids and gently pipetted into the Ibidi chamber, on top of the bottom coat. The pH of the collagen mixture is confirmed using pH strips (Cytiva) as 7.0- 7.4. The collagen mixture is shown below, where quantities are prepared for 3 Ibidi wells.

Preparation of coating layer for 3 wells:

Table S2: Collagen Reagent Amounts

**Reagent (stock concentration)**	**Working conc.**	**Volume (ul) allow for coating**
DMEM media		189.6
$NaHCO_3$ (7.5% v/v)	0.75%	40
Hepes 1 M	50 mM	20
Collagen I (8.08 mg/ml)	3 mg/ml	148.5
NaOH (1M)	5 mM	2
	Total (ul)	*400*

Preparation of collagen mixture containing spheroids:

**Reagent (stock concentration)**	**Working conc.**	**Volume (ul) allow for coating.**
DMEM media		174
$NaHCO_3$ (7.5% v/v)	0.75%	100
Hepes 1 M	50 mM	50
Collagen I (8.08 mg/ml)	3 mg/ml	371.3
NaOH (1M)	5 mM	5
	Total (ul)	*700*
	Divide into 3 tubes	*233*
	Add spheroids suspended in media	*100*
	Total (ul)	*333*

The lid of the Ibidi chamber was placed ontop and the system was placed in the incubator. After 1 hour to allow the collagen to gel, an additional 176 µl of media was added ontop of each well. 20 ng/ml EGF was included in the media. Expansion and invasion into the collagen matrix were monitored after 24 hours using a 20X objective with a confocal laser scanning microscope (CLSM). Confocal z-stacks were performed with a 25X water immersion objective.

Quantification of invasion: Quantification of invasion events was performed using Fiji imaging software. The invasion and areas were obtained by manually tracing the invasive front using the brightfield channel. The core was also traced manually. A total of 36 control spheroids, 61 flow- only and 69 constricted spheroids were quantified in 4 independent experiments.

Immunofluorescence of spheroids. Spheroids were collected in an Eppendorf tube and suspended in PBS, then centrifuged at 800 rpm, PBS was removed and fixed with 4% PFA for 30 min, rinsed with PBS, permeabilized with 0.2% Triton X-100 for 3 h, and washed with IF buffer (0.2% TritonX, 0.1% BSA, 0.05% Tween20 in PBS). Spheroids were blocked for non-specific binding sites in 5% BSA for 1.5 h. Primary antibodies were diluted in IF buffer and added for 48 h incubation at 4°C. Then, the spheroids were washed with PBS before secondary antibody incubation for 24 h at 4°C. Secondary antibodies were diluted in IF buffer. A wash with IF buffer for 5 minutes was performed to remove the excess of secondary antibodies and the nuclei of cells were stained with DAPI (1:1000 dilution) for 2 h at RT. Spheroids were washed with PBS and mounted between glass slides as described in the 'Mounting Spheroids for Confocal Imaging' section. Cells and clusters were imaged with a 63X objective with a confocal laser scanning microscope (CLSM) Leica TCS SP8 mounted on an inverted microscope Leica DMi 8 (LASX plus NAVIGATOR software).

Mounting Spheroids for Confocal Imaging

120 µm- high circular spacers were placed onto a coverslip (25 x 75 mm and 0.17 mm thick: Logitech). Fixed spheroids in PBS were pipetted onto the spacer in a 10 µl drop (containing 10- 50 spheroids). The PBS was removed using kimwipes and then a drop of 10 µl of glycerol was added ontop of the mostly PBS-free spheroids. A circular coverslip (Prestige, 2.5 mm diameter, 0.17 mm thick) is placed ontop of the spacer and the edges are sealed with nail polish. Spheroids are imaged using a 63X oil objective with a confocal laser scanning microscope (CLSM) Leica TCS SP8 mounted on an inverted microscope Leica DMi 8.

Confocal Imaging of Spheroids in Constriction Channels. MCF10.DCIS.com spheroids were introduced into the long- channel device at 1 ml/h in culture media containing 25 mM hepes. The flow was halted when the spheroid was trapped in the constriction channel 40 µm-width zone. Spheroids were imaged with a 60X water objective with iXplore Olympus spinning disc confocal microscope (CSU-W1 confocal scanning unit Yokogawa with a controlled temperature incubator (37°C), and 0.4 µm step size.

Immunohistochemistry

Samples are processed by a Diapath automatic processor as follow. Tissues were dehydrated through 70% (60 minutes), 2 change of 95% (90 minutes each) , and 3 change of 99% (60 minutes each) ethanol, cleared through 3 changes of xylene (90 minutes each), and finally immersed in 3 changes of paraffin, 1 hour each. Samples were embedded in a paraffin block and store at room temperature until ready to section. According to standard protocol, Haematoxylin/Eosin (Diapath) were performed.
For IHC analysis paraffin was removed with xylene and the sections were rehydrated in graded alcohol. Antigen retrieval was carried out using preheated target retrieval solution (pH6) for 30 minutes and endogenous peroxidase activity was quenched with 3% hydrogen peroxide in distilled water for 10 minutes at RT. Sections were blocked with FBS serum in PBS for 60 min and incubated overnight with primary anti Ki67 (Thermoscientific, 1:50, MA5-14520). The antibody binding was detected using a polymer detection kit (GAR-HRP, Microtech) followed by a diaminobenzidine chromogen reaction (Peroxidase substrate kit, DAB, SK-4100;Vector Lab). All sections were counterstained with Mayer's hematoxylin. Slides were imaged with a widefield microscope (Olympus Upright BX61).

Membrane Integrity Assay
MCF10.DCIS.com spheroids were incubated with the live cell dye (1:250) for 2 hours prior to the microfluidic device. The spheroids passed through the microfluidic channels in the presence of Aqua live cell dye (ThermoFisher). The spheroids were collected post- chip, and fixed with 4% paraformaldehyde and mounted on a glass slide in glycerol mounting medium. Control represents spheroids that were not introduced into the device. Spheroids were treated with 0.02% TritonX as a positive control for a compromised membrane; treatment occurred for 30 minutes prior to the introduction of the dye; spheroids remained in media with the TritonX for the duration of the dye incubation (2 h), after which they were collected and fixed. Spheroids were imaged with 63X objective confocal microscope to obtain the central z-plane of the spheroid. The relative intensity of the aqua dye was measured by tracing the spheroid area, and subtracting the background signal.

Spheroid viability assay. Live spheroids were incubated with fluorescent green cytoplasmic dye (abcam; dilution 1:400) for 2 hours at RT, then washed with PBS, fixed with 4% paraformaldehyde for 20 min at RT, washed with PBS, permeabilized with 0.2% TritonX 100, washed with PBS and then incubated with DAPI (1:1000) for 2 hours at RT and washed with PBS. Spheroids were then mounted between glass slides in glycerol as described previously.

Spheroid recovery dynamics.
MCF10.DCIS.com spheroids were introduced into the deformation recovery device at 3 ml/h and timelapse videos were obtained as soon as the spheroids reached the recovery traps. 1-cycle spheroids were introduced directly into the recovery device. 4-cycle spheroids were cycled 3 times through the deformation device, and then the final 4th time through the deformation recovery device.
Videos were obtained using an iXplore Olympus spinning disc confocal microscope (CSU-W1 confocal scanning unit Yokogawa) with 10x objective and 4.2 s time step for a duration of 3 minutes. Spheroids were imaged using brightfield and 488 channels. The brightfield channel was used for tracking the spheroid shape changes over time. The 488 channel was used to image the fluorescent GFP-H2B nuclei, to confirm whether the spheroid rotated during the timelapse imaging. Spheroids that rotated were excluded from the shape analysis. Using Fiji, the spheroid areas were manually traced at defined time points and shape parameters were obtained.

Automated nuclei analysis
We employed a python-based script with Fiji to identify spheroid border from high resolution confocal sectioning of the entire spheroid subjected to multiple rounds of deformation. We used MCF10DCIS.com spheroids immunostained with DAPI. The nuclei were segmented using the deep learning-based cell segmentation method Cellpose, using the cyto2 model. A set of quantitative geometric and biological features were extracted, including area, perimeter and aspect ratio, Excess of Perimeter (EOC), as well as the intensity of the ATF3 signal. Specifically, within the segmented nuclear boundaries, we extracted the mean intensity of the ATF3 signal. To determine whether nuclei were positive or negative for ATF3, we compared the mean ATF3 intensity to a defined cutoff set at 8. The secondary background control average intensities were subtracted from the ATF3 intensity signals. Periphery nuclei have centroids ≤ 6.5 μm from the spheroid border. Central nuclei have centroids at a distance > 6.5 μm from the border.

3D segmentation
3D segmentation of the spheroids was performed using Arivis software. Z-stacks of the spheroids were obtained using a confocal laser scanning microscope (CLSM) Leica TCS SP8 and 0.36 μm step size. Z-compensation was applied. MCF10DCIS.com spheroids were transfected with GFP-H2B for visualization of the nuclei. Nuclear count and segmentation are performed with ARIVIS (arivis Pro 4.2.2) using a custom pipeline. For the spheroid segmentation, we used the Intensity Threshold Segmenter operation, while for the nuclei segmentation, we applied the Cellpose-based Segmenter operation with the Cyto2 model. The interstitial volume fractions were subtracted from the total spheroid volume. The average cell volumes were obtained by dividing the total spheroid volume by the number of nuclei. The shape index was calculated for each nuclei[3]:

$$Shape\ index = \frac{surface\ area}{volume^{\frac{2}{3}}}$$

Quantification of spheroid interstitial volume. Control spheroids were incubated with 2 g/L dextran for 20 minutes. Confocal images were obtained using a Leica TCS SP8-STED confocal microscope and taking z-stacks with 0.8 µm step sizes. An automated Fiji macro was applied to each z-slice and the images were processed with Tubeness filter (sigma parameter =1) that identifies the dextran signals in the interstitial spaces. The macro calculates the area of the connected dextran signals and the area of the spheroid per z-slice. Dextran Volume and Spheroid Volume were estimated by interpolating the respective masks along the Z-stack using "3d Object Counter" Fiji plugin for 10 control spheroids.
The percentage of interstitial volume is obtained as the ratio of the interstitial volume V_I: total spheroid volume $V_{spheroid}$ (Figure S6f):

$$Percent\ interstitial\ volume\ (\%) = \Phi_{ECM} = \frac{V_I}{V_{spheroid}} x\ 100$$

Transcriptomics analyses
MCF10.DCIS.com spheroids were introduced into the deformation device and cycled 4 times. The spheroids were then cultured in 6-well low attachment dishes (Corning) for 1 hour and then collected, and washed with PBS. Total RNA from cells was extracted using RNeasy Plus Mini Kit (Qiagen). RNA was quality controlled on Agilent Bioanalyzer 2100, assay class eukaryote total RNA picogram sensitivity. RNA integrity numbers were >9.6 for all samples analyzed. RNA concentrations were quantified using Qubit (broad range, Thermofisher).
Sequencing was performed by GENEWIZ sequencing company (Germany). FASTQ reads were quality checked with FastQC (v 0.11.9) and adaptors trimmed with Trimmomatic (v 0.40)[4] specifying default parameters. High-quality trimmed reads were mapped to the Hg38 reference genome with STAR aligner (v 2.7.9) using the latest GENCODE main annotation file. The construction of the gene count matrix was done using feature Counts[5] on bam files generated by STAR, counting the reads associated to 'exons' features per gene. These steps were performed using nf-core rnaseq pipeline (v 3.13.2)[11].
Differential gene expression on the normalized gene count matrix was performed with DESeq2 (v 3.13). DEGS (differentially expressed genes) were extracted by filtering on P-value adjusted (using 0.05 as threshold value), corrected by the Benjamini Hochberg test. GSEA analysis was performed with fgsea[6] using the Hallmarks pathway gene sets in the GSEA Molecular Signatures Database; only over-represented categories with corrected P-value were kept, using the previously described method. Downstream statistics and Plotting were performed within the R (v 4.0.1) environment.
RNA sequencing experiments were performed in triplicate (each experiment consisted of processing 1000 spheroids per condition). Only 2 repeats were plotted for statistical reasons.

Spheroid composition: ECM and single cells contributions

The relative concentrations of cells and ECM in the different spheroid configurations can be quantified by Raman spectral analysis. The peak areas are proportional to the mass fraction of the chemical species within the scattering volume and the scattering efficiency ε of the corresponding molecular vibration. We define the ratio between the high-frequency peak areas corresponding to CH2-CH3 stretching and OH stretching as:

$$r = \frac{w_{OH}\varepsilon_{OH}}{w_{CH}\varepsilon_{CH}} = \frac{[w_{cell}\, w_{cell\ OH} + (1 - w_{cell})w_{ECM\ OH}]\varepsilon_{OH}}{[\,w_{cell}\,(1 - w_{cell\ OH}) + (1 - w_{cell})(1 - w_{ECM\ OH})]\varepsilon_{CH}}$$

Where w_{cell} is the fraction of cells inside the spheroid respect to the total spheroid which includes cells + ECM, while $w_{cell\ OH}$, and $w_{ECM\ OH}$ are the water fraction of, respectively, cells and ECM. The value r is experimentally accessible from the calculation on the Raman peak areas. By comparing this ratio in different conditions, quantitative data about the relative changes of composition can be estimated.
For example, in the case of the ROCK inhibitor treatment:

$$R = \frac{r_{ROCK}}{r_{ctrl}} = \frac{[\, w_{cell\,ROCK}\, w_{cell\,OH} + (1 - w_{cell\,ROCK}\,)w_{ECM\,OH}]}{[w_{cell\,ROCK}(1 - w_{cell\,OH}) + (1 - w_{cell\,ROCK}\,)(1 - w_{ECM\,OH})]}\, \frac{[w_{cell\,ctrl}(1 - w_{cell\,OH}) + (1 - w_{cell\;CTRL})(1 - w_{ECM\,OH})]}{[w_{cell\;CTRL} w_{cell\,OH} + (1 - w_{cell\;CTRL})w_{ECM\,OH}]}$$

Where $w_{cell\,OH} = 70\%$[7], $w_{CELL\,CTRL} = 86\%$ (evaluated from the volume fraction $\Phi_{CELL\,CTRL}$ extracted in Figure S6). As for the ECM, we assumed $w_{ECM\,OH} = 1$. Given the highly hydrated nature of the ECM, $w_{ECM\,CH} = 1 - w_{ECM\,OH}$ is expected to fall within the 0–10% range[8].

Variations within this range would introduce an error of less than 1% in the obtained mass fraction and thus can be neglected. Under the assumption that the relative dry mass and water content of cells remain constant, and using R calculated from the Raman experimental data, we can evaluate $\Phi_{cell\,ROCK} = 76\%$ and $\Phi_{ECM\,ROCK} = 1 - \Phi_{\mathrm{cell\,ROCK}} = 24\%$. These values, expressed in volume fraction, are derived from mass fraction values.
All the conversions from mass fraction to volume fraction and vice versa have been evaluated using density, according to: $\Phi_i = w_i \rho_{sph} / \rho_i$. The densities used were $\rho_{ECM} = 1.00\, g/cm^3$, $\rho_{cell} = 1.08\, g/cm^3$ [9, 10], while ρ_{sph} was estimated assuming a linear mixture model of cells and ECM $\rho_{sph} = \Phi_{\mathrm{cell}}\, \rho_{cell} + \Phi_{\mathrm{ECM}}\, \rho_{ECM}$. This approach can be applied to compare the control data to any other configuration.

Statistical analyses for ANCOVA
To account for any size-dependent effect of spheroids on the channel 1 transit time in Figure 2a, we performed an ANCOVA analysis (analysis of covariance) between control, ROCK-inhibitor treated and RhoA-activator treated spheroids. This analysis compares the different regression coefficients between groups. The treatment groups (control, ROCK inhibitor and RhoA activator) were the categorical variable, the spheroid diameter is the covariate and the dependent variable is the transit time. We asked whether the transit time can be influenced by the spheroid diameters between groups.

The following table was generated from the analysis: F-value refers to the statistical F-test that compares variances, DFn is the degrees of freedom of the numerator of the F-ratio, DFd is the degrees of freedom in the denominator of the F-ratio, np2 is the partial eta squared that measures the strength of association between the spheroid diameter and transit time and the p-value indicates the significance. A low p-value indicates a significant difference in transit time among conditions, accounting for variations in initial spheroid size.

Table S3: ANCOVA analysis of MCF10.DCIS.com spheroid diameters vs transit times for no-treatment controls, spheroids treated with a ROCK inhibitor or a RhoA activator.

F	DFn	DFd	P value	np2
13.13	2	214	4.18E-06	0.1093

References

1. Jaiswal, D. et al. Stiffness analysis of 3D spheroids using microtweezers. *PLoS One* **12**, e0188346 (2017).
2. Frohlich, E. et al. Comparison of two in vitro systems to assess cellular effects of nanoparticles-containing aerosols. *Toxicol In Vitro* **27**, 409-417 (2013).
3. Sharp, T.A., Merkel, M., Manning, M.L. & Liu, A.J. Inferring statistical properties of 3D cell geometry from 2D slices. *PLoS One* **14**, e0209892 (2019).
4. Bolger, A.M., Lohse, M. & Usadel, B. Trimmomatic: a flexible trimmer for Illumina sequence data. *Bioinformatics* **30**, 2114-2120 (2014).
5. Liao, Y., Smyth, G.K. & Shi, W. featureCounts: an efficient general purpose program for assigning sequence reads to genomic features. *Bioinformatics* **30**, 923-930 (2014).
6. Korotkevich, G. et al. Fast gene set enrichment analysis. *bioRxiv*, 060012 (2021).
7. Fulton, A.B. How crowded is the cytoplasm? *Cell* **30**, 345-347 (1982).

8. Fan, D., Creemers, E.E. & Kassiri, Z. Matrix as an interstitial transport system. *Circ Res* **114**, 889-902 (2014).
9. Antonacci, G. & Braakman, S. Biomechanics of subcellular structures by non-invasive Brillouin microscopy. *Sci Rep* **6**, 37217 (2016).
10. Scarcelli, G. et al. Noncontact three-dimensional mapping of intracellular hydromechanical properties by Brillouin microscopy. *Nat Methods* **12**, 1132-1134 (2015).
11. Harshil Patel, Phil Ewels, Jonathan Manning, Maxime U Garcia, Alexander Peltzer, Rickard Hammarén, Olga Botvinnik, Adam Talbot, Gregor Sturm, nf-core bot, Matthias Zepper, Denis Moreno, Pranathi Vemuri, Mahesh Binzer-Panchal, Ezra Greenberg, silviamorins, Lorena Pantano, Robert Syme, Gavin Kelly, … Gisela Gabernet. (2024). nf-core/rnaseq: nf-core/rnaseq v3.17.0 - Neon Newt (3.17.0). Zenodo. https://doi.org/10.5281/zenodo.13986791
